# Serotype distribution of *Streptococcus pneumoniae* causing invasive disease in children in the post-PCV era: A systematic review and meta-analysis

**DOI:** 10.1371/journal.pone.0177113

**Published:** 2017-05-09

**Authors:** Evelyn Balsells, Laurence Guillot, Harish Nair, Moe H. Kyaw

**Affiliations:** 1 Usher Institute of Population Health Sciences and Informatics, University of Edinburgh, Medical School, Teviot Place, Edinburgh, United Kingdom; 2 Sanofi Pasteur, Swiftwater, Pennsylvania, United States of America; Public Health England, UNITED KINGDOM

## Abstract

**Background:**

Routine immunisation with pneumococcal conjugate vaccines (PCV7/10/13) has reduced invasive pneumococcal disease (IPD) due to vaccine serotypes significantly. However, an increase in disease due to non-vaccine types, or serotype replacement, has been observed. Serotypes’ individual contributions to IPD play a critical role in determining the overall effects of PCVs. This study examines the distribution of pneumococcal serotypes in children to identify leading serotypes associated with IPD post-PCV introduction.

**Methods:**

A systematic search was performed to identify studies and surveillance reports (published between 2000 and December 2015) of pneumococcal serotypes causing childhood IPD post-PCV introduction. Serotype data were differentiated based on the PCV administered during the study period: PCV7 or higher valent PCVs (PCV10 or PCV13). Meta-analysis was conducted to estimate the proportional contributions of the most frequent serotypes in childhood IPD in each period.

**Results:**

We identified 68 studies reporting serotype data among IPD cases in children. We analysed data from 38 studies (14 countries) where PCV7 was administered and 20 (24 countries) where PCV10 or PCV13 have been introduced. Studies reported early and late periods of PCV7 administration (range: 2001∓13). In these settings, serotype 19A was the most predominant cause of childhood IPD, accounting for 21.8% (95%CI 18.6∓25.6) of cases. In countries that have introduced higher valent PCVs, study periods were largely representative of the transition and early years of PCV10 or PCV13. In these studies, the overall serotype-specific contribution of 19A was lower (14.2% 95%CI 11.1∓18.3). Overall, non-PCV13 serotypes contributed to 42.2% (95%CI 36.1∓49.5%) of childhood IPD cases. However, regional differences were noted (57.8% in North America, 71.9% in Europe, 45.9% in Western Pacific, 28.5% in Latin America, 42.7% in one African country, and 9.2% in one Eastern Mediterranean country). Predominant non-PCV13 serotypes overall were 22F, 12F, 33F, 24F, 15C, 15B, 23B, 10A, and 38 (descending order), but their rank order varied by region.

**Conclusion:**

Childhood IPD is associated with a wide number of serotypes. In the early years after introduction of higher valent PCVs, non-PCV13 types caused a considerable proportion of childhood IPD. Serotype data, particularly from resource-limited countries with high burden of IPD, are needed to assess the importance of serotypes in different settings. The geographic diversity of pneumococcal serotypes highlights the importance of continued surveillance to guide vaccine design and recommendations.

## Introduction

*Streptococcus pneumoniae* is the major cause of serious invasive diseases such as bacterial pneumonia, septicaemia, and meningitis in young children worldwide. An estimated 14.5 million cases of invasive pneumococcal disease (IPD) occurred globally in 2000 before introduction of pneumococcal conjugate vaccines (PCVs). Widespread availability of PCVs has reduced the burden of IPD substantially, from over 800,000 annual deaths before PCV introduction to 541,000 deaths in 2008 [1, 2]. Although two formulations of PCVs are available to protect against disease, *S*. *pneumoniae* still poses a significant burden on individuals and healthcare systems.

PCVs have been largely effective in reducing IPD associated with serotypes included in currently available formulations. Yet, concerns exist about their long-term effects since these contain a limited number of serotypes and the potential role of non-vaccine serotypes. The first PCV, licensed in 2000, offered protection against 7 serotypes (4, 6B, 9V, 14, 18C, 19F, 23F). By 2015, PCV7 had been replaced, and PCV10 (PCV7 + 1, 3, 7F) or PCV13 (PCV10 + 19A, 6A, 3) have been introduced in over 130 countries [3, 4]. Before the introduction of PCVs, vaccine-targeted serotypes were associated with a 82∓88% of IPD in North America, 72∓88% in Europe, 68∓79% in Oceania, 58∓82% in Latin America, 49∓77% in Africa, and 52∓74% in Asia [5]. After the introduction and scale up of PCV7 in routine immunisation programmes, IPD caused by these 7 serotypes decreased significantly in children and other age groups [6, 7]. Nasopharyngeal carriage of *S*. *pneumoniae*, which can lead to invasive disease [8], was also dramatically impacted by PCVs. Studies indicated that PCVs reduced the prevalence of targeted serotypes among carriers [9] to the extent that these strains have nearly disappeared in some settings in both the vaccinated and unvaccinated persons [10].

In light of global efforts for universal immunisation with PCV, it is important to examine which serotypes are associated with IPD in the post-PCV era to gain insights into the evolving epidemiology of *S*. *pneumoniae*. Serotype replacement, an increase in incidence of disease due to serotypes [8] not included in PCV7, was noticed among young children using data from 21 large surveillance systems [11]. In these industrialised settings, by the 7^th^ year of PCV7, the rate ratio of vaccine-related IPD (RR 0.03 95%CI 0.01∓0.10) decreased substantially as compared to the ratio of non-PCV7 types (RR 2.81 95%CI 2.12∓3.71). Thus, there is a clear need for close monitoring of serotypes causing IPD to inform public health actions [8]. We aimed to systematically assess and describe the global distribution of serotypes causing IPD in young children after the introduction of PCVs to better understand the contribution of individual serotypes, including non-vaccine types and inform recommendations for vaccine use.

## Methods

### Search methods and inclusion criteria

We conducted a systematic review of the literature according to the PRISMA guidelines. We searched electronic medical databases for studies and surveillance reports (published between 1 January 2000 and 31 December 2015) reporting serotype data from children with IPD after the introduction of a PCV in the study area. Search strategies with database-specific MeSH and free search terms were developed with the assistance of a medical librarian for Medline, Embase, and Global Health (via Ovid); EMRO, SEARO, and WPRO regional databases (Global Health Library), LILACS, and Web of Science. Strategies were designed to retrieve records that included the following terms: *Streptococcus pneumoniae* and, IPD or syndromes, and vaccines, and serotypes (search strategies are available in S1 Table).

We included peer-reviewed studies and annual surveillance reports from Department of Health websites from countries from which the electronic medical databases retrieved national surveillance publications. IPD was defined as the identification of *S*. *pneumoniae* from a normally sterile site (e.g. blood, cerebrospinal, pleural effusions, or joint fluid) in children. No language restrictions or publication type were imposed at this stage. We also scanned the reference list of included studies to identify any additional studies. Eligibility criteria were as described in Box 1, which were modified from previous reviews assessing the serotype distribution in childhood IPD [5, 11].

**Box 1. Eligibility criteria for this review**

**Inclusion**

- Observational studies or annual national surveillance reports from selected Departments of Health from settings with PCV uptake of at least 25% during the study period described
- Studies reporting at least 20 serotyped isolates overall and with at least 50% of reported IPD cases were serotyped
- Study reports data on different serogroups/types for invasive disease (defined as isolates from normally sterile sites) in children
- Study population was representative of the general population, not a selected group with specific co-morbidities
- Surveillance conducted for at least 12 continuous months

**Exclusion**

- Case reports, narrative reviews, quarterly or province-level surveillance reports, if annual and/or national were available
- Serotype data from studies with high risk of bias: e.g. studies focused on serotypes from severe cases, most frequent types, or antimicrobial resistance
- Data for years after PCV introduction are not extractable independently or the study does not provide a description of PCV use in the area
- Data only reported for serotypes included in PCVs (PCV7, PCV10, PCV13)
- Serotype data included isolates obtained from non-sterile sites (e.g. nasopharynx) or not extractable specifically for otherwise healthy children (i.e. study population includes all immunocompromised population or adults)
- Data overlap with other studies included in the analysis (*Studies with the longest study period or larger sample size were preferred)*

**Serotypes in Pneumococcal conjugate vaccines**

**PCV7**: 4, 6B, 9V, 14, 18C, 19F, 23F
**PCV10**: PCV7 + 1, 5, 7F
**PCV13**: PCV10 + 3, 6A, 19A
**Non-PCV13**: those not included in PCV13

### Data collection and analysis

Two reviewers (EB, LG) independently reviewed identified publications and extracted relevant data into a template (Microsoft Excel), which was piloted before use. Datathief III software (http://datathief.org/) was used to extract data from images. Study periods were classified according to the PCV administered during the study period as either PCV7 or higher valent (PCV10 or PCV13, hereafter PCV10/13). From each study included in analysis, data for young children (<5 years) was preferred, if other age groups were available to minimise bias. Non-vaccine types were those currently not included in PCV13 (non-PCV13).

#### Meta-analysis

Serotype-specific percentages were calculated for each study (% serotype *x* = total number of cases serotype *x* over total IPD isolates with serotype data x 100). Serogroup data were not redistributed into serotypes. Using *metan* in Stata 13 (Statacorp, College Station, TX), pooled estimates and 95% confidence intervals (95%CI) were calculated for serotype-specific proportions using the transformed log of the proportion and random effects model (DerSimonian-Laird method). A continuity correction was used to include data from studies with zero counts in independent meta-analyses. If there were multiple studies from the same setting, the most representative of childhood IPD (i.e. larger sample size, or reporting data for any IPD rather than a single syndrome) was included in meta-analysis. Most data for settings where PCV10 or PCV13 have been introduced were from countries that had transitioned from PCV7, except Brazil, Chile, and Colombia. The first year of PCV10 or PCV13 introduction for these 3 Latin American countries was excluded from analyses to allow time for scaling up of vaccine uptake. For other settings, data from the transition year from PCV7 to higher valent PCVs was included in meta-analysis, as it was not possible to exclude this year from analysis for all studies. We used data exclusively for years with either PCV7 or the current higher valent PCV, except when disaggregation was not possible. We report overall and regional pooled estimates with 95%CIs for vaccine types and individual non-PCV13 types causing at least 1% of IPD in the dataset of countries that have introduced PCV10 or PCV13.

## Results

### Literature review

**Fig 1:**
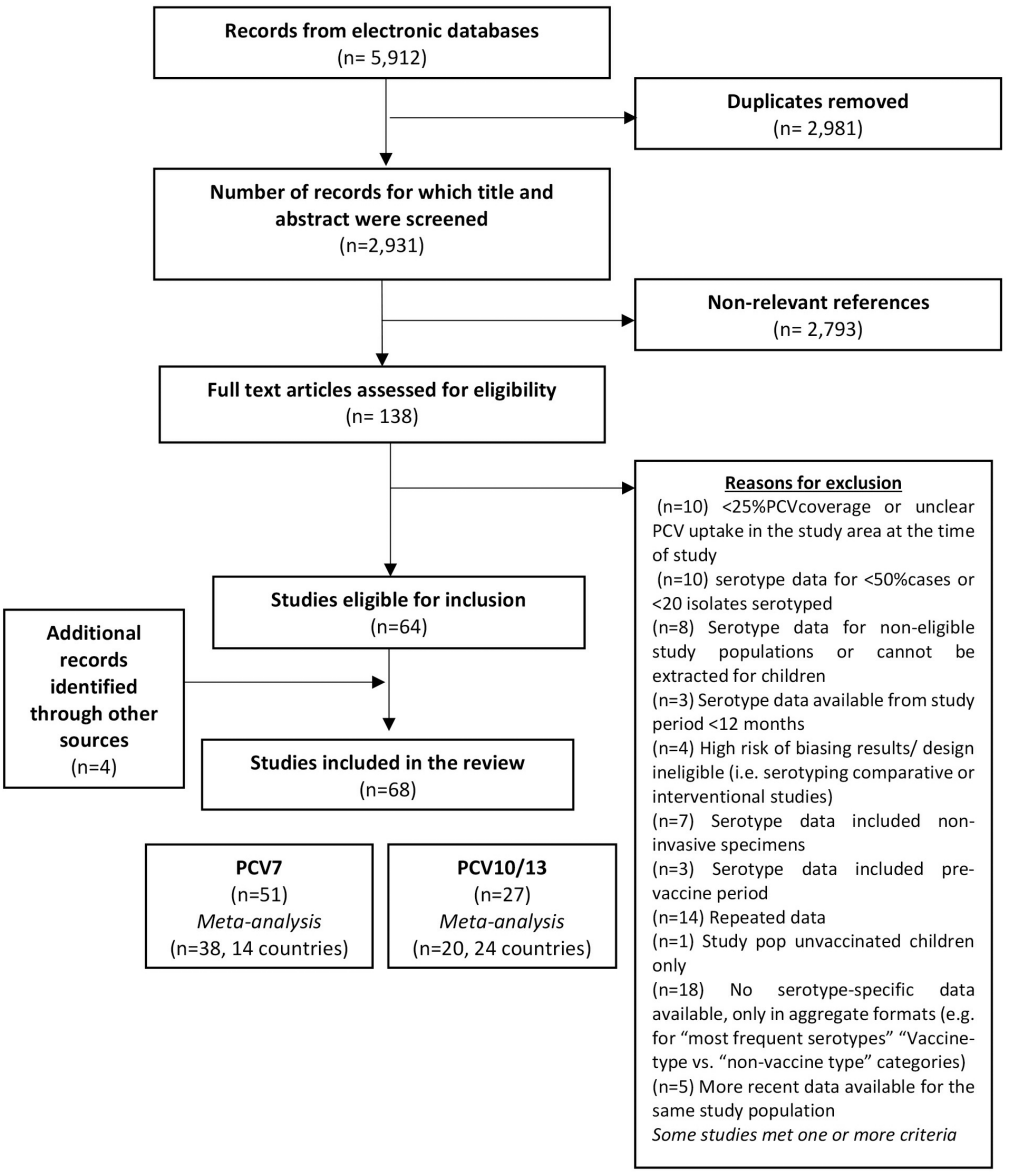
PRISMA flowchart—Literature review process.

We identified 5,912 records through databases search and identified 138 articles for full text examination (Fig 1). Of these, 64 [6, 7, 12–73] articles met our pre-defined eligibility criteria and we identified an additional 4 surveillance reports [74–77] from online searches.

### Characteristics of included publications

A total of 27 countries were represented in the 68 publications included in the review. Surveillance data were retrieved from online reports for Latin America (SIREVA II reports 2009–12, considered as 1 publication), Scotland (UK), Australia, New Zealand, and Singapore. Studies identified overlapping geographic, time period or for individual clinical syndromes were excluded from meta-analyses (details in S2 Table).

Main characteristics of studies included in meta-analysis are available in Table 1. Eight publications reported serotype data for years after both time periods after PCV7 and PCV10/13 implementation. These were extracted separately for analysis. After classification of the studies, we analysed 67 data points assessing childhood IPD, primarily from North America (n studies = 17), Europe (n = 28), and Latin America (n = 11), but also from Western Pacific (n = 8), Eastern Mediterranean (n = 2) and Africa (n = 1).

**Table 1:** Main characteristics of studies included in the review and meta-analyses.

Last author (publication year)	Country	PCV	Introduction year	Study years (PCV uptake)	Isolates	PCV13 types (%)	Non-PCV13 (%)
Rendi-Wagner (2009)	Austria	PCV7	2001*	2005–06 (25%)	36	97.2	2.8
Lepoutre (2015)	France	PCV7	2003*, 2006†	2008–09 (83–95%)	483	71.4	28.6
Varon (2015)	France	PCV7	2006†	2008–09 (>86%)	388	67.5	32.5
van der Linden (2015)	Germany	PCV7	2006†	2007–10 (80–85%)	542	68.1	31.9
Knol (2015)	Netherlands	PCV7	2006†	2008–11 (94–95%)	128	52.3	47.7
Steens (2013)	Norway	PCV7	2006†	2006–10 (86–92%)	165	69.1	30.9
Aguiar (2008)	Portugal	PCV7	2001*	2003–05 (33%)	90	85.6	14.4
Aristegui (2007)	Spain	PCV7	2001*	2002–03 (28–45%)	77	88.3	11.7
Barricarte (2007)	Spain	PCV7	2001*	2001–05 (45%)	85	87.1	12.9
Calbo (2006)	Spain	PCV7	2001*	2002–04 (34%)	64	78.1	21.9
Guevara (2014)	Spain	PCV7	2001*	2004–09 (25–61%)	106	78.3	21.7
Munoz-Almagro (2011)	Spain	PCV7	2001*	2009§	130	80.0	20.0
Perez-Trallero (2009)	Spain	PCV7	2001*	2002–07 (~50%)	45	86.7	13.3
Picazo (2011)	Spain	PCV7	2001*	2007–09 (~50%)	330	79.1	20.9
Rodriguez (2011)	Spain	PCV7	2006†	2007–09 (94.5%)	366	74.9	25.1
Salleras (2009)	Spain	PCV7	2001*	2005–07 (35%)	240	77.9	22.1
Vila-Corcoles (2013)	Spain	PCV7	2001*	2002–09 (13–47%)	65	84.6	15.4
Ceyhan (2011)	Turkey	PCV7	2008†	2008–10§	146	77.4	22.6
Miller (2011)	UK	PCV7	2006†	2008–10§	528	69.7	30.3
Moore (2014)	UK	PCV7	2006†	2006–10 (92–87%)	65	49.2	50.8
Parra (2013)	Colombia	PCV7	2009	2010–11 (69.9%)	84	70.2	29.8
Bettinger (2010)	Canada	PCV7	2005†	2006–07 (80–90%)	212	69.8	30.2
De Wals (2012)	Canada	PCV7	2004	2007 (>90%)	113	63.7	36.3
Kellner (2009)	Canada	PCV7	2002	2003-07(87–91%)	67	76.1	23.9
Black (2007)	USA	PCV7	2000†	2001–05 (86%)	84	34.5	65.5
Bruce (2015)	USA	PCV7	2001†	2005–08§	126	61.9	38.1
Byington (2005)	USA	PCV7	2000†	2001–03§	105	74.3	25.7
Croney (2013)	USA	PCV7	2000†	2002–10§	157	60.5	39.5
Hsu (2010)	USA	PCV7	2000†	2001-02/06-07§	130	66.2	33.8
Kaplan (2013)	USA	PCV7	2000†	2007–09§	609	67.7	32.3
Pilishvili (2010)	USA	PCV7	2000†	2006–07§	519	67.9	32.1
Schutze (2004)	USA	PCV7	2000†	2001–03§	75	77.3	22.7
Sharma (2013)	USA	PCV7	2000†	2008–09§	47	59.6	40.4
Weatherholtz (2010)	USA	PCV7	2000†	2001–06§	115	76.5	23.5
Williams (2011)	Australia	PCV7	2001*, 2005†	2006–07§	201	67.2	32.8
Chiba (2014)	Japan	PCV7	2010/11*	2011–12 (50–90%)	302	55.0	45.0
Ishiwada (2014)	Japan	PCV7	2010*, 2011†	2012–13§	33	51.5	48.5
Suga (2015)	Japan	PCV7	2010*	2011–13 (89%)	308	68.8	31.2
von Gottberg (2013)	South Africa	PCV13	2011†	2011–12§	839	57.3	42.7
Al-Sheikh (2014)	Saudi Arabia	PCV13	2010†	2009–12§	78	85.9	14.1
Shibl (2012)	Saudi Arabia	PCV13		2010§	108	94.4	5.6
Varon (2015)	France	PCV13	2010†	2012–13 (>92%)	181	17.7	82.3
van der Linden (2015)	Germany	PCV13	2009 Dic†	2010–14 (80–85%)	567	35.4	64.6
Knol (2015)	Netherlands	PCV10	2011†	2011–14 (94–95%)	57	21.1	78.9
Steens (2013)	Norway	PCV13	2011†	2011–12 (92%)	47	55.3	44.7
Guevara (2014)	Spain	PCV13	2010	2010–13 (78%)	25	52.0	48.0
Moore (2014)	UK	PCV13	2010†	2010–13§	48	37.5	62.5
Scotland Surveillance	UK	PCV13	2010†	2010–15§	206	19.9	80.1
Waight (2015)	UK	PCV13	2010†	2013–14§	247	14.2	85.8
SIREVA (Brazil)	Brazil	PCV10	2010‡	2011–12 (81.5%)	416	68.8	31.3
SIREVA (Chile)	Chile	PCV10	2011‡	2012 (54.0%)	168	72.6	27.4
SIREVA (Colombia)	Colombia	PCV10	2010‡	2011–12 (69.8%)	208	74.0	26.0
SIREVA (Costa Rica)	Costa Rica	PCV13	2011†	2011–12 (78.0%)	38	76.3	23.7
SIREVA (Ecuador)	Ecuador	PCV10	2010†	2011–12 (71.0%)	62	80.6	19.4
SIREVA (El Salvador)	El Salvador	PCV13	2011†	2011–12 (98.1%)	31	74.2	25.8
SIREVA (Mexico)	Mexico	PCV13	2011†	2012 (97.8%)	105	66.7	33.3
SIREVA (Panama)	Panama	PCV13	2011†	2011–12 (61.8%)	68	88.2	11.8
SIREVA (Peru)	Peru	PCV10	2011†	2011–12 (81.9%)	23	69.6	30.4
SIREVA (Uruguay)	Uruguay	PCV13	2010†	2010–12 (92.0%)	96	50.0	50.0
Demczuk (2013)	Canada	PCV13	2010†	2010–12 (74–90%)	886	55.5	44.5
Bruce (2015)	USA	PCV13	2010†	2010–13 (86–96%)	52	25.0	75.0
Kaplan (2013)	USA	PCV13	2010†	2010–11§	283	56.5	43.5
Moore (2015)	USA	PCV13	2010	2012–13 (63–76%)	177	19.2	80.8
Australia Surveillance	Australia	PCV13	2011†	2012§	184	43.5	56.5
Nakano (2015)	Japan	PCV13	2013†	2014§	126	28.6	71.4
N Zealand Surveillance	New Zealand	PCV13	2014	2014–15 (93%)	78	51.3	48.7
Singapore Surveillance	Singapore	PCV13	2010* 2011†	2012–14§	65	86.2	13.8

Notes—Definitions for PCV uptake varied by study. Uptake is presented as reported in each study.

*PCV was first recommended, approved or licenced,

^†^Included in national immunisation programmes/Universal administration recommended

^‡^ First year of PCV implementation in the country. Settings where national or universal PCV administration is recommended, where eligible for inclusion.

^§^Data not reported.

We analysed serotype data from 7,366 IPD isolates (range: 33∓609) from 38 studies in 14 countries for study periods when PCV7 was administered (Table 1). Study periods ranged from 2001, in the USA [19, 21, 38, 61, 72] until 2013 in Japan [24, 40, 66]. We excluded studies reporting <25% PCV coverage, but studies included serotype data from different uptake levels. For instance, from settings where PCV7 had been recently licensed, introduced into national immunisation programmes, or it was largely available through the private system, for instance, in Spain [16, 17, 22, 34, 52, 56, 57, 59, 60, 69], Portugal [12], and Austria [58]. Other studies were from settings where vaccine uptake was high or PCV was recommended universally in the study setting, such as in Canada [18, 26, 43], USA, UK, Colombia (Bogota) [55], France [13].

We analysed 20 publications from 24 countries where PCV10 or PCV13 have been introduced. The Americas region was the most represented (n = 12 countries). PCV10 was the primary vaccine in 6 countries (Brazil, Chile, Colombia, Ecuador, Peru, and the Netherlands), while PCV13 had been introduced in the remaining 18. Most study periods included the year of transition from PCV7 or the year after introduction of the current higher valent. PCV. A total of 5,469 IPD isolates with serotype data were included in meta-analysis. The number of isolates in individual studies ranged from 23 to 886. The countries with the largest number of isolates were Canada and South Africa (n = 886 and 839, respectively). Six publications reported data for fewer than 50 isolates and an additional 8 reported less than 100 cases.

### Serotype distribution among paediatric IPD cases following PCV introduction

#### PCV-targeted serotypes

**Fig 2:**
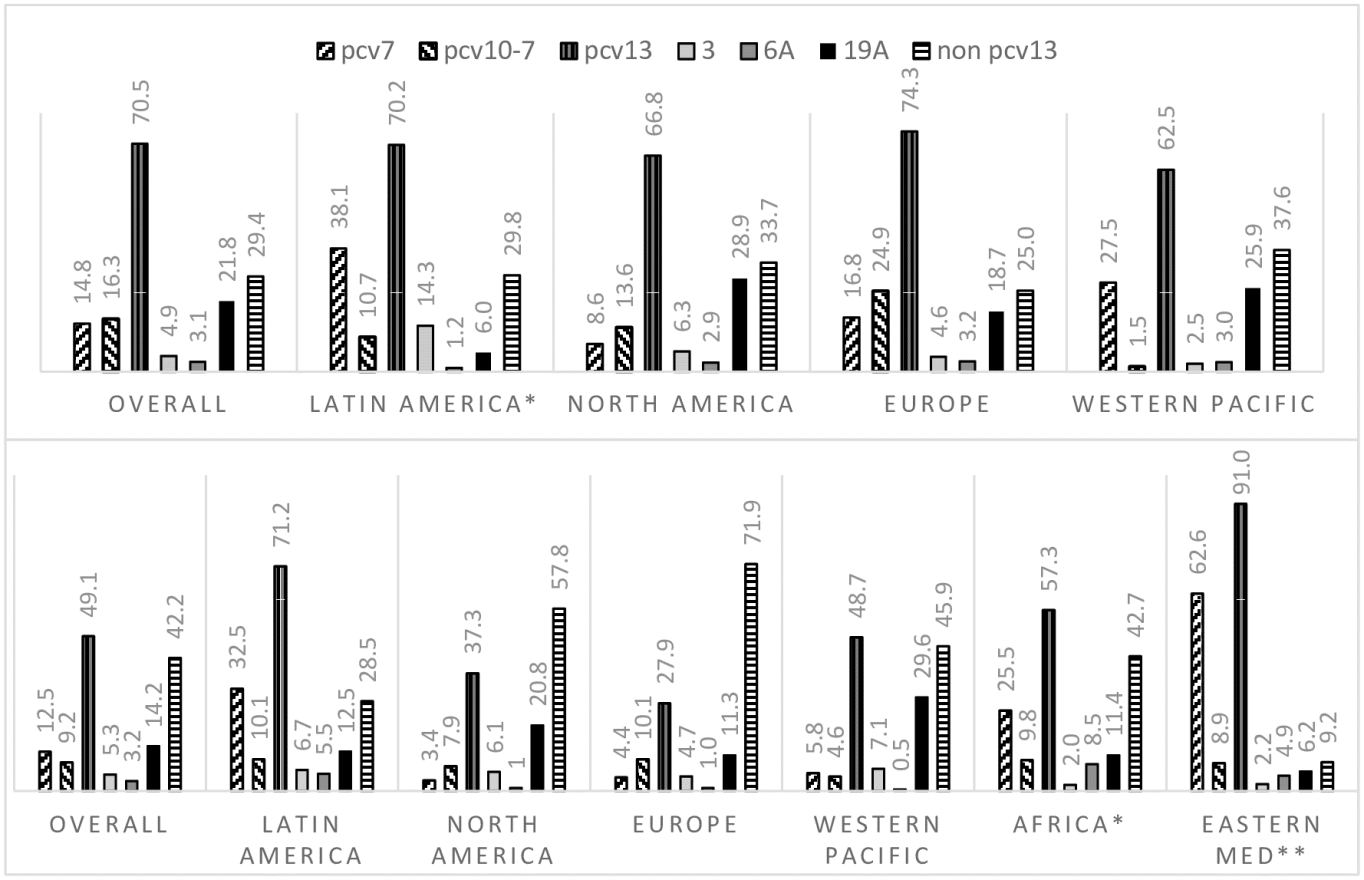
Estimates of serotype-specific contributions to IPD in children (%, 95%CI) reported are based on random effects model from meta-analysis of 3 or more studies, unless indicated [one study (*) or two (**)].

In studies where PCV7 was administered, serotypes included in the heptavalent conjugate vaccine accounted for an overall 14.8% (95%CI 11.4∓19.1) of childhood IPD cases. In studies with study periods after higher valent PCVs were introduced, the proportional contribution of these seven serotypes to IPD in children was 12.5% (95%CI 8.8∓17.7). In the latter, all countries, except the Netherlands, identified PCV7 types. PCV7 types were most frequently isolated from childhood IPD cases in Latin America, Africa, and Eastern Mediterranean regions (32.5%, 25.5%, 62.6% of IPD cases, respectively). Conversely, PCV7 types accounted for lower proportions of IPD in North America, Europe, and the Western Pacific, between 3.4∓5.8%, after introduction of PCV10 or PCV13. Fig 2 and Tables 2 and 3 show the overall meta-estimate and regional stratifications results per period.

**Table 2:** Serotype-specific contributions (%) to paediatric IPD cases by region in studies with PCV7 implementation.

	OVERALL (N = 38)	LATIN AMERICA (N = 1)		NORTH AMERICA (N = 13)	EUROPE (N = 20)	WESTERN PACIFIC (N = 4)	
**PCV7**	**14.8 (11.4–19.1)**	*	**38.1 (26.9–53.9)**	**8.6 (4.9–15.2)**	**16.8 (11.9–23.9)**		**27.5 (19.8–38)**
**PCV10-7**	**16.3 (13.5–19.8)**	*	**10.7 (5.6–20.6)**	**13.6 (10.8–17)**	**24.9 (20.7–30)**		**1.5 (0.8–2.9)**
**PCV13**	**70.5 (67.6–73.5)**	*	**70.2 (54.4–90.7)**	**66.8 (62.3–71.6)**	**74.3 (70.6–78.2)**		**62.5 (54.9–71.3)**
**19A**	21.8 (18.6–25.6)	*	6 (2.5–14.3)	28.9 (23–36.4)	18.7 (15.4–22.7)		25.9 (17.7–37.9)
**3**	4.9 (4.2–5.8)	*	14.3 (8.1–25.2)	6.3 (5.1–7.8)	4.6 (4–5.4)		2.5 (1.4–4.6)
**6A**	3.1 (2.4–4.1)	*	1.2 (0.2–8.5)	2.9 (1.5–5.6)	3.2 (2.2–4.5)		3 (2–4.5)
**NON PCV13**	**29.4 (26.8–32.4)**	*	**29.8 (20.1–44)**	**33.7 (29.4–38.7)**	**24.9 (21.7–28.8)**		**37.6 (30.1–46.9)**
**22F**	3.5 (2.9–4.3)	*	1.2 (0.2–8.5)	4.9 (4–6)	2.2 (1.5–3.3)		4 (2.8–5.6)
**12F**	2.3 (1.8–2.9)		−	3.1 (1.6–6)	1.9 (1.5–2.4)	*	0.3 (0–2.3)
**33F**	3.4 (2.6–4.6)		−	4.8 (3.7–6.3)	3 (1.7–5.1)		2.1 (1.3–3.4)
**24F**	4 (3.4–4.8)	*	1.2 (0.2–8.5)	−	3.3 (2.5–4.5)	**	5 (3.1–8)
**15C**	2.8 (2.3–3.4)	*	2.4 (0.6–9.5)	2.6 (1.7–3.8)	2.2 (1.7–2.9)		4.7 (3.3–6.7)
**15B**	2.4 (2–3)	*	3.6 (1.2–11.1)	2.4 (1.6–3.5)	2.3 (1.8–3)	**	2.8 (1.8–4.6)
**23B**	1.7 (1.4–2.1)	*	1.2 (0.2–8.5)	2 (1.4–2.8)	1.5 (1.1–2)		−
**10A**	2.6 (2–3.3)		−	2.5 (1.7–3.5)	2.5 (1.6–3.8)		2.5 (1.2–5.4)
**38**	2.3 (1.8–2.8)		−	2.7 (1.8–4.2)	1.9 (1.4–2.7)		1.8 (1–3.2)
**15A**	1.8 (1.2–2.7)	*	2.4 (0.6–9.5)	1.5 (1–2.3)	1.1 (0.8–1.6)		7.1 (5–10.2)
**35B**	1.2 (0.9–1.6)	*	2.4 (0.6–9.5)	1.5 (0.9–2.6)	0.7 (0.4–1.1)		1.6 (0.8–3.1)
**6C**	2.8 (2.2–3.4)		−	3.4 (2.4–4.8)	1.1 (0.7–1.7)	**	4.2 (2.7–6.8)
**8**	1.4 (1.1–1.9)	*	2.4 (0.6–9.5)	0.9 (0.5–1.8)	1.6 (1.1–2.2)		−
**11A**	1.3 (1–1.8)	*	2.4 (0.6–9.5)	1.1 (0.6–2)	1.2 (0.8–1.7)	**	1.8 (0.7–4.5)
**23A**	1.6 (1.2–2.1)		−	1.8 (1.2–2.7)	0.7 (0.4–1.2)	**	1.4 (0.2–9.1)
**9N**	0.9 (0.6–1.2)		−	0.7 (0.4–1.3)	1 (0.6–1.7)		−

Estimates of serotype-specific contributions to IPD in children (% 95%CI) reported are based on random effects model from meta-analysis of 3 or more studies, unless indicated [one study (*) or two (**)].–indicates no data reported. Meta-analyses were conducted individually, thus the sum of each of the serotypes in a category may exceed 100%

**Table 3:** Serotype-specific contributions (%) to paediatric IPD cases in settings that have introduced PCV10 or PCV13.

	OVERALL (N = 20)	LATIN AMERICA (N = 1†)		NORTH AMERICA (N = 4)		EUROPE (N = 8)	WESTERN PACIFIC (N = 4)		AFRICA (N = 1)		EASTERN MEDITERRANEAN (N = 2)	
**PCV7**	**12.5 (8.8–17.7)**		**32.5 (25.5–41.3)**		**3.4 (2.5–4.5)**	**4.4 (3.3–5.7)**		**5.8 (1.6–21.1)**	*	**25.5 (22.3–29.2)**	**	**62.6 (46.3–84.7)**
**PCV10-7**	**9.2 (6.9–12.2)**		**10.1 (5–20.4)**		**7.9 (4.5–13.9)**	**10.1 (6.6–15.4)**		**4.6 (1.5–14)**	*	**9.8 (7.9–12.2)**	**	**8.9 (1–81.8)**
**PCV13**	**49.1 (42.3–56.9)**		**71.2 (65.9–77)**		**37.3 (25.4–54.7)**	**27.9 (19.9–39.1)**		**48.7 (31.6–74.8)**	*	**57.3 (52.4–62.6)**	**	**91 (78.2–100)**
**19A**	14.2 (11.1–18.3)		12.5 (7.7–20.2)		20.8 (13.3–32.6)	11.3 (7.6–17)		29.6 (20.3–43.1)	*	11.4 (9.3–13.9)	**	6.2 (3.4–11.2)
**3**	5.3 (4.2–6.7)		6.7 (4.7–9.7)		6.1 (3.9–9.3)	4.7 (3.6–6.1)		7.1 (3.4–14.9)	*	2 (1.3–3.2)	*	2.2 (0.8–5.8)
**6A**	3.2 (2.1–4.9)		5.5 (4.2–7.1)	**	1 (0.6–1.8)	1 (0.5–2)	*	0.5 (0.1–3.8)	*	8.5 (6.8–10.8)	**	4.9 (2.2–11.3)
**NON-PCV13**	**42.2 (36.1–49.5)**		**28.5 (23.4–34.7)**		**57.8 (41.6–80.4)**	**71.9 (63.1–82)**		**45.9 (30.9–68.2)**	*	**42.7 (38.5–47.4)**	*	**9.2 (3.7–22.9)**
**22F**	5.3 (4.2–6.7)		2.3 (1.4–3.7)		6.9 (4.5–10.4)	5.2 (3.6–7.5)		7.4 (4.6–12)		−		−
**12F**	4.3 (3.5–5.3)		4 (2.8–5.7)	**	3.3 (1.3–8.3)	5.6 (4.4–7)		−	*	4 (2.9–5.6)	*	1.3 (0.2–9.1)
**33F**	4.5 (3.4–5.9)	**	2 (0.8–4.8)		4.9 (2.2–10.8)	4.9 (3.3–7.4)		4.3 (2.5–7.5)		−		−
**24F**	4.2 (2.6–6.8)		2.4 (1.6–3.8)		−	6.7 (3.8–11.9)	*	3.9 (0.1–12.4)		−		−
**15C**	4 (3.1–5)		1.5 (0.8–2.7)		3.9 (2.4–6.5)	5.9 (4.4–7.8)	*	4.3 (2.2–8.7)		−		−
**15B**	3.7 (3.1–4.4)		2.5 (1.5–4.3)		4.2 (2.3–7.8)	3.7 (2.6–5.2)	**	3.3 (1.7–6.7)	*	4.3 (3.1–5.9)		−
**23B**	3.4 (2.6–4.3)		2.4 (1.4–4.1)		3 (2–4.4)	4.1 (2.6–6.5)	**	3.9 (2.1–7.3)		−	*	1.3 (0.2–9.1)
**10A**	3.4 (2.2–5.4)		1.3 (0.8–2.3)	**	2.6 (1.7–3.8)	6.7 (3.7–12.4)	**	3 (1.6–5.8)		−		−
**38**	3.4 (2.4–4.8)		1.8 (0.6–5.5)	**	5 (1.7–14.3)	3.2 (1.8–5.6)	**	0.7 (0.2–2.6)		−		−
**15A**	2.9 (1.9–4.4)		1.4 (0.8–2.5)		3.3 (1.5–7.4)	3.9 (1.9–7.9)	**	3 (0.2–38.9)		−		−
**35B**	2.6 (1.8–3.8)		1.2 (0.2–6.4)		4.5 (1.7–11.4)	1.4 (0.9–2.3)	**	3.9 (2.2–6.9)		−		−
**6C**	2.4 (1.8–3)		2.4 (1.6–3.7)		2.3 (1.6–3.3)	1.6 (0.7–3.6)		3 (1.2–7.1)		−		−
**8**	2.2 (1.3–3.8)		1.2 (0.6–2.3)	**	1.5 (0.9–2.5)	2.9 (1.3–6.2)	*	3.8 (1.2–11.9)	*	8.3 (6.6–10.5)	*	1.3 (0.2–9.1)
**11A**	2 (1.6–2.6)		2.2 (1.5–3.3)	**	2.5 (1.6–3.7)	1.7 (1.1–2.6)	**	0.7 (0.2–2.6)		−		−
**23A**	2 (1.6–2.6)		2 (1.2–3.6)		2.2 (1.5–3.2)	1.6 (1–2.5)		2.4 (1.1–5)		−	*	2.6 (0.6–10.2)
**9N**	1.3 (1–1.8)		1.3 (0.6–2.8)	**	1.3 (0.7–2.4)	1.3 (0.7–2.4)	**	1.5 (0.5–4.7)	*	1.8 (1.1–3)		−

Estimates of serotype-specific contributions to IPD in children (% 95%CI) reported are based on random effects model from meta-analysis of 3 or more studies, unless indicated [one study (*) or two (**)].–indicates no data reported.

^†^10 countries from the SIREVA surveillance network. Meta-analyses were conducted individually, thus the sum of each of the serotypes in a category may exceed 100%

#### PCV10-specific serotypes

Serotypes 1, 5, and 7F were identified in all studies with periods of PCV7 implementation, accounting for an overall 16.3% (95%CI 13.5∓91.8) of childhood IPD cases. These serotypes comprised 9.2% (95%CI 6.9∓12.2) in settings where PCV10 or PCV13 have been introduced; which were reported in most countries (except France and Singapore). In this most recent period, the contributions to paediatric IPD cases due to PCV10-specific serotypes were similar in all regions (approximately 8∓9%), except in the Western Pacific (4.6%).

#### PCV13-specific serotypes

In both periods, the most frequent PCV13 type was 19A, followed by 3, and then 6A. Serotype 3 accounted for 5∓6% of childhood IPD cases after the introduction of either PCV, while 6A was associated with approximately 3% of all cases. In studies from countries where PCV10 or PCV13 have been introduced, serotype 19A accounted for 14.2% (95% CI 11.1∓18.3%) of childhood IPD cases across all regions (and at least 10% in each of the regions).

#### Non-PCV13

There was wide-variation in the number of non-PCV13 strains reported. In addition to the 16 non-PCV13 types included in meta-analysis, at least 1 case was reported for over 60 different serotypes currently absent in any of the PCV formulations (a list is provided in the S3 Table).

The overall contribution of non-PCV13 types to IPD in children was 29.4% (95%CI 26.8∓32.4) in periods of PCV7 administration and 42.2% (95%CI 36.1∓49.5) in studies where higher valent PCVs have been introduced. In each period, differences between the individual proportional contribution of non-PCV13 serotypes to childhood IPD were small and their overall ranking varied within regions (Tables 2 and 3). Based on data from countries where PCV10 or PCV13 have been introduced, 22F was the most common serotype (5% of overall childhood IPD cases analysed), followed by 12F, 15C, 24F, 33F (4% each). Combined, the leading non-PCV13 serotypes 22F, 12F, 33F accounted for 4∓16% of IPD cases in children across regions, except in the Eastern Mediterranean region. Notably, serotype 24F appears to be prevalent in Europe and Western Pacific regions, but not North America.

### Contributions of serotypes to childhood IPD between periods

We compared the contribution of various serotypes to childhood IPD in the two sets of studies (PCV7 implementation and PCV10 or PCV13 introduction). We observed decreases in the percentage point differences in PCV7 types (-2.3%), PCV10-only strains (-7.1%) and 19A (-7.6%). Changes observed between periods for overall meta-estimates of non-PCV13 types were small. Comparisons between the regions with the longest history of PCV use (North America, Europe, and Western Pacific) show variations in the percentage point reductions in PCV13 serotypes among childhood IPD cases:-29.5%, -46.4%, and -13.8% in North America, Europe, and Western Pacific regions, respectively. Notably, studies from these regions reported a range of early and late periods of PCV7 use in individual study settings, while data from countries that have introduced higher valent PCVs were largely representative of the transition and early years of the vaccines. Non-PCV13 serotype 22F accounted individually for at least 5% of childhood IPD in North America, an increase of approximately 2 percentage points, compared to the post-PCV7 period. Similar changes in this serotype was also seen in Europe and Western Pacific (+3%). Other non-PCV13 serotypes with an increase in their proportional contribution to paediatric IPD after introduction of PCV10/13 differ between regions. For instance, serotype 38 (+2.7%) accounted for more childhood IPD cases in North America while 12F and 15C (+3.7%) and 10A (+4.2%) were more prominent in Europe.

## Discussion

This is the first study to comprehensively report *S*. *pneumoniae* serotype-specific contributions to IPD in young children subsequent to the introduction of PCVs. Our estimates, based on a systematic analysis of data available to-date indicate that, in countries that have introduced higher-valent PCVs, approximately 42% (95%CI 36∓49) of childhood IPD cases were caused by non-PCV13 serotypes. Our results provide insights into the relative importance of serotypes in childhood IPD after widespread implementation of PCVs and highlight regional differences.

It is well documented that routine immunisation with PCVs has led to significant decreases in both colonisation and IPD by vaccine-targeted types in children and that it has indirectly impacted non-targeted populations [78]. It was estimated that PCV7-targeted serotypes accounted for 49∓82% of childhood IPD before PCV introduction [5]. We found that, after PCV7 implementation, their proportional contribution was approximately 11∓19%. This is consistent with other regional estimates, as prior to the introduction of PCV13 in Europe PCV7-type IPD was approximately 19% among children based on data from 26 countries [79]. Subsequent to the introduction of higher valent PCVs, the proportional contribution of serotypes targeted by PCVs continued to decrease, with differences across regions.

Concerns exist about optimum prevention of PCV13-types 3 and 19A−IPD in young children. We estimated that serotype 3−specific contribution to childhood IPD was approximately 5% in the two PCV periods analysed. These estimates are noteworthy, as serotype 3 has been associated with cases of vaccine failure [80]. Our meta-estimates also show the extent of 19A’s predominant contribution to childhood IPD in different world regions after the introduction of PCVs. Subsequent to the introduction of PCV7, 19A was consistently identified as the most frequent serotype associated with childhood IPD cases in the Americas, Europe, and Western Pacific regions (causing approximately 20% of cases). We also found an important contribution to childhood IPD cases of serotype 19A (14.2% 95%CI 11.1∓18.3) in the early years of higher valent PCVs. More recent data suggest different experiences regarding changes in the contribution of 19A at the country level. In the USA, significant reductions of IPD after 5 years of use of PCV13 have been driven by decreases in 19A [81]. However, in other settings in Europe, this serotype continues to pose challenges as a disease-causing strain despite widespread use of PCVs [82, 83]. Differences in vaccine schedules and catch-up campaigns between these settings, as well as time required to observe a decrease in disease due to vaccine-targeted strains are key factors that will influence the epidemiology of *S*. *pneumoniae* strains. The estimated contributions of serotypes to childhood IPD in this study emphasise the need for a better understanding of factors associated with disease due to vaccine types, especially as PCV10 and PCV13 continue to be administered worldwide.

Widespread use of PCVs has resulted in dramatic reductions of IPD associated with vaccine serotypes among young children in high-income countries in North America, Europe, and the Western Pacific [11, 51, 79]. Knowledge of the serotypes that are associated with IPD among the target population is important for the development of new vaccines that extend protection against non-PCV13, which could also affect other age groups. We found that subsequent to the introduction of higher valent PCVs in North America and Europe, approximately half of childhood IPD cases were due to serotypes for which there is no protection via immunisation. Serotypes likely to provide further reductions in disease are 22F, 33F, 15B, 38, and 35B (25% combined) in North America. In Europe, the leading disease-causing non-PCV13 strains were 12F, 10A, 24F, 22F, and 15C, accounting for 30% of IPD. Serotypes 12F, 22F, 24F, and 33F have been identified to have high invasive disease potentials [68, 84, 85]. Studies have also shown that non-PCV13 15B/C/A, 23B, 24F, 35B are not only important causes of IPD but also are common nasopharyngeal colonisers and have high prevalence of antibiotic resistance [86–90]. Considering the important contribution of non-PCV13 serotypes to childhood IPD in these settings, next generation PCVs with greater coverage will be needed to reduce the remaining burden of pneumococcal disease.

There is paucity of data on burden of pneumococcal disease in Latin American countries. Nevertheless, 1,500,000 cases and 28,000 deaths of IPD are estimated to occur annually in children less than 5 years of age in Latin America [91]. We analysed data from 10 countries in Latin America, all from SIREVA surveillance systems, of which 5 currently use PCV10. Compared with other regions, the proportions of IPD caused by vaccine types were highest in this region. The contribution of PCV7 serotypes for IPD was 10-fold higher in Latin America than in the North America. Given the predominant role of vaccine types, the expansion of immunisation programmes with PCVs and surveillance of epidemiological changes is critical for effectively reducing the burden of pneumococcal disease in Latin American countries.

Before PCVs became available, the highest proportion of deaths (60%) associated with childhood IPD were estimated to occur in Africa and Asia [1]. A limited number of countries in the South East Asian and Mediterranean region have introduced PCVs and are evaluating their impact [92]. A recent systematic review of serotypes associated with IPD found that 1, 14, and 19F were common in South Asian countries. Further, serotypes varied across countries and there was low prevalence of serotype 19A [93]. We only included serotype data for two countries from two of these regions—South Africa and Saudi Arabia. In South Africa, serotype 8 was the most frequent serotype in childhood IPD post-PCV. This is in contrast to its position during the pre-PCV era in Africa, when its contribution was approximately 1% of IPD, ranking 8^th^ [5]. Thus, our overall pooled estimates for the so-called African and Eastern Mediterranean regions in this study should be interpreted with caution. Our review of the literature shows that evidence of the effects of PCV in countries in Asia, Africa, and Eastern Mediterranean is urgently needed to further understand the role of serotypes following PCV10 and PCV13 introduction in settings with high burden of *S*. *pneumoniae* disease.

Serotype-specific meta-analysis, as performed in this study, provides evidence on potential serotypes of interest in the post-PCV era. However, changes in the proportional contribution of different strains to childhood IPD need to be interpreted carefully and in the context of changes in IPD incidence in a particular setting. For example, if the percentage of isolates for a serotype doubled, but the IPD decreased by 50%, it would indicate that there was no change in the absolute number of cases for particular serotypes. Without incidence data, we are unable to indicate if there has been a change in the absolute number of IPD cases in any given serotype in the post-PCV era or to account for varying factors, such as temporal changes in the study population and vaccine utilisation [11]. Yet, evidence in this review and meta-estimates adds to the information base about serotypes’ individual importance in IPD and emphasise the need for closer attention to regional differences in the post-PCV era.

### Limitations

This study is not exempt from limitations of systematic reviews and meta-analysis. For some studies, case counts by serotype were not exact as we extracted data from images or calculated them from proportions. In other instances, serotype data were only available in a grouped format (e.g. “other”) or not all IPD cases were serotyped/reported. Missing information is likely to affect serotypes considered “rare” or “infrequent”, which will vary in each setting. These issues can potentially introduce biases to our results since not all serotypes could be assessed individually. Our meta-estimates could also be influenced by the differences across studies and are not exempt from risk of under or over-estimation. Although we sought to include large studies, datasets were heterogeneous, reported different sample sizes, observed diverse populations with varying rates of vaccine coverage, immunisation schedules, and methods for case detection of IPD (either by clinical and testing practices). Additionally, analyses were conducted individually per serotype, thus the sum of serotypes into categories may exceed 100%. Limitations withstanding, the large number of countries represented and isolates analysed is a strength of our review. We aimed to address issues of heterogeneity by analysing the most comparable data and case definitions. We also stratified analysis by PCV formulation and used data for children of the same age (<5 years) whenever possible. We would recommend future studies assessing serotype distribution after PCV introduction to report a clear definition and description of vaccine coverage in order contextualise results and better understand vaccine impact.

## Conclusion

In the post-PCV era, childhood IPD is associated with a wide number of serotypes. After PCV7 and in the early years after introduction of higher valent PCVs, 19A was the most commonly identified serotype in different world regions. Non-PCV13 serotypes caused a considerable proportion of childhood IPD, which emphasises the need for new vaccines with additional serotypes to reduce the remaining burden of childhood pneumococcal disease. Data on serotypes causing IPD from the regions with the highest burden were not available to draw robust regional conclusions. The geographic diversity of serotypes and changing epidemiology of *S*. *pneumoniae* underscores the importance of continued surveillance of pneumococcal serotypes to guide vaccine recommendations.

## Supporting information

S1 Checklist (DOC)

S1 Table Search strategies by database. (DOCX)

S2 Table Characteristics of studies excluded from meta-analysis. (DOCX)

S3 Table Proportional contribution of serotypes (%) to childhood IPD in individual studies included in meta-analysis. (DOCX)

S4 Table Additional serogroups/serotypes reported in studies identified through the review, not included in meta-analysis. (DOCX)

S5 Table Meta-analyses results. (DOCX)

S6 Table Sensitivity analysis. (DOCX)
