# Supplementary material for: Serotype distribution of Streptococcus pneumoniae causing invasive disease in children in the post-PCV era: A systematic review and meta-analysis
Source: PLoS One. 2017 May 9;12(5):e0177113. doi: 10.1371/journal.pone.0177113 (PMC5423631; doi:10.1371/journal.pone.0177113)
Supplement: S1 Table — (DOCX) [file pone.0177113.s002.docx]

# **Serotype distribution of Streptococcus pneumoniae causing invasive disease in young children during the post-PCV period**

## S1 Table. Search strategies by database

| **Medline In-Process & Other Non-Indexed Citations and Ovid MEDLINE(R)** | | |
| --- | --- | --- |
| 1 | exp Streptococcus pneumoniae/ | |
| 2 | Streptococcus pneumonia?.mp. | |
| 3 | "s? pneumonia?".mp. | |
| 4 | diplococcus pneumonia?.mp. | |
| **5** | 1 or 2 or 3 or 4 | |
| 6 | exp Pneumonia, Pneumococcal/ | |
| 7 | pneumonia.mp. | |
| 8 | exp Respiratory Tract Infections/ | |
| 9 | exp Pneumococcal Infections/ | |
| 10 | pneumococc*.mp. | |
| 11 | exp Meningitis, Pneumococcal/ | |
| 12 | meningitis.mp. | |
| 13 | exp Bacteremia/ | |
| 14 | bacter?emia.mp. | |
| 15 | invasive disease.mp. | |
| **16** | 6 or 7 or 8 or 9 or 10 or 11 or 12 or 13 or 14 or 15 | |
| 17 | exp Pneumococcal Vaccines/ | |
| 18 | PCV*.mp. | |
| 19 | exp Vaccines, Conjugate/ | |
| 20 | conjugate vaccine*.mp. | |
| 21 | polysaccharide vaccine*.mp. | |
| 22 | 7-valent.mp. | |
| 23 | hepta?valent.mp. | |
| 24 | seven-valent.mp. | |
| 25 | 10-valent.mp. | |
| 26 | deca?valent.mp. | |
| 27 | ten-valent.mp. | |
| 28 | 13-valent.mp. | |
| 29 | thirteen-valent.mp. | |
| 30 | 23-valent.mp. | |
| 31 | Prev?nar.mp. | |
| 32 | Pneumovax*.mp. | |
| **33** | 17 or 18 or 19 or 20 or 21 or 22 or 23 or 24 or 25 or 26 or 27 or 28 or 29 or 30 or 31 or 32 | |
| 34 | exp serogroup/ | |
| 35 | (Serotyp* or Serogroup*).mp. | |
| **36** | 34 or 35 | |
| **37** | 5 and 16 and 33 and 36 | |
| **38** | limit 37 to (humans and yr="2000 -Current" and "all child (0 to 18 years)") | |
| **Global Health Library** | | |
| [(streptococcus pneumonia or streptococcus pneumoniae) AND (invasive disease OR meningitis OR bacteremia OR bacteraemia OR pneumonia) AND (serotyp* OR serogroup*) AND (neonate* or neonatal or newborn* or new born* or infant* or infancy or baby or babies or toddler* or preschool or child* or juvenile* or girl* or boy* or young* or youth* or teenager* or adolescen* or pediatric* or paediatric*)](http://pesquisa.bvsalud.org/ghl/index.php) | | |
| **LILACS** | | |
| [(streptococcus pneumonia or streptococcus pneumoniae) AND (invasive disease OR meningitis OR bacteremia OR bacteraemia OR pneumonia) AND (serotyp* OR serogroup*) AND (neonate* or neonatal or newborn* or new born* or infant* or infancy or baby or babies or toddler* or preschool or child* or juvenile* or girl* or boy* or young* or youth* or teenager* or adolescen* or pediatric* or paediatric*)](http://pesquisa.bvsalud.org/ghl/index.php) | | |
| **Web** **of Science** | | |
| **TOPIC:**(streptococcus pneumonia?) AND (invasive disease OR meningitis OR bacter?emia OR pneumonia) AND **TOPIC:** (serotyp* OR serogroup*) AND **TOPIC:** (neonate* or neonatal or newborn* or new born* or infant* or infancy or baby or babies or toddler* or preschool or child* or juvenile* or girl* or boy* or young* or youth* or teenager* or adolescen* or pediatric* or paediatric*) **Timespan:** 2000-2015. **Indexes:** SCI-EXPANDED, CPCI-S. | | |
| **Embase - 1980 to 2015** | | |
| 1 | | exp Streptococcus pneumoniae/ |
| 2 | | Streptococcus pneumonia?.mp. |
| 3 | | "s? pneumonia?".mp. |
| 4 | | diplococcus pneumonia?.mp. |
| **5** | | 1 or 2 or 3 or 4 |
| 6 | | exp Pneumonia, Pneumococcal/ |
| 7 | | pneumonia.mp. |
| 8 | | exp lower respiratory tract infection/ |
| 9 | | exp Pneumococcal Infections/ |
| 10 | | pneumococc*.mp. |
| 11 | | exp Meningitis, Pneumococcal/ |
| 12 | | meningitis.mp. |
| 13 | | exp Bacteremia/ |
| 14 | | bacter?emia.mp. |
| 15 | | invasive disease.mp. |
| 16 | | 6 or 7 or 8 or 9 or 10 or 11 or 12 or 13 or 14 or 15 |
| 17 | | exp Pneumococcal Vaccines/ |
| 18 | | PCV*.mp. |
| 19 | | exp Vaccines, Conjugate/ |
| 20 | | conjugate vaccine*.mp. |
| 21 | | polysaccharide vaccine*.mp. |
| 22 | | 7-valent.mp. |
| 23 | | seven-valent.mp. |
| 24 | | hepta-valent.mp. |
| 25 | | 10-valent.mp. |
| 26 | | ten-valent.mp. |
| 27 | | decavalent.mp. |
| 28 | | 13-valent.mp. |
| 29 | | 23-valent.mp. |
| 30 | | Prev?nar.mp. |
| 31 | | Pneumovax*.mp. |
| 32 | | 17 or 18 or 19 or 20 or 21 or 22 or 23 or 24 or 25 or 26 or 27 or 28 or 29 or 30 or 31 |
| 33 | | exp serotype/ |
| 34 | | (Serotyp* or Serogroup*).mp. |
| 35 | | 33 or 34 |
| 36 | | 5 and 16 and 32 and 35 |
| 37 | | limit 36 to (yr="2000 -Current" and (infant <to one year> or child <unspecified age> or preschool child <1 to 6 years> or school child <7 to 12 years> or adolescent <13 to 17 years>)) |
| **Global Health** | | |
| 1 | | exp Streptococcus pneumoniae/ |
| 2 | | streptococcus pneumonia?.mp. |
| 3 | | "s? pneumonia?".mp. |
| 4 | | diplococcus pneumonia?.mp. |
| **5** | | 1 or 2 or 3 or 4 |
| 6 | | exp lower respiratory tract infections/ |
| 7 | | pneumonia.mp. |
| 8 | | Pneumococc*.mp. |
| 9 | | meningitis.mp. |
| 10 | | bacter?emia.mp. |
| 11 | | invasive disease.mp. |
| 12 | | 6 or 7 or 8 or 9 or 10 or 11 |
| 13 | | PCV*.mp. |
| 14 | | conjugate vaccine*.mp. |
| 15 | | polysaccharide vaccine*.mp. |
| 16 | | 7-valent conjugate vaccine.mp. |
| 17 | | 10-valent conjugate vaccine.mp. |
| 18 | | 13-valent conjugate vaccine.mp. |
| 19 | | 23-valent pneumococcal polysaccharide vaccine.mp. |
| 20 | | prev?nar.mp. |
| 21 | | pneumovax*.mp. |
| 22 | | 13 or 14 or 15 or 16 or 17 or 18 or 19 or 20 or 21 |
| 23 | | exp serotype/ |
| 24 | | (serotyp* or serogroup*).mp. |
| 25 | | 23 or 24 |
| 26 | | 5 and 12 and 22 and 25 |
| 27 | | limit 26 to yr="2000 -Current" |
| 28 | | (neonate* or neonatal or newborn* or new born* or infant* or infancy or baby or babies or toddler* or preschool or child* or juvenile* or girl* or boy* or young* or youth* or teenager* or adolescen* or pediatric* or paediatric*).mp. |
| 29 | | 27 and 28 |
