# Supplementary material for: Serotype distribution of Streptococcus pneumoniae causing invasive disease in children in the post-PCV era: A systematic review and meta-analysis
Source: PLoS One. 2017 May 9;12(5):e0177113. doi: 10.1371/journal.pone.0177113 (PMC5423631; doi:10.1371/journal.pone.0177113)
Supplement: S2 Table — (DOCX) [file pone.0177113.s003.docx]

# **Serotype distribution of Streptococcus pneumoniae causing invasive disease in children during the post-PCV period**

Evelyn Balsells, Laurence Guillot, Harish Nair, Moe H. Kyaw

## S2 Table. Characteristics of studies excluded from meta-analysis

| Reference | Region | Country | Outcome | Year of PCV introduction | Study period | PCV implemented | Age | Remarks | Isolates | PCV13 types (%) | Non-PCV13 (%) |
| --- | --- | --- | --- | --- | --- | --- | --- | --- | --- | --- | --- |
| Foster (2011) | Europe | UK | IPD | 2006 | 2006-09 | PCV7 | <2y | Overlap with another study included in meta-analysis | 53 | 56.6 | 43.4 |
| Alexandre (2010) | Europe | France | MENG | 2002 | 2005-08 | PCV7 | <18y | IPD data from this country available | 35 | 65.7 | 34.3 |
| Doit (2010) | Europe | France | BACT | 2002  (Recommended) | 2005-08 | PCV7 | ≤15y | IPD data from this country available | 55 | 80.0 | 20.0 |
| Herrero (2012) | Europe | Spain | BACT | 2001 | 2008-09 | PCV7 | <14y | IPD data from this country available | 20 | 85.0 | 15.0 |
| Mistry (2013) | North America | USA | BACT | 2000 | 2003-09 | PCV7 | ≤18y | IPD data from this country available | 89 | 57.3 | 42.7 |
| Herz (2006) | North America | USA | BACT | 2000 | 2001-03 | PCV7 | 3-36m | IPD data from this country available | 25 | 36.0 | 64.0 |
| Hsu (2005) | North America | USA | IPD | 2000 | 2001-03 | PCV7 | <18y | Partial overlap with more recent publication included in meta-analysis | 136 | 64.0 | 36.0 |
| Hicks (2007) | North America | USA | IPD | 2000 | 2004 | PCV7 | <5y | Overlap with another study included in meta-analysis | 297 | 59.3 | 40.7 |
| Kaplan (2009) | North America | USA | IPD | 2000 | 2003-08 | PCV7 | Children | More recent publication included in meta-analysis | 1029 | 68.2 | 31.8 |
| Olarte (2015) | North America | USA | MENG | 2000 | 2007-09 | PCV7 | ≤18y | IPD data from this country available | 74 | 54.1 | 45.9 |
| Lepoutre (2008) | Europe | France | IPD | 2003 (high risk),  2006 June (Universal) | 2006 | PCV7 | <2y | Year of PCV7 introduction, most recent publication from this setting is available | 166 | 70.5 | 29.5 |
| Levy (2011) | Europe | France | MENG | 2006 | 2007-08 | PCV7 | <5y | IPD data from this country available | 187 | 59.4 | 40.6 |
| Imohl (2015) | Europe | Germany | MENG | 2006 | 2007-10 | PCV7 | <16y | IPD data from this country available | 227 | 60.4 | 39.6 |
| Desai (2015) | North America | USA | IPD | 2010 March | 2010-13 | PCV13 | 6-59m | Overlap with another study included in meta-analysis | 98 | 23.5 | 76.5 |
| Olarte (2015) | North America | USA | MENG | 2010 | 2010-13 | PCV13 | ≤18y | IPD data from this country available | 92 | 31.5 | 68.5 |
| Angoulvant (2014) | Europe | France | PNEUM | 2009 | 2009-12 | PCV7/13 | 1m-<15 | IPD data from this country available | 62 | 87.1 | 12.9 |
| Levy (2014) | Europe | France | MENG | 2010 July | 2010-12 | PCV13 | ≤15y | IPD data from this country available | 238 | 39.5 | 60.5 |
| Imohl (2015) | Europe | Germany | MENG | 2006 | 2010/11-12/13 | PCV13 | <16y | IPD data from this country available | 194 | 29.4 | 70.6 |
| del Amo (2014) | Europe | Spain | IPD | 2001/2009/2010 | 2007-11 | PCV7/10/13 | <6y | Not possible to disaggregate data for a particular PCV | 159 | 84.9 | 15.1 |
| del Amo (2015) | Europe | Spain | IPD | 2001 Licensed | 2007-11 | PCV7/10 | <6yy | Not possible to disaggregate data for a particular PCV | 358 | 56.1 | 43.9 |
