# Supplementary material for: Serotype distribution of Streptococcus pneumoniae causing invasive disease in children in the post-PCV era: A systematic review and meta-analysis
Source: PLoS One. 2017 May 9;12(5):e0177113. doi: 10.1371/journal.pone.0177113 (PMC5423631; doi:10.1371/journal.pone.0177113)
Supplement: S3 Table — (DOCX) [file pone.0177113.s004.docx]

# **Serotype distribution of Streptococcus pneumoniae causing invasive disease in young children during the post-PCV period**

Evelyn Balsells, Laurence Guillot, Harish Nair, Moe H. Kyaw

## S3 Table. Proportional contribution of serotypes (%) to childhood IPD in individual studies included in meta-analysis

| Last author (Ref) | Isolates | PCV13 types | Non-PCV13 | 3 | 6A | 19A | 22F | 24F | 33F | 12F | 15B | 10A | 8 | 38 | 15A | 23B | 15C | 6C | 35B | 11A | 23A | 9N |
| --- | --- | --- | --- | --- | --- | --- | --- | --- | --- | --- | --- | --- | --- | --- | --- | --- | --- | --- | --- | --- | --- | --- |
| Rendi-Wagner (2009) | 36 | 97.2 | 2.8 | 2.8 | 8.3 | 0.0 | 0.0 | 0.0 | 0.0 | 0.0 | 0.0 | 0.0 | 0.0 | 0.0 | 0.0 | 0.0 | 0.0 | 0.0 | 0.0 | 0.0 | 2.8 | 0.0 |
| Lepoutre (2015) | 483 | 71.4 | 28.6 | 3.9 | 1.4 | 27.5 | 1.9 | 4.6 | 0.0 | 1.9 | 1.9 | 0.0 | 0.0 | 0.0 | 1.9 | 0.0 | 1.2 | 1.2 | 0.0 | 0.0 | 0.0 | 0.0 |
| Varon (2015) | 388 | 67.5 | 32.5 | 4.6 | 0.8 | 33.0 | 1.5 | 6.4 | 1.3 | 2.3 | 0.0 | 1.8 | 0.3 | 0.0 | 1.5 | 0.8 | 0.0 | 1.0 | 0.3 | 0.3 | 0.5 | 0.3 |
| van der Linden (2015) | 542 | 68.1 | 31.9 | 6.1 | 4.2 | 9.2 | 1.8 | 3.9 | 1.3 | 1.3 | 2.8 | 4.8 | 0.9 | 2.8 | 0.4 | 0.6 | 3.0 | 0.6 | 0.2 | 0.6 | 0.6 | 1.1 |
| Knol (2015) | 128 | 52.3 | 47.7 | 3.1 | 1.6 | 22.7 | 3.9 | 2.3 | 8.6 | 1.6 | 2.3 | 10.9 | 2.3 | 0.8 | 0.8 | 0.8 | 0.8 | 0.0 | 0.0 | 2.3 | 0.8 | 0.0 |
| Steens (2013) | 165* | 69.1 | 30.9 | 6.7 | 5.5 | 10.3 | 5.5 | 0.0 | 5.5 | 0.0 | 0.0 | 2.4 | 0.0 | 1.2 | 0.0 | 1.2 | 0.0 | 1.2 | 0.0 | 0.0 | 0.0 | 0.0 |
| Aguiar (2008) | 90 | 85.6 | 14.4 | 3.3 | 2.2 | 24.4 | 2.2 | 1.1 | 1.1 | 0.0 | 1.1 | 2.2 | 1.1 | 0.0 | 0.0 | 1.1 | 0.0 | 0.0 | 0.0 | 0.0 | 0.0 | 0.0 |
| Aristegui (2007) | 77 | 88.3 | 11.7 | 2.6 | 0.0 | 0.0 | 0.0 | 0.0 | 0.0 | 0.0 | 0.0 | 0.0 | 0.0 | 0.0 | 0.0 | 0.0 | 0.0 | 0.0 | 0.0 | 0.0 | 0.0 | 0.0 |
| Barricarte (2007) | 85 | 87.1 | 12.9 | 5.9 | 7.1 | 22.4 | 0.0 | 0.0 | 0.0 | 0.0 | 1.2 | 0.0 | 0.0 | 0.0 | 0.0 | 2.4 | 2.4 | 0.0 | 0.0 | 0.0 | 0.0 | 0.0 |
| Calbo (2006) | 64 | 78.1 | 21.9 | 0.0 | 9.4 | 0.0 | 0.0 | 0.0 | 0.0 | 0.0 | 0.0 | 0.0 | 0.0 | 3.1 | 0.0 | 0.0 | 1.6 | 0.0 | 0.0 | 0.0 | 0.0 | 0.0 |
| Guevara (2014) | 106 | 78.3 | 21.7 | 4.7 | 1.9 | 32.1 | 1.9 | 3.8 | 1.9 | 0.9 | 0.9 | 1.9 | 0.0 | 0.0 | 0.9 | 0.0 | 0.0 | 1.9 | 0.0 | 0.0 | 0.0 | 0.0 |
| Munoz-Almagro (2011) | 130 | 80.0 | 20.0 | 3.1 | 2.3 | 20.0 | 0.8 | 4.6 | 0.0 | 0.8 | 0.8 | 2.3 | 0.0 | 2.3 | 0.0 | 2.3 | 0.0 | 0.0 | 0.0 | 0.0 | 0.0 | 1.5 |
| Perez-Trallero (2009) | 45 | 86.7 | 13.3 | 0.0 | 4.4 | 13.3 | 0.0 | 0.0 | 0.0 | 0.0 | 0.0 | 0.0 | 0.0 | 0.0 | 0.0 | 0.0 | 0.0 | 0.0 | 0.0 | 0.0 | 0.0 | 0.0 |
| Picazo (2011) | 330 | 79.1 | 20.9 | 3.9 | 1.2 | 18.8 | 0.6 | 1.5 | 0.3 | 1.5 | 1.8 | 0.9 | 0.6 | 0.0 | 0.6 | 0.9 | 0.6 | 0.6 | 0.9 | 1.2 | 0.0 | 0.0 |
| Rodriguez (2011) | 366 | 74.9 | 25.1 | 3.3 | 2.2 | 22.4 | 1.1 | 1.9 | 0.0 | 2.2 | 3.3 | 1.9 | 0.8 | 0.0 | 0.8 | 2.2 | 1.1 | 0.0 | 0.8 | 0.8 | 0.5 | 0.0 |
| Salleras (2009) | 240 | 77.9 | 22.1 | 2.1 | 7.1 | 22.1 | 0.4 | 4.6 | 2.1 | 2.9 | 0.0 | 2.5 | 0.4 | 0.8 | 0.4 | 2.5 | 0.0 | 0.0 | 0.8 | 0.0 | 0.0 | 0.8 |
| Vila-Corcoles (2013) | 65 | 84.6 | 15.4 | 0.0 | 0.0 | 10.8 | 1.5 | 0.0 | 0.0 | 3.1 | 0.0 | 1.5 | 0.0 | 0.0 | 0.0 | 0.0 | 3.1 | 0.0 | 1.5 | 1.5 | 0.0 | 1.5 |
| Ceyhan (2011) | 146 | 77.4 | 22.6 | 4.1 | 2.1 | 6.2 | 0.0 | 0.0 | 0.0 | 0.0 | 0.0 | 0.0 | 1.4 | 0.0 | 0.0 | 0.0 | 1.4 | 0.0 | 0.0 | 0.0 | 0.7 | 0.0 |
| Miller (2011) | 528 | 69.7 | 30.3 | 5.7 | 0.0 | 18.9 | 5.7 | 0.6 | 3.6 | 1.5 | 2.5 | 1.1 | 2.8 | 1.1 | 0.9 | 1.3 | 2.5 | 0.0 | 0.6 | 1.5 | 0.8 | 0.6 |
| Moore (2014) | 65 | 49.2 | 50.8 | 0.0 | 1.5 | 13.8 | 4.6 | 0.0 | 13.8 | 3.1 | 1.5 | 0.0 | 1.5 | 1.5 | 1.5 | 1.5 | 6.2 | 3.1 | 0.0 | 0.0 | 0.0 | 3.1 |
| Parra (2013) | 84 | 70.2 | 29.8 | 14.3 | 1.2 | 6.0 | 1.2 | 1.2 | 0.0 | 0.0 | 3.6 | 0.0 | 2.4 | 0.0 | 2.4 | 1.2 | 2.4 | 0.0 | 2.4 | 2.4 | 0.0 | 0.0 |
| Bettinger (2010) | 212 | 69.8 | 30.2 | 8.0 | 4.2 | 20.3 | 6.1 | 0.0 | 1.9 | 0.0 | 2.8 | 1.9 | 0.0 | 2.8 | 0.9 | 1.4 | 1.4 | 0.0 | 0.9 | 1.4 | 0.0 | 0.5 |
| De Wals (2012) | 113 | 63.7 | 36.3 | 4.4 | 2.7 | 43.4 | 4.4 | 0.0 | 3.5 | 0.0 | 1.8 | 2.7 | 0.0 | 1.8 | 2.7 | 0.9 | 2.7 | 1.8 | 0.0 | 0.9 | 1.8 | 0.0 |
| Kellner (2009) | 67 | 76.1 | 23.9 | 9.0 | 3.0 | 4.5 | 6.0 | 0.0 | 0.0 | 0.0 | 0.0 | 0.0 | 1.5 | 1.5 | 0.0 | 0.0 | 0.0 | 0.0 | 0.0 | 0.0 | 0.0 | 0.0 |
| Black (2007) | 84 | 34.5 | 65.5 | 2.4 | 3.6 | 11.9 | 9.5 | 0.0 | 4.8 | 1.2 | 1.2 | 3.6 | 1.2 | 8.3 | 0.0 | 1.2 | 7.1 | 0.0 | 1.2 | 2.4 | 0.0 | 0.0 |
| Bruce (2015) | 126 | 61.9 | 38.1 | 5.6 | 2.4 | 31.7 | 4.0 | 0.0 | 3.2 | 4.8 | 0.8 | 1.6 | 0.8 | 0.0 | 2.4 | 4.0 | 0.8 | 1.6 | 1.6 | 0.8 | 0.0 | 1.6 |
| Byington (2005) | 105 | 74.3 | 25.7 | 9.5 | 0.0 | 0.0 | 0.0 | 0.0 | 0.0 | 0.0 | 0.0 | 0.0 | 1.9 | 0.0 | 0.0 | 0.0 | 0.0 | 0.0 | 0.0 | 0.0 | 0.0 | 0.0 |
| Croney (2013) | 157 | 60.5 | 39.5 | 3.8 | 0.6 | 32.5 | 0.0 | 0.0 | 0.0 | 0.0 | 0.0 | 0.0 | 0.0 | 0.0 | 0.0 | 1.9 | 0.0 | 4.5 | 3.2 | 0.0 | 2.5 | 0.6 |
| Hsu (2010) | 130 | 66.2 | 33.8 | 4.6 | 12.3 | 23.8 | 6.2 | 0.0 | 5.4 | 1.5 | 0.0 | 1.5 | 0.0 | 0.0 | 0.0 | 0.0 | 0.0 | 0.0 | 0.0 | 0.0 | 0.0 | 0.0 |
| Kaplan (2013) | 609 | 67.7 | 32.3 | 7.7 | 0.3 | 40.1 | 3.4 | 0.0 | 0.0 | 0.0 | 2.6 | 0.0 | 0.5 | 1.0 | 1.0 | 2.3 | 2.0 | 3.6 | 0.0 | 0.0 | 1.6 | 0.7 |
| Pilishvili (2010) | 519 | 67.9 | 32.1 | 5.0 | 0.0 | 47.2 | 4.8 | 0.0 | 4.8 | 1.5 | 0.0 | 2.7 | 0.0 | 2.7 | 1.5 | 1.4 | 0.0 | 0.0 | 0.6 | 0.8 | 1.7 | 0.6 |
| Schutze (2004) | 75 | 77.3 | 22.7 | 1.3 | 0.0 | 0.0 | 0.0 | 0.0 | 0.0 | 0.0 | 0.0 | 0.0 | 0.0 | 0.0 | 0.0 | 0.0 | 0.0 | 0.0 | 0.0 | 0.0 | 0.0 | 0.0 |
| Sharma (2013)* | 47 | 59.6 | 40.4 | 2.1 | 0.0 | 48.9 | 4.3 | 0.0 | 12.8 | 2.1 | 0.0 | 0.0 | 0.0 | 4.3 | 0.0 | 2.1 | 0.0 | 0.0 | 2.1 | 0.0 | 0.0 | 0.0 |
| Weatherholtz (2010) | 115 | 76.5 | 23.5 | 8.7 | 4.3 | 19.1 | 3.5 | 0.0 | 0.0 | 9.6 | 0.0 | 2.6 | 0.9 | 2.6 | 0.0 | 1.7 | 0.0 | 0.0 | 0.0 | 0.0 | 0.0 | 0.0 |
| Williams (2011) | 201 | 67.2 | 32.8 | 4.5 | 4.5 | 36.8 | 4.5 | 0.0 | 1.5 | 0.0 | 0.0 | 0.0 | 0.0 | 0.0 | 0.0 | 0.0 | 0.0 | 0.0 | 0.0 | 3.0 | 0.0 | 0.0 |
| Chiba (2014) | 302 | 55.0 | 45.0 | 2.0 | 2.0 | 15.9 | 4.6 | 0.0 | 2.0 | 0.0 | 3.3 | 1.0 | 0.0 | 2.0 | 6.6 | 0.0 | 5.0 | 5.6 | 2.0 | 0.0 | 3.6 | 0.0 |
| Ishiwada (2014) | 33 | 51.5 | 48.5 | 3.0 | 3.0 | 27.3 | 6.1 | 6.1 | 6.1 | 0.0 | 0.0 | 6.1 | 0.0 | 3.0 | 15.2 | 0.0 | 3.0 | 0.0 | 3.0 | 0.0 | 0.0 | 0.0 |
| Suga (2015) | 308 | 68.8 | 31.2 | 1.3 | 2.6 | 27.9 | 2.3 | 4.9 | 1.9 | 0.3 | 2.3 | 2.9 | 0.0 | 1.3 | 5.8 | 0.0 | 4.5 | 2.6 | 0.6 | 0.6 | 0.3 | 0.0 |
| von Gottberg (2013) | 839 | 57.3 | 42.7 | 2.0 | 8.5 | 11.4 | 0.0 | 0.0 | 0.0 | 4.0 | 4.3 | 0.0 | 8.3 | 0.0 | 0.0 | 0.0 | 0.0 | 0.0 | 0.0 | 0.0 | 0.0 | 1.8 |
| Al-Sheikh (2014) | 78 | 85.9 | 14.1 | 2.6 | 2.6 | 3.8 | 0.0 | 0.0 | 0.0 | 1.3 | 0.0 | 0.0 | 1.3 | 0.0 | 0.0 | 1.3 | 0.0 | 0.0 | 0.0 | 0.0 | 2.6 | 0.0 |
| Shibl (2012) | 108 | 94.4 | 5.6 | 1.9 | 6.5 | 7.4 | 0.0 | 0.0 | 0.0 | 0.0 | 0.0 | 0.0 | 0.0 | 0.0 | 0.0 | 0.0 | 0.0 | 0.0 | 0.0 | 0.0 | 0.0 | 0.0 |
| Varon (2015) | 181 | 17.7 | 82.3 | 2.8 | 0.6 | 8.8 | 5.0 | 19.9 | 3.3 | 8.8 | 0.0 | 7.7 | 0.0 | 2.8 | 7.2 | 1.1 | 0.0 | 0.6 | 2.2 | 1.1 | 2.2 | 0.0 |
| van der Linden (2015) | 567 | 35.4 | 64.6 | 5.3 | 1.1 | 11.8 | 3.5 | 8.8 | 3.2 | 4.1 | 3.9 | 6.9 | 1.2 | 6.5 | 1.2 | 4.8 | 6.0 | 1.1 | 1.2 | 1.9 | 1.4 | 1.4 |
| Knol (2015) | 57 | 21.1 | 78.9 | 0.0 | 0.0 | 19.3 | 5.3 | 1.8 | 10.5 | 3.5 | 1.8 | 28.1 | 3.5 | 1.8 | 0.0 | 0.0 | 7.0 | 1.8 | 1.8 | 0.0 | 1.8 | 1.8 |
| Steens (2013) | 47 | 55.3 | 44.7 | 6.4 | 0.0 | 21.3 | 4.3 | 2.1 | 4.3 | 0.0 | 0.0 | 4.3 | 0.0 | 0.0 | 0.0 | 0.0 | 0.0 | 8.5 | 0.0 | 2.1 | 0.0 | 0.0 |
| Guevara (2014) | 25 | 52.0 | 48.0 | 12.0 | 0.0 | 24.0 | 4.0 | 4.0 | 4.0 | 8.0 | 0.0 | 0.0 | 0.0 | 0.0 | 0.0 | 0.0 | 0.0 | 4.0 | 0.0 | 0.0 | 0.0 | 0.0 |
| Moore (2014) | 48 | 37.5 | 62.5 | 4.2 | 0.0 | 16.7 | 0.0 | 2.1 | 6.3 | 6.3 | 4.2 | 8.3 | 6.3 | 2.1 | 0.0 | 2.1 | 10.4 | 2.1 | 0.0 | 2.1 | 0.0 | 0.0 |
| Scotland Surv | 206 | 19.9 | 80.1 | 3.9 | 0.0 | 5.8 | 4.4 | 0.5 | 2.9 | 4.9 | 3.4 | 2.9 | 1.0 | 1.0 | 2.9 | 2.4 | 2.4 | 0.5 | 1.5 | 1.0 | 0.0 | 0.0 |
| Waight (2015) | 247 | 14.2 | 85.8 | 3.2 | 0.0 | 2.8 | 9.3 | 11.3 | 8.1 | 6.1 | 0.0 | 2.8 | 6.1 | 3.2 | 7.3 | 7.3 | 0.0 | 0.8 | 0.8 | 1.6 | 1.2 | 0.4 |
| SIREVA (Brasil) | 416 | 68.8 | 31.3 | 10.6 | 5.5 | 8.4 | 1.9 | 1.0 | 0.0 | 3.4 | 0.2 | 1.0 | 1.0 | 0.0 | 0.7 | 0.5 | 1.7 | 2.9 | 0.2 | 1.9 | 0.5 | 1.2 |
| SIREVA (Chile) | 168 | 72.6 | 27.4 | 4.8 | 7.1 | 8.3 | 3.0 | 3.6 | 2.4 | 3.6 | 1.2 | 0.6 | 0.6 | 1.8 | 0.6 | 0.0 | 1.2 | 2.4 | 0.0 | 1.2 | 0.0 | 0.0 |
| SIREVA (Colombia) | 208 | 74.0 | 26.0 | 5.8 | 4.3 | 10.1 | 0.0 | 1.0 | 0.0 | 0.0 | 2.9 | 1.0 | 1.4 | 0.0 | 1.0 | 1.4 | 0.0 | 1.4 | 0.5 | 2.9 | 2.9 | 0.0 |
| SIREVA (Costa Rica) | 38 | 76.3 | 23.7 | 5.3 | 0.0 | 18.4 | 0.0 | 0.0 | 0.0 | 0.0 | 0.0 | 2.6 | 0.0 | 0.0 | 5.3 | 0.0 | 0.0 | 0.0 | 0.0 | 5.3 | 0.0 | 2.6 |
| SIREVA (Ecuador) | 62 | 80.6 | 19.4 | 3.2 | 6.5 | 16.1 | 0.0 | 4.8 | 0.0 | 0.0 | 0.0 | 1.6 | 0.0 | 0.0 | 1.6 | 3.2 | 0.0 | 1.6 | 0.0 | 1.6 | 0.0 | 0.0 |
| SIREVA (El Salvador) | 31 | 74.2 | 25.8 | 0.0 | 0.0 | 16.1 | 0.0 | 0.0 | 0.0 | 0.0 | 0.0 | 0.0 | 3.2 | 0.0 | 0.0 | 0.0 | 0.0 | 0.0 | 0.0 | 0.0 | 6.5 | 0.0 |
| SIREVA (Mexico) | 105 | 66.7 | 33.3 | 3.8 | 1.9 | 39.0 | 1.0 | 0.0 | 0.0 | 1.0 | 4.8 | 2.9 | 0.0 | 0.0 | 0.0 | 4.8 | 1.0 | 1.9 | 6.7 | 1.9 | 1.0 | 0.0 |
| SIREVA (Panama) | 68 | 88.2 | 11.8 | 1.5 | 5.9 | 8.8 | 0.0 | 2.9 | 0.0 | 0.0 | 0.0 | 0.0 | 0.0 | 0.0 | 2.9 | 0.0 | 1.5 | 0.0 | 0.0 | 1.5 | 0.0 | 0.0 |
| SIREVA (Peru) | 23 | 69.6 | 30.4 | 8.7 | 4.3 | 17.4 | 0.0 | 0.0 | 0.0 | 4.3 | 0.0 | 0.0 | 0.0 | 0.0 | 0.0 | 4.3 | 0.0 | 4.3 | 0.0 | 0.0 | 0.0 | 0.0 |
| SIREVA (Uruguay) | 96 | 50.0 | 50.0 | 12.5 | 0.0 | 3.1 | 3.1 | 3.1 | 1.0 | 7.3 | 0.0 | 1.0 | 0.0 | 0.0 | 1.0 | 0.0 | 0.0 | 0.0 | 0.0 | 2.1 | 1.0 | 1.0 |
| Demczuk (2013) | 886 | 55.5 | 44.5 | 8.5 | 1.1 | 32.8 | 6.1 | 0.0 | 1.9 | 1.5 | 2.7 | 2.5 | 1.7 | 2.8 | 3.7 | 2.6 | 2.9 | 1.9 | 2.1 | 2.5 | 2.1 | 0.7 |
| Bruce (2015) | 52 | 25.0 | 75.0 | 1.9 | 0.0 | 15.4 | 7.7 | 0.0 | 5.8 | 7.7 | 7.7 | 3.8 | 0.0 | 0.0 | 7.7 | 1.9 | 5.8 | 0.0 | 5.8 | 1.9 | 1.9 | 0.0 |
| Kaplan (2013) | 283 | 56.5 | 43.5 | 4.6 | 0.4 | 33.2 | 4.2 | 0.0 | 5.7 | 0.0 | 2.5 | 0.0 | 0.4 | 0.0 | 1.1 | 2.1 | 2.5 | 3.5 | 0.0 | 0.0 | 2.5 | 1.1 |
| Moore (2015) | 177 | 19.2 | 80.8 | 5.6 | 0.0 | 7.3 | 11.3 | 0.0 | 9.6 | 4.0 | 7.3 | 0.0 | 0.0 | 9.0 | 0.0 | 5.1 | 6.8 | 1.7 | 7.9 | 0.0 | 0.0 | 0.0 |
| Australia Surv | 184 | 43.5 | 56.5 | 6.0 | 0.5 | 22.8 | 6.0 | 0.5 | 4.9 | 0.0 | 2.2 | 2.2 | 0.0 | 0.5 | 0.5 | 4.3 | 4.3 | 2.2 | 4.3 | 0.5 | 0.5 | 0.5 |
| Nakano (2015) | 126 | 28.6 | 71.4 | 0.8 | 0.0 | 24.6 | 11.1 | 22.2 | 0.8 | 0.0 | 0.0 | 4.0 | 0.0 | 0.8 | 11.1 | 0.0 | 0.0 | 1.6 | 3.2 | 0.8 | 2.4 | 0.0 |
| New Zealand Surv | 78 | 51.3 | 48.7 | 14.1 | 0.0 | 26.9 | 3.8 | 0.0 | 5.1 | 0.0 | 5.1 | 0.0 | 3.8 | 0.0 | 0.0 | 2.6 | 0.0 | 7.7 | 0.0 | 0.0 | 3.8 | 2.6 |
| Singapore Surv | 65 | 86.2 | 13.8 | 9.2 | 0.0 | 50.8 | 0.0 | 0.0 | 0.0 | 0.0 | 0.0 | 0.0 | 0.0 | 0.0 | 0.0 | 0.0 | 0.0 | 1.5 | 0.0 | 0.0 | 0.0 | 0.0 |
