# Supplementary material for: Serotype distribution of Streptococcus pneumoniae causing invasive disease in children in the post-PCV era: A systematic review and meta-analysis
Source: PLoS One. 2017 May 9;12(5):e0177113. doi: 10.1371/journal.pone.0177113 (PMC5423631; doi:10.1371/journal.pone.0177113)
Supplement: S4 Table — (DOCX) [file pone.0177113.s005.docx]

# **Serotype distribution of Streptococcus pneumoniae causing invasive disease in young children during the post-PCV period**

Evelyn Balsells, Laurence Guillot, Harish Nair, Moe H. Kyaw

## S4 Table. Additional serogroups/serotypes reported in studies identified through the review, not included in meta-analysis

| Serotype | Number of studies | Number of IPD cases | Serotype | Number of studies | | Number of PD cases | |
| --- | --- | --- | --- | --- | --- | --- | --- |
| 2 | 2 | 3 | **18A** | 6 | 12 | |  |
| 10 | 9 | 30 | **18B** | 5 | 12 | |  |
| 11 | 8 | 23 | **18F** | 2 | 2 | |  |
| 12 | 9 | 34 | **19C** | 1 | 1 | |  |
| 13 | 9 | 24 | **19D** | 1 | 3 | |  |
| 15 | 11 | 74 | **22A** | 1 | 1 | |  |
| 16 | 8 | 13 | **22F/A** | 2 | 9 | |  |
| 17 | 7 | 9 | **23AB** | 2 | 3 | |  |
| 20 | 16 | 24 | **24A** | 2 | 6 | |  |
| 21 | 20 | 58 | **24B** | 10 | 16 | |  |
| 22 | 6 | 16 | **24C** | 1 | 1 | |  |
| 24 | 9 | 30 | **25A** | 10 | 41 | |  |
| 27 | 8 | 13 | **28A** | 5 | 7 | |  |
| 28 | 5 | 6 | **28F** | 3 | 7 | |  |
| 29 | 7 | 7 | **28FA** | 1 | 1 | |  |
| 31 | 10 | 16 | **33A** | 6 | 26 | |  |
| 33 | 6 | 14 | **33B** | 5 | 11 | |  |
| 34 | 16 | 35 | **33FA** | 1 | 2 | |  |
| 35 | 6 | 16 | **33FA37** | 1 | 1 | |  |
| 37 | 3 | 5 | **35A** | 2 | 2 | |  |
| 39 | 1 | 1 | **35BF** | 1 | 2 | |  |
| 45 | 1 | 1 | **35F** | 23 | 57 | |  |
| 46 | 3 | 4 | **35F47F** | 1 | 1 | |  |
| 10A39 | 1 | 2 | **38/25F** | 1 | 1 | |  |
| 10B | 3 | 3 | **41F** | 1 | 1 | |  |
| 10F | 1 | 1 | **7A** | 1 | 1 | |  |
| 11AD | 1 | 1 | **7B** | 1 | 1 | |  |
| 11ADF | 1 | 1 | **7C** | 13 | 33 | |  |
| 11B | 1 | 1 | **9A** | 3 | 4 | |  |
| 11E | 1 | 1 | **9L** | 2 | 2 | |  |
| 11F | 2 | 3 | **poolC** | 2 | 2 | |  |
| 12A | 1 | 2 | **PoolD+** | 1 | 1 | |  |
| 12B | 3 | 3 | **PoolG** | 2 | 28 | |  |
| 12FB | 1 | 6 | **PoolH+** | 1 | 1 | |  |
| 15AF | 2 | 4 |  |  |  |  |  |
| 15CF | 1 | 3 |  |  |  |  |  |
| 15F | 5 | 6 |  |  |  |  |  |
| 16F | 38 | 84 |  |  |  |  |  |
| 17F | 22 | 50 |  |  |  |  |  |
| 17FA | 1 | 4 |  |  |  |  |  |
