# Supplementary material for: Serotype distribution of Streptococcus pneumoniae causing invasive disease in children in the post-PCV era: A systematic review and meta-analysis
Source: PLoS One. 2017 May 9;12(5):e0177113. doi: 10.1371/journal.pone.0177113 (PMC5423631; doi:10.1371/journal.pone.0177113)
Supplement: S5 Table — (DOCX) [file pone.0177113.s006.docx]

# **Serotype distribution of Streptococcus pneumoniae causing invasive disease in young children during the post-PCV period**

Evelyn Balsells, Laurence Guillot, Harish Nair, Moe H. Kyaw

## S5 Table. Meta-analyses results

(PCV7 implementation)

Study | ES [95% Conf. Interval] % Weight

---------------------+---------------------------------------------------

non-pcv

Aguiar (2008) | 14.444 8.387 24.876 0.20

Aristegui (2007) | 11.688 6.082 22.464 0.20

Barricarte (2007) | 12.941 7.167 23.368 0.20

Bettinger (2010) | 30.189 23.629 38.570 0.21

Black (2007) | 65.476 50.270 85.283 0.21

Bruce (2015) | 38.095 28.708 50.551 0.21

Byington (2005) | 25.714 17.634 37.497 0.21

Calbo (2006) | 21.875 12.955 36.936 0.20

Ceyhan (2011) | 22.603 16.069 31.794 0.21

Chiba (2014) | 45.033 38.066 53.275 0.21

Croney (2013) | 39.490 30.788 50.652 0.21

De Wals (2012) | 36.283 26.716 49.277 0.21

Guevara (2014) | 21.698 14.419 32.652 0.21

Hsu (2010) | 33.846 25.187 45.482 0.21

Ishiwada (2014) | 48.485 29.703 79.143 0.20

Kaplan (2013) | 32.348 28.132 37.196 0.21

Kellner (2009) | 23.881 14.630 38.981 0.20

Knol (2015) | 47.656 37.079 61.250 0.21

Lepoutre (2015)* | 28.571 24.181 33.759 0.21

Miller (2011) | 30.303 25.953 35.382 0.21

Moore (2014) | 50.769 36.093 71.413 0.21

Munoz-Almagro (2011) | 20.000 13.617 29.374 0.21

Parra (2013) | 29.762 20.110 44.046 0.21

Perez-Trallero (2009 | 13.333 5.990 29.679 0.19

Picazo (2011) | 20.909 16.514 26.473 0.21

Pilishvili (2010) | 32.177 27.649 37.447 0.21

Rendi-Wagner (2009) | 2.778 0.391 19.720 0.12

Rodriguez (2011) | 25.137 20.491 30.836 0.21

Salleras (2009) | 22.083 16.871 28.906 0.21

Schutze (2004) | 22.667 14.091 36.462 0.20

Sharma (2013)* | 40.426 25.785 63.378 0.20

Steens (2013) | 30.909 23.490 40.671 0.21

Suga (2015) | 31.169 25.518 38.071 0.21

Varon (2015) | 32.474 27.271 38.670 0.21

Vila-Corcoles (2013) | 15.385 8.278 28.593 0.20

Weatherholtz (2010) | 23.478 16.101 34.236 0.21

Williams (2011) | 32.836 25.797 41.795 0.21

van der Linden (2015 | 31.919 27.500 37.048 0.21

Sub-total |

D+L pooled ES | 29.442 26.773 32.377 7.77

I-V pooled ES | 31.417 30.138 32.750

---------------------+---------------------------------------------------

pcv7

Aguiar (2008) | 30.000 20.573 43.746 0.21

Aristegui (2007) | 18.182 10.768 30.700 0.20

Barricarte (2007) | 41.176 29.564 57.350 0.21

Bettinger (2010) | 18.396 13.441 25.179 0.21

Black (2007) | 11.905 6.405 22.126 0.20

Bruce (2015) | 2.381 0.768 7.382 0.17

Byington (2005) | 6.667 3.178 13.984 0.19

Calbo (2006) | 28.125 17.720 44.640 0.20

Ceyhan (2011) | 57.534 46.457 71.253 0.21

Chiba (2014) | 34.106 28.116 41.372 0.21

Croney (2013) | 12.102 7.719 18.973 0.20

De Wals (2012) | 4.425 1.842 10.631 0.18

Guevara (2014) | 9.434 5.076 17.534 0.20

Hsu (2010) | 11.538 6.956 19.140 0.20

Ishiwada (2014) | 15.152 6.306 36.403 0.18

Kaplan (2013) | 3.941 2.641 5.880 0.21

Kellner (2009) | 52.239 37.507 72.757 0.21

Knol (2015) | 3.906 1.626 9.385 0.18

Lepoutre (2015)* | 11.180 8.563 14.598 0.21

Miller (2011) | 7.955 5.879 10.764 0.21

Moore (2014) | 9.231 4.147 20.547 0.19

Munoz-Almagro (2011) | 6.923 3.602 13.306 0.20

Parra (2013) | 38.095 26.940 53.870 0.21

Perez-Trallero (2009 | 33.333 20.095 55.292 0.20

Picazo (2011) | 4.545 2.740 7.540 0.20

Pilishvili (2010) | 2.119 1.174 3.827 0.20

Rendi-Wagner (2009) | 75.000 51.433 109.365 0.21

Rodriguez (2011) | 4.918 3.099 7.806 0.20

Salleras (2009) | 31.667 25.291 39.650 0.21

Schutze (2004) | 6.667 2.775 16.017 0.18

Sharma (2013)* | 0.001 0.000 1.2e+35 0.00

Steens (2013) | 23.030 16.758 31.651 0.21

Suga (2015) | 36.364 30.216 43.762 0.21

Varon (2015) | 6.701 4.563 9.842 0.21

Vila-Corcoles (2013) | 35.385 23.514 53.248 0.21

Weatherholtz (2010) | 15.652 9.861 24.843 0.20

Williams (2011) | 18.905 13.756 25.982 0.21

van der Linden (2015 | 23.247 19.523 27.682 0.21

Sub-total |

D+L pooled ES | 14.756 11.405 19.090 7.44

I-V pooled ES | 22.217 20.969 23.539

---------------------+---------------------------------------------------

pcv10-pcv7

Aguiar (2008) | 25.556 16.982 38.457 0.21

Aristegui (2007) | 10.390 5.196 20.775 0.19

Barricarte (2007) | 7.059 3.171 15.712 0.19

Bettinger (2010) | 18.868 13.840 25.723 0.21

Black (2007) | 4.762 1.787 12.688 0.18

Bruce (2015) | 19.841 13.407 29.364 0.21

Byington (2005) | 10.476 5.802 18.917 0.20

Calbo (2006) | 20.313 11.794 34.982 0.20

Ceyhan (2011) | 7.534 4.172 13.605 0.20

Chiba (2014) | 0.993 0.320 3.080 0.17

Croney (2013) | 11.465 7.223 18.197 0.20

De Wals (2012) | 8.850 4.761 16.448 0.20

Guevara (2014) | 30.189 21.349 42.689 0.21

Hsu (2010) | 13.846 8.724 21.977 0.20

Ishiwada (2014) | 3.030 0.427 21.513 0.12

Kaplan (2013) | 15.599 12.758 19.074 0.21

Kellner (2009) | 7.463 3.106 17.930 0.18

Knol (2015) | 21.094 14.466 30.759 0.21

Lepoutre (2015)* | 27.329 23.043 32.413 0.21

Miller (2011) | 33.523 28.931 38.844 0.21

Moore (2014) | 23.077 13.912 38.279 0.20

Munoz-Almagro (2011) | 47.692 37.183 61.172 0.21

Parra (2013) | 10.714 5.575 20.592 0.20

Perez-Trallero (2009 | 35.556 21.782 58.038 0.20

Picazo (2011) | 50.000 42.924 58.242 0.21

Pilishvili (2010) | 9.827 7.468 12.930 0.21

Rendi-Wagner (2009) | 11.111 4.170 29.605 0.18

Rodriguez (2011) | 42.077 35.929 49.276 0.21

Salleras (2009) | 15.000 10.820 20.795 0.21

Schutze (2004) | 17.333 10.065 29.852 0.20

Sharma (2013)* | 8.511 3.194 22.676 0.18

Steens (2013) | 23.636 17.269 32.351 0.21

Suga (2015) | 0.649 0.162 2.596 0.15

Varon (2015) | 22.423 18.173 27.666 0.21

Vila-Corcoles (2013) | 38.462 25.989 56.921 0.21

Weatherholtz (2010) | 28.696 20.400 40.364 0.21

Williams (2011) | 2.488 1.035 5.977 0.18

van der Linden (2015 | 25.277 21.379 29.884 0.21

Sub-total |

D+L pooled ES | 16.329 13.472 19.793 7.48

I-V pooled ES | 25.730 24.466 27.058

---------------------+---------------------------------------------------

pcv13

Aguiar (2008) | 85.556 68.429 106.968 0.21

Aristegui (2007) | 88.312 69.629 112.007 0.21

Barricarte (2007) | 87.059 69.320 109.336 0.21

Bettinger (2010) | 69.811 59.423 82.015 0.21

Black (2007) | 34.524 23.991 49.680 0.21

Bruce (2015) | 61.905 49.584 77.287 0.21

Byington (2005) | 74.286 59.501 92.744 0.21

Calbo (2006) | 78.125 59.212 103.079 0.21

Ceyhan (2011) | 77.397 64.365 93.068 0.21

Chiba (2014) | 54.967 47.210 63.998 0.21

Croney (2013) | 60.510 49.487 73.987 0.21

De Wals (2012) | 63.717 50.575 80.273 0.21

Guevara (2014) | 78.302 63.145 97.097 0.21

Hsu (2010) | 66.154 53.551 81.723 0.21

Ishiwada (2014) | 51.515 32.025 82.868 0.20

Kaplan (2013) | 67.652 61.425 74.510 0.21

Kellner (2009) | 76.119 57.850 100.159 0.21

Knol (2015) | 52.344 41.198 66.506 0.21

Lepoutre (2015)* | 71.429 64.275 79.378 0.21

Miller (2011) | 69.697 62.928 77.195 0.21

Moore (2014) | 49.231 34.815 69.616 0.21

Munoz-Almagro (2011) | 80.000 66.012 96.952 0.21

Parra (2013) | 70.238 54.419 90.655 0.21

Perez-Trallero (2009 | 86.667 63.321 118.619 0.21

Picazo (2011) | 79.091 70.055 89.293 0.21

Pilishvili (2010) | 67.823 61.095 75.291 0.21

Rendi-Wagner (2009) | 97.222 69.805 135.409 0.21

Rodriguez (2011) | 74.863 66.504 84.274 0.21

Salleras (2009) | 77.917 67.512 89.924 0.21

Schutze (2004) | 77.333 59.786 100.032 0.21

Sharma (2013)* | 59.574 41.133 86.283 0.21

Steens (2013) | 69.091 57.504 83.013 0.21

Suga (2015) | 68.831 60.162 78.749 0.21

Varon (2015) | 67.526 59.825 76.218 0.21

Vila-Corcoles (2013) | 84.615 64.964 110.212 0.21

Weatherholtz (2010) | 76.522 62.093 94.303 0.21

Williams (2011) | 67.164 56.738 79.506 0.21

van der Linden (2015 | 68.081 61.477 75.395 0.21

Sub-total |

D+L pooled ES | 70.471 67.583 73.482 8.01

I-V pooled ES | 70.328 68.431 72.277

---------------------+---------------------------------------------------

19A

Aguiar (2008) | 24.444 16.095 37.124 0.21

Aristegui (2007) | 0.001 0.000 7.6e+34 0.00

Barricarte (2007) | 22.353 14.258 35.044 0.20

Bettinger (2010) | 20.283 15.043 27.349 0.21

Black (2007) | 11.905 6.405 22.126 0.20

Bruce (2015) | 31.746 23.286 43.279 0.21

Byington (2005) | 0.000 0.000 5.6e+34 0.00

Calbo (2006) | 0.001 0.000 9.1e+34 0.00

Ceyhan (2011) | 6.164 3.207 11.848 0.20

Chiba (2014) | 15.894 11.978 21.091 0.21

Croney (2013) | 32.484 24.687 42.743 0.21

De Wals (2012) | 43.363 32.773 57.375 0.21

Guevara (2014) | 32.075 22.919 44.891 0.21

Hsu (2010) | 23.846 16.770 33.908 0.21

Ishiwada (2014) | 27.273 14.190 52.416 0.20

Kaplan (2013) | 40.066 35.341 45.422 0.21

Kellner (2009) | 4.478 1.444 13.883 0.17

Knol (2015) | 22.656 15.744 32.603 0.21

Lepoutre (2015)* | 27.536 23.232 32.637 0.21

Miller (2011) | 18.939 15.568 23.040 0.21

Moore (2014) | 13.846 7.204 26.611 0.20

Munoz-Almagro (2011) | 20.000 13.617 29.374 0.21

Parra (2013) | 5.952 2.478 14.301 0.18

Perez-Trallero (2009 | 13.333 5.990 29.679 0.19

Picazo (2011) | 18.788 14.648 24.098 0.21

Pilishvili (2010) | 47.206 41.650 53.503 0.21

Rendi-Wagner (2009) | 0.001 0.000 1.6e+35 0.00

Rodriguez (2011) | 22.404 18.044 27.819 0.21

Salleras (2009) | 22.083 16.871 28.906 0.21

Schutze (2004) | 0.001 0.000 7.8e+34 0.00

Sharma (2013)* | 48.936 32.519 73.641 0.21

Steens (2013) | 10.303 6.405 16.574 0.20

Suga (2015) | 27.922 22.603 34.493 0.21

Varon (2015) | 32.990 27.742 39.230 0.21

Vila-Corcoles (2013) | 10.769 5.134 22.590 0.19

Weatherholtz (2010) | 19.130 12.596 29.054 0.21

Williams (2011) | 36.816 29.315 46.237 0.21

van der Linden (2015 | 9.225 6.992 12.172 0.21

Sub-total |

D+L pooled ES | 21.790 18.554 25.590 6.73

I-V pooled ES | 28.139 26.858 29.481

---------------------+---------------------------------------------------

6A

Aguiar (2008) | 2.222 0.556 8.886 0.15

Aristegui (2007) | 0.001 0.000 7.6e+34 0.00

Barricarte (2007) | 7.059 3.171 15.712 0.19

Bettinger (2010) | 4.245 2.209 8.159 0.20

Black (2007) | 3.571 1.152 11.074 0.17

Bruce (2015) | 2.381 0.768 7.382 0.17

Byington (2005) | 0.000 0.000 5.6e+34 0.00

Calbo (2006) | 9.375 4.212 20.868 0.19

Ceyhan (2011) | 2.055 0.663 6.371 0.17

Chiba (2014) | 1.987 0.893 4.422 0.19

Croney (2013) | 0.637 0.090 4.522 0.12

De Wals (2012) | 2.655 0.856 8.232 0.17

Guevara (2014) | 1.887 0.472 7.544 0.15

Hsu (2010) | 12.308 7.540 20.090 0.20

Ishiwada (2014) | 3.030 0.427 21.513 0.12

Kaplan (2013) | 0.328 0.082 1.313 0.15

Kellner (2009) | 2.985 0.747 11.936 0.15

Knol (2015) | 1.563 0.391 6.248 0.15

Lepoutre (2015)* | 1.449 0.691 3.040 0.19

Miller (2011) | 0.000 0.000 1.1e+34 0.00

Moore (2014) | 1.538 0.217 10.922 0.12

Munoz-Almagro (2011) | 2.308 0.744 7.155 0.17

Parra (2013) | 1.190 0.168 8.452 0.12

Perez-Trallero (2009 | 4.444 1.112 17.771 0.15

Picazo (2011) | 1.212 0.455 3.230 0.18

Pilishvili (2010) | 0.000 0.000 1.1e+34 0.00

Rendi-Wagner (2009) | 8.333 2.688 25.839 0.17

Rodriguez (2011) | 2.186 1.093 4.371 0.19

Salleras (2009) | 7.083 4.403 11.394 0.20

Schutze (2004) | 0.001 0.000 7.8e+34 0.00

Sharma (2013)* | 0.001 0.000 1.2e+35 0.00

Steens (2013) | 5.455 2.838 10.483 0.20

Suga (2015) | 2.597 1.299 5.194 0.19

Varon (2015) | 0.773 0.249 2.397 0.17

Vila-Corcoles (2013) | 0.001 0.000 9.0e+34 0.00

Weatherholtz (2010) | 4.348 1.810 10.446 0.18

Williams (2011) | 4.478 2.330 8.606 0.20

van der Linden (2015 | 4.244 2.820 6.386 0.21

Sub-total |

D+L pooled ES | 3.094 2.358 4.060 5.23

I-V pooled ES | 3.866 3.327 4.493

---------------------+---------------------------------------------------

3

Aguiar (2008) | 3.333 1.075 10.335 0.17

Aristegui (2007) | 2.597 0.650 10.386 0.15

Barricarte (2007) | 5.882 2.448 14.133 0.18a

Bettinger (2010) | 8.019 4.985 12.899 0.20

Black (2007) | 2.381 0.595 9.520 0.15

Bruce (2015) | 5.556 2.648 11.654 0.19

Byington (2005) | 9.524 5.124 17.701 0.20

Calbo (2006) | 0.001 0.000 9.1e+34 0.00

Ceyhan (2011) | 4.110 1.846 9.148 0.19

Chiba (2014) | 1.987 0.893 4.422 0.19

Croney (2013) | 3.822 1.717 8.507 0.19

De Wals (2012) | 4.425 1.842 10.631 0.18

Guevara (2014) | 4.717 1.963 11.333 0.18

Hsu (2010) | 4.615 2.073 10.273 0.19

Ishiwada (2014) | 3.030 0.427 21.513 0.12

Kaplan (2013) | 7.718 5.799 10.272 0.21

Kellner (2009) | 8.955 4.023 19.934 0.19

Knol (2015) | 3.125 1.173 8.326 0.18

Lepoutre (2015)* | 3.934 2.509 6.167 0.20

Miller (2011) | 5.682 3.973 8.126 0.21

Moore (2014) | 0.001 0.000 9.0e+34 0.00

Munoz-Almagro (2011) | 3.077 1.155 8.198 0.18

Parra (2013) | 14.286 8.113 25.155 0.20

Perez-Trallero (2009 | 0.001 0.000 1.3e+35 0.00

Picazo (2011) | 3.939 2.287 6.784 0.20

Pilishvili (2010) | 5.010 3.411 7.358 0.21

Rendi-Wagner (2009) | 2.778 0.391 19.720 0.12

Rodriguez (2011) | 3.279 1.862 5.773 0.20

Salleras (2009) | 2.083 0.867 5.005 0.18

Schutze (2004) | 1.333 0.188 9.466 0.12

Sharma (2013)* | 2.128 0.300 15.105 0.12

Steens (2013) | 6.667 3.692 12.038 0.20

Suga (2015) | 1.299 0.487 3.460 0.18

Varon (2015) | 4.639 2.923 7.363 0.20

Vila-Corcoles (2013) | 0.001 0.000 9.0e+34 0.00

Weatherholtz (2010) | 8.696 4.679 16.161 0.20

Williams (2011) | 4.478 2.330 8.606 0.20

van der Linden (2015 | 6.089 4.328 8.564 0.21

Sub-total |

D+L pooled ES | 4.943 4.217 5.794 6.15

I-V pooled ES | 5.388 4.850 5.986

---------------------+---------------------------------------------------

12F

Aguiar (2008) | 0.001 0.000 6.5e+34 0.00

Aristegui (2007) | 0.001 0.000 7.6e+34 0.00

Barricarte (2007) | 0.001 0.000 6.9e+34 0.00

Bettinger (2010) | 0.000 0.000 2.8e+34 0.00

Black (2007) | 1.190 0.168 8.452 0.12

Bruce (2015) | 4.762 2.139 10.600 0.19

Byington (2005) | 0.000 0.000 5.6e+34 0.00

Calbo (2006) | 0.001 0.000 9.1e+34 0.00

Ceyhan (2011) | 0.000 0.000 4.0e+34 0.00

Chiba (2014) | 0.000 0.000 1.9e+34 0.00

Croney (2013) | 0.000 0.000 3.7e+34 0.00

De Wals (2012) | 0.000 0.000 5.2e+34 0.00

Guevara (2014) | 0.943 0.133 6.697 0.12

Hsu (2010) | 1.538 0.385 6.152 0.15

Ishiwada (2014) | 0.002 0.000 1.8e+35 0.00

Kaplan (2013) | 0.000 0.000 9.6e+33 0.00

Kellner (2009) | 0.001 0.000 8.7e+34 0.00

Knol (2015) | 1.563 0.391 6.248 0.15

Lepoutre (2015)* | 1.863 0.970 3.581 0.20

Miller (2011) | 1.515 0.758 3.030 0.19

Moore (2014) | 3.077 0.770 12.303 0.15

Munoz-Almagro (2011) | 0.769 0.108 5.461 0.12

Parra (2013) | 0.001 0.000 7.0e+34 0.00

Perez-Trallero (2009 | 0.001 0.000 1.3e+35 0.00

Picazo (2011) | 1.515 0.631 3.640 0.18

Pilishvili (2010) | 1.541 0.771 3.082 0.19

Rendi-Wagner (2009) | 0.001 0.000 1.6e+35 0.00

Rodriguez (2011) | 2.186 1.093 4.371 0.19

Salleras (2009) | 2.917 1.390 6.118 0.19

Schutze (2004) | 0.001 0.000 7.8e+34 0.00

Sharma (2013)* | 2.128 0.300 15.105 0.12

Steens (2013) | 0.000 0.000 3.5e+34 0.00

Suga (2015) | 0.325 0.046 2.305 0.12

Varon (2015) | 2.320 1.207 4.458 0.20

Vila-Corcoles (2013) | 3.077 0.770 12.303 0.15

Weatherholtz (2010) | 9.565 5.297 17.272 0.20

Williams (2011) | 0.000 0.000 2.9e+34 0.00

van der Linden (2015 | 1.292 0.616 2.709 0.19

Sub-total |

D+L pooled ES | 2.280 1.811 2.871 3.10

I-V pooled ES | 2.342 1.907 2.876

---------------------+---------------------------------------------------

10A

Aguiar (2008) | 2.222 0.556 8.886 0.15

Aristegui (2007) | 0.001 0.000 7.6e+34 0.00

Barricarte (2007) | 0.001 0.000 6.9e+34 0.00

Bettinger (2010) | 1.887 0.708 5.027 0.18

Black (2007) | 3.571 1.152 11.074 0.17

Bruce (2015) | 1.587 0.397 6.347 0.15

Byington (2005) | 0.000 0.000 5.6e+34 0.00

Calbo (2006) | 0.001 0.000 9.1e+34 0.00

Ceyhan (2011) | 0.000 0.000 4.0e+34 0.00

Chiba (2014) | 0.993 0.320 3.080 0.17

Croney (2013) | 0.000 0.000 3.7e+34 0.00

De Wals (2012) | 2.655 0.856 8.232 0.17

Guevara (2014) | 1.887 0.472 7.544 0.15

Hsu (2010) | 1.538 0.385 6.152 0.15

Ishiwada (2014) | 6.061 1.516 24.234 0.15

Kaplan (2013) | 0.000 0.000 9.6e+33 0.00

Kellner (2009) | 0.001 0.000 8.7e+34 0.00

Knol (2015) | 10.938 6.478 18.468 0.20

Lepoutre (2015)* | 0.000 0.000 1.2e+34 0.00

Miller (2011) | 1.136 0.511 2.529 0.19

Moore (2014) | 0.001 0.000 9.0e+34 0.00

Munoz-Almagro (2011) | 2.308 0.744 7.155 0.17

Parra (2013) | 0.001 0.000 7.0e+34 0.00

Perez-Trallero (2009 | 0.001 0.000 1.3e+35 0.00

Picazo (2011) | 0.909 0.293 2.819 0.17

Pilishvili (2010) | 2.697 1.598 4.555 0.20

Rendi-Wagner (2009) | 0.001 0.000 1.6e+35 0.00

Rodriguez (2011) | 1.913 0.912 4.012 0.19

Salleras (2009) | 2.500 1.123 5.565 0.19

Schutze (2004) | 0.001 0.000 7.8e+34 0.00

Sharma (2013)* | 0.001 0.000 1.2e+35 0.00

Steens (2013) | 2.424 0.910 6.459 0.18

Suga (2015) | 2.922 1.520 5.616 0.20

Varon (2015) | 1.804 0.860 3.784 0.19

Vila-Corcoles (2013) | 1.538 0.217 10.922 0.12

Weatherholtz (2010) | 2.609 0.841 8.089 0.17

Williams (2011) | 0.000 0.000 2.9e+34 0.00

van der Linden (2015 | 4.797 3.266 7.045 0.21

Sub-total |

D+L pooled ES | 2.580 2.006 3.318 3.78

I-V pooled ES | 3.020 2.536 3.596

---------------------+---------------------------------------------------

22F

Aguiar (2008) | 2.222 0.556 8.886 0.15

Aristegui (2007) | 0.001 0.000 7.6e+34 0.00

Barricarte (2007) | 0.001 0.000 6.9e+34 0.00

Bettinger (2010) | 6.132 3.561 10.561 0.20

Black (2007) | 9.524 4.763 19.044 0.19

Bruce (2015) | 3.968 1.652 9.534 0.18

Byington (2005) | 0.000 0.000 5.6e+34 0.00

Calbo (2006) | 0.001 0.000 9.1e+34 0.00

Ceyhan (2011) | 0.000 0.000 4.0e+34 0.00

Chiba (2014) | 4.636 2.746 7.827 0.20

Croney (2013) | 0.000 0.000 3.7e+34 0.00

De Wals (2012) | 4.425 1.842 10.631 0.18

Guevara (2014) | 1.887 0.472 7.544 0.15

Hsu (2010) | 6.154 3.077 12.305 0.19

Ishiwada (2014) | 6.061 1.516 24.234 0.15

Kaplan (2013) | 3.448 2.248 5.289 0.21

Kellner (2009) | 5.970 2.241 15.907 0.18

Knol (2015) | 3.906 1.626 9.385 0.18

Lepoutre (2015)* | 1.863 0.970 3.581 0.20

Miller (2011) | 5.682 3.973 8.126 0.21

Moore (2014) | 4.615 1.489 14.311 0.17

Munoz-Almagro (2011) | 0.769 0.108 5.461 0.12

Parra (2013) | 1.190 0.168 8.452 0.12

Perez-Trallero (2009 | 0.001 0.000 1.3e+35 0.00

Picazo (2011) | 0.606 0.152 2.423 0.15

Pilishvili (2010) | 4.817 3.255 7.129 0.21

Rendi-Wagner (2009) | 0.001 0.000 1.6e+35 0.00

Rodriguez (2011) | 1.093 0.410 2.912 0.18

Salleras (2009) | 0.417 0.059 2.958 0.12

Schutze (2004) | 0.001 0.000 7.8e+34 0.00

Sharma (2013)* | 4.255 1.064 17.015 0.15

Steens (2013) | 5.455 2.838 10.483 0.20

Suga (2015) | 2.273 1.083 4.767 0.19

Varon (2015) | 1.546 0.695 3.442 0.19

Vila-Corcoles (2013) | 1.538 0.217 10.922 0.12

Weatherholtz (2010) | 3.478 1.305 9.268 0.18

Williams (2011) | 4.478 2.330 8.606 0.20

van der Linden (2015 | 1.845 0.993 3.429 0.20

Sub-total |

D+L pooled ES | 3.512 2.859 4.316 5.03

I-V pooled ES | 3.900 3.410 4.461

---------------------+---------------------------------------------------

15B

Aguiar (2008) | 1.111 0.157 7.888 0.12

Aristegui (2007) | 0.001 0.000 7.6e+34 0.00

Barricarte (2007) | 1.176 0.166 8.352 0.12

Bettinger (2010) | 2.830 1.271 6.300 0.19

Black (2007) | 1.190 0.168 8.452 0.12

Bruce (2015) | 0.794 0.112 5.634 0.12

Byington (2005) | 0.000 0.000 5.6e+34 0.00

Calbo (2006) | 0.001 0.000 9.1e+34 0.00

Ceyhan (2011) | 0.000 0.000 4.0e+34 0.00

Chiba (2014) | 3.311 1.782 6.154 0.20

Croney (2013) | 0.000 0.000 3.7e+34 0.00

De Wals (2012) | 1.770 0.443 7.077 0.15

Guevara (2014) | 0.943 0.133 6.697 0.12

Hsu (2010) | 0.000 0.000 4.5e+34 0.00

Ishiwada (2014) | 0.002 0.000 1.8e+35 0.00

Kaplan (2013) | 2.627 1.610 4.289 0.20

Kellner (2009) | 0.001 0.000 8.7e+34 0.00

Knol (2015) | 2.344 0.756 7.267 0.17

Lepoutre (2015)* | 1.863 0.970 3.581 0.20

Miller (2011) | 2.462 1.430 4.240 0.20

Moore (2014) | 1.538 0.217 10.922 0.12

Munoz-Almagro (2011) | 0.769 0.108 5.461 0.12

Parra (2013) | 3.571 1.152 11.074 0.17

Perez-Trallero (2009 | 0.001 0.000 1.3e+35 0.00

Picazo (2011) | 1.818 0.817 4.047 0.19

Pilishvili (2010) | 0.000 0.000 1.1e+34 0.00

Rendi-Wagner (2009) | 0.001 0.000 1.6e+35 0.00

Rodriguez (2011) | 3.279 1.862 5.773 0.20

Salleras (2009) | 0.000 0.000 2.4e+34 0.00

Schutze (2004) | 0.001 0.000 7.8e+34 0.00

Sharma (2013)* | 0.001 0.000 1.2e+35 0.00

Steens (2013) | 0.000 0.000 3.5e+34 0.00

Suga (2015) | 2.273 1.083 4.767 0.19

Varon (2015) | 0.000 0.000 1.5e+34 0.00

Vila-Corcoles (2013) | 0.001 0.000 9.0e+34 0.00

Weatherholtz (2010) | 0.000 0.000 5.1e+34 0.00

Williams (2011) | 0.000 0.000 2.9e+34 0.00

van der Linden (2015 | 2.768 1.668 4.591 0.20

Sub-total |

D+L pooled ES | 2.448 2.029 2.954 3.06

I-V pooled ES | 2.448 2.029 2.954

---------------------+---------------------------------------------------

15C

Aguiar (2008) | 0.001 0.000 6.5e+34 0.00

Aristegui (2007) | 0.001 0.000 7.6e+34 0.00

Barricarte (2007) | 2.353 0.588 9.408 0.15

Bettinger (2010) | 1.415 0.456 4.388 0.17

Black (2007) | 7.143 3.209 15.899 0.19

Bruce (2015) | 0.794 0.112 5.634 0.12

Byington (2005) | 0.000 0.000 5.6e+34 0.00

Calbo (2006) | 1.563 0.220 11.093 0.12

Ceyhan (2011) | 1.370 0.343 5.477 0.15

Chiba (2014) | 4.967 2.994 8.239 0.20

Croney (2013) | 0.000 0.000 3.7e+34 0.00

De Wals (2012) | 2.655 0.856 8.232 0.17

Guevara (2014) | 0.000 0.000 5.5e+34 0.00

Hsu (2010) | 0.000 0.000 4.5e+34 0.00

Ishiwada (2014) | 3.030 0.427 21.513 0.12

Kaplan (2013) | 1.970 1.119 3.470 0.20

Kellner (2009) | 0.001 0.000 8.7e+34 0.00

Knol (2015) | 0.781 0.110 5.546 0.12

Lepoutre (2015)* | 1.242 0.558 2.765 0.19

Miller (2011) | 2.462 1.430 4.240 0.20

Moore (2014) | 6.154 2.310 16.397 0.18

Munoz-Almagro (2011) | 0.000 0.000 4.5e+34 0.00

Parra (2013) | 2.381 0.595 9.520 0.15

Perez-Trallero (2009 | 0.001 0.000 1.3e+35 0.00

Picazo (2011) | 0.606 0.152 2.423 0.15

Pilishvili (2010) | 0.000 0.000 1.1e+34 0.00

Rendi-Wagner (2009) | 0.001 0.000 1.6e+35 0.00

Rodriguez (2011) | 1.093 0.410 2.912 0.18

Salleras (2009) | 0.000 0.000 2.4e+34 0.00

Schutze (2004) | 0.001 0.000 7.8e+34 0.00

Sharma (2013)* | 0.001 0.000 1.2e+35 0.00

Steens (2013) | 0.000 0.000 3.5e+34 0.00

Suga (2015) | 4.545 2.692 7.675 0.20

Varon (2015) | 0.000 0.000 1.5e+34 0.00

Vila-Corcoles (2013) | 3.077 0.770 12.303 0.15

Weatherholtz (2010) | 0.000 0.000 5.1e+34 0.00

Williams (2011) | 0.000 0.000 2.9e+34 0.00

van der Linden (2015 | 2.952 1.808 4.819 0.20

Sub-total |

D+L pooled ES | 2.804 2.326 3.380 3.28

I-V pooled ES | 2.804 2.326 3.380

---------------------+---------------------------------------------------

24F

Aguiar (2008) | 1.111 0.157 7.888 0.12

Aristegui (2007) | 0.001 0.000 7.6e+34 0.00

Barricarte (2007) | 0.001 0.000 6.9e+34 0.00

Bettinger (2010) | 0.000 0.000 2.8e+34 0.00

Black (2007) | 0.001 0.000 7.0e+34 0.00

Bruce (2015) | 0.000 0.000 4.6e+34 0.00

Byington (2005) | 0.000 0.000 5.6e+34 0.00

Calbo (2006) | 0.001 0.000 9.1e+34 0.00

Ceyhan (2011) | 0.000 0.000 4.0e+34 0.00

Chiba (2014) | 0.000 0.000 1.9e+34 0.00

Croney (2013) | 0.000 0.000 3.7e+34 0.00

De Wals (2012) | 0.000 0.000 5.2e+34 0.00

Guevara (2014) | 3.774 1.416 10.055 0.18

Hsu (2010) | 0.000 0.000 4.5e+34 0.00

Ishiwada (2014) | 6.061 1.516 24.234 0.15

Kaplan (2013) | 0.000 0.000 9.6e+33 0.00

Kellner (2009) | 0.001 0.000 8.7e+34 0.00

Knol (2015) | 2.344 0.756 7.267 0.17

Lepoutre (2015)* | 4.555 2.999 6.918 0.21

Miller (2011) | 0.568 0.183 1.762 0.17

Moore (2014) | 0.001 0.000 9.0e+34 0.00

Munoz-Almagro (2011) | 4.615 2.073 10.273 0.19

Parra (2013) | 1.190 0.168 8.452 0.12

Perez-Trallero (2009 | 0.001 0.000 1.3e+35 0.00

Picazo (2011) | 1.515 0.631 3.640 0.18

Pilishvili (2010) | 0.000 0.000 1.1e+34 0.00

Rendi-Wagner (2009) | 0.001 0.000 1.6e+35 0.00

Rodriguez (2011) | 1.913 0.912 4.012 0.19

Salleras (2009) | 4.583 2.538 8.276 0.20

Schutze (2004) | 0.001 0.000 7.8e+34 0.00

Sharma (2013)* | 0.001 0.000 1.2e+35 0.00

Steens (2013) | 0.000 0.000 3.5e+34 0.00

Suga (2015) | 4.870 2.936 8.078 0.20

Varon (2015) | 6.443 4.354 9.536 0.21

Vila-Corcoles (2013) | 0.001 0.000 9.0e+34 0.00

Weatherholtz (2010) | 0.000 0.000 5.1e+34 0.00

Williams (2011) | 0.000 0.000 2.9e+34 0.00

van der Linden (2015 | 3.875 2.526 5.943 0.21

Sub-total |

D+L pooled ES | 3.998 3.357 4.761 2.47

I-V pooled ES | 3.998 3.357 4.761

---------------------+---------------------------------------------------

15A

Aguiar (2008) | 0.001 0.000 6.5e+34 0.00

Aristegui (2007) | 0.001 0.000 7.6e+34 0.00

Barricarte (2007) | 0.001 0.000 6.9e+34 0.00

Bettinger (2010) | 0.943 0.236 3.772 0.15

Black (2007) | 0.001 0.000 7.0e+34 0.00

Bruce (2015) | 2.381 0.768 7.382 0.17

Byington (2005) | 0.000 0.000 5.6e+34 0.00

Calbo (2006) | 0.001 0.000 9.1e+34 0.00

Ceyhan (2011) | 0.000 0.000 4.0e+34 0.00

Chiba (2014) | 6.623 4.273 10.265 0.20

Croney (2013) | 0.000 0.000 3.7e+34 0.00

De Wals (2012) | 2.655 0.856 8.232 0.17

Guevara (2014) | 0.943 0.133 6.697 0.12

Hsu (2010) | 0.000 0.000 4.5e+34 0.00

Ishiwada (2014) | 15.152 6.306 36.403 0.18

Kaplan (2013) | 0.985 0.443 2.193 0.19

Kellner (2009) | 0.001 0.000 8.7e+34 0.00

Knol (2015) | 0.781 0.110 5.546 0.12

Lepoutre (2015)* | 1.863 0.970 3.581 0.20

Miller (2011) | 0.947 0.394 2.275 0.18

Moore (2014) | 1.538 0.217 10.922 0.12

Munoz-Almagro (2011) | 0.000 0.000 4.5e+34 0.00

Parra (2013) | 2.381 0.595 9.520 0.15

Perez-Trallero (2009 | 0.001 0.000 1.3e+35 0.00

Picazo (2011) | 0.606 0.152 2.423 0.15

Pilishvili (2010) | 1.541 0.771 3.082 0.19

Rendi-Wagner (2009) | 0.001 0.000 1.6e+35 0.00

Rodriguez (2011) | 0.820 0.264 2.542 0.17

Salleras (2009) | 0.417 0.059 2.958 0.12

Schutze (2004) | 0.001 0.000 7.8e+34 0.00

Sharma (2013)* | 0.001 0.000 1.2e+35 0.00

Steens (2013) | 0.000 0.000 3.5e+34 0.00

Suga (2015) | 5.844 3.682 9.276 0.20

Varon (2015) | 1.546 0.695 3.442 0.19

Vila-Corcoles (2013) | 0.001 0.000 9.0e+34 0.00

Weatherholtz (2010) | 0.000 0.000 5.1e+34 0.00

Williams (2011) | 0.000 0.000 2.9e+34 0.00

van der Linden (2015 | 0.369 0.092 1.475 0.15

Sub-total |

D+L pooled ES | 1.781 1.168 2.715 3.10

I-V pooled ES | 2.697 2.212 3.287

---------------------+---------------------------------------------------

33F

Aguiar (2008) | 1.111 0.157 7.888 0.12

Aristegui (2007) | 0.001 0.000 7.6e+34 0.00

Barricarte (2007) | 0.001 0.000 6.9e+34 0.00

Bettinger (2010) | 1.887 0.708 5.027 0.18

Black (2007) | 4.762 1.787 12.688 0.18

Bruce (2015) | 3.175 1.191 8.459 0.18

Byington (2005) | 0.000 0.000 5.6e+34 0.00

Calbo (2006) | 0.001 0.000 9.1e+34 0.00

Ceyhan (2011) | 0.000 0.000 4.0e+34 0.00

Chiba (2014) | 1.987 0.893 4.422 0.19

Croney (2013) | 0.000 0.000 3.7e+34 0.00

De Wals (2012) | 3.540 1.329 9.432 0.18

Guevara (2014) | 1.887 0.472 7.544 0.15

Hsu (2010) | 5.385 2.567 11.295 0.19

Ishiwada (2014) | 6.061 1.516 24.234 0.15

Kaplan (2013) | 0.000 0.000 9.6e+33 0.00

Kellner (2009) | 0.001 0.000 8.7e+34 0.00

Knol (2015) | 8.594 4.759 15.518 0.20

Lepoutre (2015)* | 0.000 0.000 1.2e+34 0.00

Miller (2011) | 3.598 2.295 5.642 0.20

Moore (2014) | 13.846 7.204 26.611 0.20

Munoz-Almagro (2011) | 0.000 0.000 4.5e+34 0.00

Parra (2013) | 0.001 0.000 7.0e+34 0.00

Perez-Trallero (2009 | 0.001 0.000 1.3e+35 0.00

Picazo (2011) | 0.303 0.043 2.151 0.12

Pilishvili (2010) | 4.817 3.255 7.129 0.21

Rendi-Wagner (2009) | 0.001 0.000 1.6e+35 0.00

Rodriguez (2011) | 0.000 0.000 1.6e+34 0.00

Salleras (2009) | 2.083 0.867 5.005 0.18

Schutze (2004) | 0.001 0.000 7.8e+34 0.00

Sharma (2013)* | 12.766 5.735 28.416 0.19

Steens (2013) | 5.455 2.838 10.483 0.20

Suga (2015) | 1.948 0.875 4.336 0.19

Varon (2015) | 1.289 0.536 3.096 0.18

Vila-Corcoles (2013) | 0.001 0.000 9.0e+34 0.00

Weatherholtz (2010) | 0.000 0.000 5.1e+34 0.00

Williams (2011) | 1.493 0.481 4.628 0.17

van der Linden (2015 | 1.292 0.616 2.709 0.19

Sub-total |

D+L pooled ES | 3.423 2.554 4.588 3.71

I-V pooled ES | 3.927 3.327 4.634

---------------------+---------------------------------------------------

6C

Aguiar (2008) | 0.001 0.000 6.5e+34 0.00

Aristegui (2007) | 0.001 0.000 7.6e+34 0.00

Barricarte (2007) | 0.001 0.000 6.9e+34 0.00

Bettinger (2010) | 0.000 0.000 2.8e+34 0.00

Black (2007) | 0.001 0.000 7.0e+34 0.00

Bruce (2015) | 1.587 0.397 6.347 0.15

Byington (2005) | 0.000 0.000 5.6e+34 0.00

Calbo (2006) | 0.001 0.000 9.1e+34 0.00

Ceyhan (2011) | 0.000 0.000 4.0e+34 0.00

Chiba (2014) | 5.629 3.499 9.055 0.20

Croney (2013) | 4.459 2.126 9.353 0.19

De Wals (2012) | 1.770 0.443 7.077 0.15

Guevara (2014) | 1.887 0.472 7.544 0.15

Hsu (2010) | 0.000 0.000 4.5e+34 0.00

Ishiwada (2014) | 0.002 0.000 1.8e+35 0.00

Kaplan (2013) | 3.612 2.379 5.486 0.21

Kellner (2009) | 0.001 0.000 8.7e+34 0.00

Knol (2015) | 0.000 0.000 4.6e+34 0.00

Lepoutre (2015)* | 1.242 0.558 2.765 0.19

Miller (2011) | 0.000 0.000 1.1e+34 0.00

Moore (2014) | 3.077 0.770 12.303 0.15

Munoz-Almagro (2011) | 0.000 0.000 4.5e+34 0.00

Parra (2013) | 0.001 0.000 7.0e+34 0.00

Perez-Trallero (2009 | 0.001 0.000 1.3e+35 0.00

Picazo (2011) | 0.606 0.152 2.423 0.15

Pilishvili (2010) | 0.000 0.000 1.1e+34 0.00

Rendi-Wagner (2009) | 0.001 0.000 1.6e+35 0.00

Rodriguez (2011) | 0.000 0.000 1.6e+34 0.00

Salleras (2009) | 0.000 0.000 2.4e+34 0.00

Schutze (2004) | 0.001 0.000 7.8e+34 0.00

Sharma (2013)* | 0.001 0.000 1.2e+35 0.00

Steens (2013) | 1.212 0.303 4.847 0.15

Suga (2015) | 2.597 1.299 5.194 0.19

Varon (2015) | 1.031 0.387 2.747 0.18

Vila-Corcoles (2013) | 0.001 0.000 9.0e+34 0.00

Weatherholtz (2010) | 0.000 0.000 5.1e+34 0.00

Williams (2011) | 0.000 0.000 2.9e+34 0.00

van der Linden (2015 | 0.554 0.179 1.716 0.17

Sub-total |

D+L pooled ES | 2.761 2.215 3.442 2.23

I-V pooled ES | 2.761 2.215 3.442

---------------------+---------------------------------------------------

23B

Aguiar (2008) | 1.111 0.157 7.888 0.12

Aristegui (2007) | 0.001 0.000 7.6e+34 0.00

Barricarte (2007) | 2.353 0.588 9.408 0.15

Bettinger (2010) | 1.415 0.456 4.388 0.17

Black (2007) | 1.190 0.168 8.452 0.12

Bruce (2015) | 3.968 1.652 9.534 0.18

Byington (2005) | 0.000 0.000 5.6e+34 0.00

Calbo (2006) | 0.001 0.000 9.1e+34 0.00

Ceyhan (2011) | 0.000 0.000 4.0e+34 0.00

Chiba (2014) | 0.000 0.000 1.9e+34 0.00

Croney (2013) | 1.911 0.616 5.925 0.17

De Wals (2012) | 0.885 0.125 6.283 0.12

Guevara (2014) | 0.000 0.000 5.5e+34 0.00

Hsu (2010) | 0.000 0.000 4.5e+34 0.00

Ishiwada (2014) | 0.002 0.000 1.8e+35 0.00

Kaplan (2013) | 2.299 1.361 3.882 0.20

Kellner (2009) | 0.001 0.000 8.7e+34 0.00

Knol (2015) | 0.781 0.110 5.546 0.12

Lepoutre (2015)* | 0.000 0.000 1.2e+34 0.00

Miller (2011) | 1.326 0.632 2.781 0.19

Moore (2014) | 1.538 0.217 10.922 0.12

Munoz-Almagro (2011) | 2.308 0.744 7.155 0.17

Parra (2013) | 1.190 0.168 8.452 0.12

Perez-Trallero (2009 | 0.001 0.000 1.3e+35 0.00

Picazo (2011) | 0.909 0.293 2.819 0.17

Pilishvili (2010) | 1.349 0.643 2.829 0.19

Rendi-Wagner (2009) | 0.001 0.000 1.6e+35 0.00

Rodriguez (2011) | 2.186 1.093 4.371 0.19

Salleras (2009) | 2.500 1.123 5.565 0.19

Schutze (2004) | 0.001 0.000 7.8e+34 0.00

Sharma (2013)* | 2.128 0.300 15.105 0.12

Steens (2013) | 1.212 0.303 4.847 0.15

Suga (2015) | 0.000 0.000 1.9e+34 0.00

Varon (2015) | 0.773 0.249 2.397 0.17

Vila-Corcoles (2013) | 0.001 0.000 9.0e+34 0.00

Weatherholtz (2010) | 1.739 0.435 6.954 0.15

Williams (2011) | 0.000 0.000 2.9e+34 0.00

van der Linden (2015 | 0.554 0.179 1.716 0.17

Sub-total |

D+L pooled ES | 1.701 1.362 2.124 3.41

I-V pooled ES | 1.701 1.362 2.124

---------------------+---------------------------------------------------

11A

Aguiar (2008) | 0.001 0.000 6.5e+34 0.00

Aristegui (2007) | 0.001 0.000 7.6e+34 0.00

Barricarte (2007) | 0.001 0.000 6.9e+34 0.00

Bettinger (2010) | 1.415 0.456 4.388 0.17

Black (2007) | 2.381 0.595 9.520 0.15

Bruce (2015) | 0.794 0.112 5.634 0.12

Byington (2005) | 0.000 0.000 5.6e+34 0.00

Calbo (2006) | 0.001 0.000 9.1e+34 0.00

Ceyhan (2011) | 0.000 0.000 4.0e+34 0.00

Chiba (2014) | 0.000 0.000 1.9e+34 0.00

Croney (2013) | 0.000 0.000 3.7e+34 0.00

De Wals (2012) | 0.885 0.125 6.283 0.12

Guevara (2014) | 0.000 0.000 5.5e+34 0.00

Hsu (2010) | 0.000 0.000 4.5e+34 0.00

Ishiwada (2014) | 0.002 0.000 1.8e+35 0.00

Kaplan (2013) | 0.000 0.000 9.6e+33 0.00

Kellner (2009) | 0.001 0.000 8.7e+34 0.00

Knol (2015) | 2.344 0.756 7.267 0.17

Lepoutre (2015)* | 0.000 0.000 1.2e+34 0.00

Miller (2011) | 1.515 0.758 3.030 0.19

Moore (2014) | 0.001 0.000 9.0e+34 0.00

Munoz-Almagro (2011) | 0.000 0.000 4.5e+34 0.00

Parra (2013) | 2.381 0.595 9.520 0.15

Perez-Trallero (2009 | 0.001 0.000 1.3e+35 0.00

Picazo (2011) | 1.212 0.455 3.230 0.18

Pilishvili (2010) | 0.771 0.289 2.054 0.18

Rendi-Wagner (2009) | 0.001 0.000 1.6e+35 0.00

Rodriguez (2011) | 0.820 0.264 2.542 0.17

Salleras (2009) | 0.000 0.000 2.4e+34 0.00

Schutze (2004) | 0.001 0.000 7.8e+34 0.00

Sharma (2013)* | 0.001 0.000 1.2e+35 0.00

Steens (2013) | 0.000 0.000 3.5e+34 0.00

Suga (2015) | 0.649 0.162 2.596 0.15

Varon (2015) | 0.258 0.036 1.830 0.12

Vila-Corcoles (2013) | 1.538 0.217 10.922 0.12

Weatherholtz (2010) | 0.000 0.000 5.1e+34 0.00

Williams (2011) | 2.985 1.341 6.645 0.19

van der Linden (2015 | 0.554 0.179 1.716 0.17

Sub-total |

D+L pooled ES | 1.316 0.979 1.768 2.32

I-V pooled ES | 1.316 0.979 1.768

---------------------+---------------------------------------------------

35B

Aguiar (2008) | 0.001 0.000 6.5e+34 0.00

Aristegui (2007) | 0.001 0.000 7.6e+34 0.00

Barricarte (2007) | 0.001 0.000 6.9e+34 0.00

Bettinger (2010) | 0.943 0.236 3.772 0.15

Black (2007) | 1.190 0.168 8.452 0.12

Bruce (2015) | 1.587 0.397 6.347 0.15

Byington (2005) | 0.000 0.000 5.6e+34 0.00

Calbo (2006) | 0.001 0.000 9.1e+34 0.00

Ceyhan (2011) | 0.000 0.000 4.0e+34 0.00

Chiba (2014) | 1.987 0.893 4.422 0.19

Croney (2013) | 3.185 1.326 7.651 0.18

De Wals (2012) | 0.000 0.000 5.2e+34 0.00

Guevara (2014) | 0.000 0.000 5.5e+34 0.00

Hsu (2010) | 0.000 0.000 4.5e+34 0.00

Ishiwada (2014) | 3.030 0.427 21.513 0.12

Kaplan (2013) | 0.000 0.000 9.6e+33 0.00

Kellner (2009) | 0.001 0.000 8.7e+34 0.00

Knol (2015) | 0.000 0.000 4.6e+34 0.00

Lepoutre (2015)* | 0.000 0.000 1.2e+34 0.00

Miller (2011) | 0.568 0.183 1.762 0.17

Moore (2014) | 0.001 0.000 9.0e+34 0.00

Munoz-Almagro (2011) | 0.000 0.000 4.5e+34 0.00

Parra (2013) | 2.381 0.595 9.520 0.15

Perez-Trallero (2009 | 0.001 0.000 1.3e+35 0.00

Picazo (2011) | 0.909 0.293 2.819 0.17

Pilishvili (2010) | 0.578 0.186 1.792 0.17

Rendi-Wagner (2009) | 0.001 0.000 1.6e+35 0.00

Rodriguez (2011) | 0.820 0.264 2.542 0.17

Salleras (2009) | 0.833 0.208 3.332 0.15

Schutze (2004) | 0.001 0.000 7.8e+34 0.00

Sharma (2013)* | 2.128 0.300 15.105 0.12

Steens (2013) | 0.000 0.000 3.5e+34 0.00

Suga (2015) | 0.649 0.162 2.596 0.15

Varon (2015) | 0.258 0.036 1.830 0.12

Vila-Corcoles (2013) | 1.538 0.217 10.922 0.12

Weatherholtz (2010) | 0.000 0.000 5.1e+34 0.00

Williams (2011) | 0.000 0.000 2.9e+34 0.00

van der Linden (2015 | 0.185 0.026 1.310 0.12

Sub-total |

D+L pooled ES | 1.177 0.860 1.610 2.49

I-V pooled ES | 1.177 0.860 1.610

---------------------+---------------------------------------------------

8

Aguiar (2008) | 1.111 0.157 7.888 0.12

Aristegui (2007) | 0.001 0.000 7.6e+34 0.00

Barricarte (2007) | 0.001 0.000 6.9e+34 0.00

Bettinger (2010) | 0.000 0.000 2.8e+34 0.00

Black (2007) | 1.190 0.168 8.452 0.12

Bruce (2015) | 0.794 0.112 5.634 0.12

Byington (2005) | 1.905 0.476 7.616 0.15

Calbo (2006) | 0.001 0.000 9.1e+34 0.00

Ceyhan (2011) | 1.370 0.343 5.477 0.15

Chiba (2014) | 0.000 0.000 1.9e+34 0.00

Croney (2013) | 0.000 0.000 3.7e+34 0.00

De Wals (2012) | 0.000 0.000 5.2e+34 0.00

Guevara (2014) | 0.000 0.000 5.5e+34 0.00

Hsu (2010) | 0.000 0.000 4.5e+34 0.00

Ishiwada (2014) | 0.002 0.000 1.8e+35 0.00

Kaplan (2013) | 0.493 0.159 1.527 0.17

Kellner (2009) | 1.493 0.210 10.596 0.12

Knol (2015) | 2.344 0.756 7.267 0.17

Lepoutre (2015)* | 0.000 0.000 1.2e+34 0.00

Miller (2011) | 2.841 1.713 4.712 0.20

Moore (2014) | 1.538 0.217 10.922 0.12

Munoz-Almagro (2011) | 0.000 0.000 4.5e+34 0.00

Parra (2013) | 2.381 0.595 9.520 0.15

Perez-Trallero (2009 | 0.001 0.000 1.3e+35 0.00

Picazo (2011) | 0.606 0.152 2.423 0.15

Pilishvili (2010) | 0.000 0.000 1.1e+34 0.00

Rendi-Wagner (2009) | 0.001 0.000 1.6e+35 0.00

Rodriguez (2011) | 0.820 0.264 2.542 0.17

Salleras (2009) | 0.417 0.059 2.958 0.12

Schutze (2004) | 0.001 0.000 7.8e+34 0.00

Sharma (2013)* | 0.001 0.000 1.2e+35 0.00

Steens (2013) | 0.000 0.000 3.5e+34 0.00

Suga (2015) | 0.000 0.000 1.9e+34 0.00

Varon (2015) | 0.258 0.036 1.830 0.12

Vila-Corcoles (2013) | 0.001 0.000 9.0e+34 0.00

Weatherholtz (2010) | 0.870 0.122 6.173 0.12

Williams (2011) | 0.000 0.000 2.9e+34 0.00

van der Linden (2015 | 0.923 0.384 2.216 0.18

Sub-total |

D+L pooled ES | 1.432 1.069 1.918 2.42

I-V pooled ES | 1.432 1.069 1.918

---------------------+---------------------------------------------------

23A

Aguiar (2008) | 0.001 0.000 6.5e+34 0.00

Aristegui (2007) | 0.001 0.000 7.6e+34 0.00

Barricarte (2007) | 0.001 0.000 6.9e+34 0.00

Bettinger (2010) | 0.000 0.000 2.8e+34 0.00

Black (2007) | 0.001 0.000 7.0e+34 0.00

Bruce (2015) | 0.000 0.000 4.6e+34 0.00

Byington (2005) | 0.000 0.000 5.6e+34 0.00

Calbo (2006) | 0.001 0.000 9.1e+34 0.00

Ceyhan (2011) | 0.685 0.096 4.863 0.12

Chiba (2014) | 3.642 2.017 6.577 0.20

Croney (2013) | 2.548 0.956 6.788 0.18

De Wals (2012) | 1.770 0.443 7.077 0.15

Guevara (2014) | 0.000 0.000 5.5e+34 0.00

Hsu (2010) | 0.000 0.000 4.5e+34 0.00

Ishiwada (2014) | 0.002 0.000 1.8e+35 0.00

Kaplan (2013) | 1.642 0.883 3.052 0.20

Kellner (2009) | 0.001 0.000 8.7e+34 0.00

Knol (2015) | 0.781 0.110 5.546 0.12

Lepoutre (2015)* | 0.000 0.000 1.2e+34 0.00

Miller (2011) | 0.758 0.284 2.019 0.18

Moore (2014) | 0.001 0.000 9.0e+34 0.00

Munoz-Almagro (2011) | 0.000 0.000 4.5e+34 0.00

Parra (2013) | 0.001 0.000 7.0e+34 0.00

Perez-Trallero (2009 | 0.001 0.000 1.3e+35 0.00

Picazo (2011) | 0.000 0.000 1.8e+34 0.00

Pilishvili (2010) | 1.734 0.902 3.333 0.20

Rendi-Wagner (2009) | 2.778 0.391 19.720 0.12

Rodriguez (2011) | 0.546 0.137 2.185 0.15

Salleras (2009) | 0.000 0.000 2.4e+34 0.00

Schutze (2004) | 0.001 0.000 7.8e+34 0.00

Sharma (2013)* | 0.001 0.000 1.2e+35 0.00

Steens (2013) | 0.000 0.000 3.5e+34 0.00

Suga (2015) | 0.325 0.046 2.305 0.12

Varon (2015) | 0.515 0.129 2.061 0.15

Vila-Corcoles (2013) | 0.001 0.000 9.0e+34 0.00

Weatherholtz (2010) | 0.000 0.000 5.1e+34 0.00

Williams (2011) | 0.000 0.000 2.9e+34 0.00

van der Linden (2015 | 0.554 0.179 1.716 0.17

Sub-total |

D+L pooled ES | 1.563 1.188 2.056 2.03

I-V pooled ES | 1.563 1.188 2.056

---------------------+---------------------------------------------------

9N

Aguiar (2008) | 0.001 0.000 6.5e+34 0.00

Aristegui (2007) | 0.001 0.000 7.6e+34 0.00

Barricarte (2007) | 0.001 0.000 6.9e+34 0.00

Bettinger (2010) | 0.472 0.066 3.349 0.12

Black (2007) | 0.001 0.000 7.0e+34 0.00

Bruce (2015) | 1.587 0.397 6.347 0.15

Byington (2005) | 0.000 0.000 5.6e+34 0.00

Calbo (2006) | 0.001 0.000 9.1e+34 0.00

Ceyhan (2011) | 0.000 0.000 4.0e+34 0.00

Chiba (2014) | 0.000 0.000 1.9e+34 0.00

Croney (2013) | 0.637 0.090 4.522 0.12

De Wals (2012) | 0.000 0.000 5.2e+34 0.00

Guevara (2014) | 0.000 0.000 5.5e+34 0.00

Hsu (2010) | 0.000 0.000 4.5e+34 0.00

Ishiwada (2014) | 0.002 0.000 1.8e+35 0.00

Kaplan (2013) | 0.657 0.247 1.750 0.18

Kellner (2009) | 0.001 0.000 8.7e+34 0.00

Knol (2015) | 0.000 0.000 4.6e+34 0.00

Lepoutre (2015)* | 0.000 0.000 1.2e+34 0.00

Miller (2011) | 0.568 0.183 1.762 0.17

Moore (2014) | 3.077 0.770 12.303 0.15

Munoz-Almagro (2011) | 1.538 0.385 6.152 0.15

Parra (2013) | 0.001 0.000 7.0e+34 0.00

Perez-Trallero (2009 | 0.001 0.000 1.3e+35 0.00

Picazo (2011) | 0.000 0.000 1.8e+34 0.00

Pilishvili (2010) | 0.578 0.186 1.792 0.17

Rendi-Wagner (2009) | 0.001 0.000 1.6e+35 0.00

Rodriguez (2011) | 0.000 0.000 1.6e+34 0.00

Salleras (2009) | 0.833 0.208 3.332 0.15

Schutze (2004) | 0.001 0.000 7.8e+34 0.00

Sharma (2013)* | 0.001 0.000 1.2e+35 0.00

Steens (2013) | 0.000 0.000 3.5e+34 0.00

Suga (2015) | 0.000 0.000 1.9e+34 0.00

Varon (2015) | 0.258 0.036 1.830 0.12

Vila-Corcoles (2013) | 1.538 0.217 10.922 0.12

Weatherholtz (2010) | 0.000 0.000 5.1e+34 0.00

Williams (2011) | 0.000 0.000 2.9e+34 0.00

van der Linden (2015 | 1.107 0.497 2.464 0.19

Sub-total |

D+L pooled ES | 0.899 0.621 1.302 1.77

I-V pooled ES | 0.899 0.621 1.302

---------------------+---------------------------------------------------

38

Aguiar (2008) | 0.001 0.000 6.5e+34 0.00

Aristegui (2007) | 0.001 0.000 7.6e+34 0.00

Barricarte (2007) | 0.001 0.000 6.9e+34 0.00

Bettinger (2010) | 2.830 1.271 6.300 0.19

Black (2007) | 8.333 3.973 17.480 0.19

Bruce (2015) | 0.000 0.000 4.6e+34 0.00

Byington (2005) | 0.000 0.000 5.6e+34 0.00

Calbo (2006) | 3.125 0.782 12.495 0.15

Ceyhan (2011) | 0.000 0.000 4.0e+34 0.00

Chiba (2014) | 1.987 0.893 4.422 0.19

Croney (2013) | 0.000 0.000 3.7e+34 0.00

De Wals (2012) | 1.770 0.443 7.077 0.15

Guevara (2014) | 0.000 0.000 5.5e+34 0.00

Hsu (2010) | 0.000 0.000 4.5e+34 0.00

Ishiwada (2014) | 3.030 0.427 21.513 0.12

Kaplan (2013) | 0.985 0.443 2.193 0.19

Kellner (2009) | 1.493 0.210 10.596 0.12

Knol (2015) | 0.781 0.110 5.546 0.12

Lepoutre (2015)* | 0.000 0.000 1.2e+34 0.00

Miller (2011) | 1.136 0.511 2.529 0.19

Moore (2014) | 1.538 0.217 10.922 0.12

Munoz-Almagro (2011) | 2.308 0.744 7.155 0.17

Parra (2013) | 0.001 0.000 7.0e+34 0.00

Perez-Trallero (2009 | 0.001 0.000 1.3e+35 0.00

Picazo (2011) | 0.000 0.000 1.8e+34 0.00

Pilishvili (2010) | 2.697 1.598 4.555 0.20

Rendi-Wagner (2009) | 0.001 0.000 1.6e+35 0.00

Rodriguez (2011) | 0.000 0.000 1.6e+34 0.00

Salleras (2009) | 0.833 0.208 3.332 0.15

Schutze (2004) | 0.001 0.000 7.8e+34 0.00

Sharma (2013)* | 4.255 1.064 17.015 0.15

Steens (2013) | 1.212 0.303 4.847 0.15

Suga (2015) | 1.299 0.487 3.460 0.18

Varon (2015) | 0.000 0.000 1.5e+34 0.00

Vila-Corcoles (2013) | 0.001 0.000 9.0e+34 0.00

Weatherholtz (2010) | 2.609 0.841 8.089 0.17

Williams (2011) | 0.000 0.000 2.9e+34 0.00

van der Linden (2015 | 2.768 1.668 4.591 0.20

Sub-total |

D+L pooled ES | 2.285 1.845 2.830 3.07

I-V pooled ES | 2.285 1.845 2.830

---------------------+---------------------------------------------------

15BC

Aguiar (2008) | 1.111 0.157 7.888 0.12

Aristegui (2007) | 0.001 0.000 7.6e+34 0.00

Barricarte (2007) | 3.529 1.138 10.943 0.17

Bettinger (2010) | 4.245 2.209 8.159 0.20

Black (2007) | 8.333 3.973 17.480 0.19

Bruce (2015) | 1.587 0.397 6.347 0.15

Byington (2005) | 0.000 0.000 5.6e+34 0.00

Calbo (2006) | 1.563 0.220 11.093 0.12

Ceyhan (2011) | 1.370 0.343 5.477 0.15

Chiba (2014) | 8.278 5.594 12.251 0.21

Croney (2013) | 0.000 0.000 3.7e+34 0.00

De Wals (2012) | 4.425 1.842 10.631 0.18

Guevara (2014) | 0.943 0.133 6.697 0.12

Hsu (2010) | 0.000 0.000 4.5e+34 0.00

Ishiwada (2014) | 3.030 0.427 21.513 0.12

Kaplan (2013) | 4.598 3.175 6.659 0.21

Kellner (2009) | 0.001 0.000 8.7e+34 0.00

Knol (2015) | 3.125 1.173 8.326 0.18

Lepoutre (2015)* | 3.106 1.872 5.151 0.20

Miller (2011) | 4.924 3.353 7.232 0.21

Moore (2014) | 7.692 3.202 18.481 0.18

Munoz-Almagro (2011) | 0.769 0.108 5.461 0.12

Parra (2013) | 5.952 2.478 14.301 0.18

Perez-Trallero (2009 | 0.001 0.000 1.3e+35 0.00

Picazo (2011) | 2.424 1.212 4.848 0.19

Pilishvili (2010) | 0.000 0.000 1.1e+34 0.00

Rendi-Wagner (2009) | 0.001 0.000 1.6e+35 0.00

Rodriguez (2011) | 4.372 2.678 7.136 0.20

Salleras (2009) | 0.000 0.000 2.4e+34 0.00

Schutze (2004) | 0.001 0.000 7.8e+34 0.00

Sharma (2013)* | 0.001 0.000 1.2e+35 0.00

Steens (2013) | 0.000 0.000 3.5e+34 0.00

Suga (2015) | 6.818 4.445 10.457 0.21

Varon (2015) | 0.000 0.000 1.5e+34 0.00

Vila-Corcoles (2013) | 3.077 0.770 12.303 0.15

Weatherholtz (2010) | 0.000 0.000 5.1e+34 0.00

Williams (2011) | 0.000 0.000 2.9e+34 0.00

van der Linden (2015 | 5.720 4.022 8.133 0.21

Sub-total |

D+L pooled ES | 4.805 4.168 5.539 3.94

I-V pooled ES | 4.874 4.270 5.564

---------------------+---------------------------------------------------

Study | ES [95% Conf. Interval] % Weight

---------------------+---------------------------------------------------

33F Europe

Aristegui (2007) | 0.001 0.000 7.6e+34 0.00

Barricarte (2007) | 0.001 0.000 6.9e+34 0.00

Calbo (2006) | 0.001 0.000 9.1e+34 0.00

Guevara (2014) | 1.887 0.472 7.544 0.15

Munoz-Almagro (2011) | 0.000 0.000 4.5e+34 0.00

Perez-Trallero (2009 | 0.001 0.000 1.3e+35 0.00

Picazo (2011) | 0.303 0.043 2.151 0.12

Rodriguez (2011) | 0.000 0.000 1.6e+34 0.00

Salleras (2009) | 2.083 0.867 5.005 0.18

Vila-Corcoles (2013) | 0.001 0.000 9.0e+34 0.00

Lepoutre (2015)* | 0.000 0.000 1.2e+34 0.00

Varon (2015) | 1.289 0.536 3.096 0.18

van der Linden (2015 | 1.292 0.616 2.709 0.19

Knol (2015) | 8.594 4.759 15.518 0.20

Steens (2013) | 5.455 2.838 10.483 0.20

Miller (2011) | 3.598 2.295 5.642 0.20

Moore (2014) | 13.846 7.204 26.611 0.20

Rendi-Wagner (2009) | 0.001 0.000 1.6e+35 0.00

Aguiar (2008) | 1.111 0.157 7.888 0.12

Ceyhan (2011) | 0.000 0.000 4.0e+34 0.00

Sub-total |

D+L pooled ES | 2.961 1.731 5.065 1.73

---------------------+---------------------------------------------------

15C Europe

Aristegui (2007) | 0.001 0.000 7.6e+34 0.00

Barricarte (2007) | 2.353 0.588 9.408 0.15

Calbo (2006) | 1.563 0.220 11.093 0.12

Guevara (2014) | 0.000 0.000 5.5e+34 0.00

Munoz-Almagro (2011) | 0.000 0.000 4.5e+34 0.00

Perez-Trallero (2009 | 0.001 0.000 1.3e+35 0.00

Picazo (2011) | 0.606 0.152 2.423 0.15

Rodriguez (2011) | 1.093 0.410 2.912 0.18

Salleras (2009) | 0.000 0.000 2.4e+34 0.00

Vila-Corcoles (2013) | 3.077 0.770 12.303 0.15

Lepoutre (2015)* | 1.242 0.558 2.765 0.19

Varon (2015) | 0.000 0.000 1.5e+34 0.00

van der Linden (2015 | 2.952 1.808 4.819 0.20

Knol (2015) | 0.781 0.110 5.546 0.12

Steens (2013) | 0.000 0.000 3.5e+34 0.00

Miller (2011) | 2.462 1.430 4.240 0.20

Moore (2014) | 6.154 2.310 16.397 0.18

Rendi-Wagner (2009) | 0.001 0.000 1.6e+35 0.00

Aguiar (2008) | 0.001 0.000 6.5e+34 0.00

Ceyhan (2011) | 1.370 0.343 5.477 0.15

Sub-total |

D+L pooled ES | 2.197 1.678 2.875 1.78

---------------------+---------------------------------------------------

pcv7 Europe

Aristegui (2007) | 18.182 10.768 30.700 0.20

Barricarte (2007) | 41.176 29.564 57.350 0.21

Calbo (2006) | 28.125 17.720 44.640 0.20

Guevara (2014) | 9.434 5.076 17.534 0.20

Munoz-Almagro (2011) | 6.923 3.602 13.306 0.20

Perez-Trallero (2009 | 33.333 20.095 55.292 0.20

Picazo (2011) | 4.545 2.740 7.540 0.20

Rodriguez (2011) | 4.918 3.099 7.806 0.20

Salleras (2009) | 31.667 25.291 39.650 0.21

Vila-Corcoles (2013) | 35.385 23.514 53.248 0.21

Lepoutre (2015)* | 11.180 8.563 14.598 0.21

Varon (2015) | 6.701 4.563 9.842 0.21

van der Linden (2015 | 23.247 19.523 27.682 0.21

Knol (2015) | 3.906 1.626 9.385 0.18

Steens (2013) | 23.030 16.758 31.651 0.21

Miller (2011) | 7.955 5.879 10.764 0.21

Moore (2014) | 9.231 4.147 20.547 0.19

Rendi-Wagner (2009) | 75.000 51.433 109.365 0.21

Aguiar (2008) | 30.000 20.573 43.746 0.21

Ceyhan (2011) | 57.534 46.457 71.253 0.21

Sub-total |

D+L pooled ES | 16.838 11.850 23.926 4.07

---------------------+---------------------------------------------------

19A Europe

Aristegui (2007) | 0.001 0.000 7.6e+34 0.00

Barricarte (2007) | 22.353 14.258 35.044 0.20

Calbo (2006) | 0.001 0.000 9.1e+34 0.00

Guevara (2014) | 32.075 22.919 44.891 0.21

Munoz-Almagro (2011) | 20.000 13.617 29.374 0.21

Perez-Trallero (2009 | 13.333 5.990 29.679 0.19

Picazo (2011) | 18.788 14.648 24.098 0.21

Rodriguez (2011) | 22.404 18.044 27.819 0.21

Salleras (2009) | 22.083 16.871 28.906 0.21

Vila-Corcoles (2013) | 10.769 5.134 22.590 0.19

Lepoutre (2015)* | 27.536 23.232 32.637 0.21

Varon (2015) | 32.990 27.742 39.230 0.21

van der Linden (2015 | 9.225 6.992 12.172 0.21

Knol (2015) | 22.656 15.744 32.603 0.21

Steens (2013) | 10.303 6.405 16.574 0.20

Miller (2011) | 18.939 15.568 23.040 0.21

Moore (2014) | 13.846 7.204 26.611 0.20

Rendi-Wagner (2009) | 0.001 0.000 1.6e+35 0.00

Aguiar (2008) | 24.444 16.095 37.124 0.21

Ceyhan (2011) | 6.164 3.207 11.848 0.20

Sub-total |

D+L pooled ES | 18.737 15.447 22.727 3.48

---------------------+---------------------------------------------------

15A Europe

Aristegui (2007) | 0.001 0.000 7.6e+34 0.00

Barricarte (2007) | 0.001 0.000 6.9e+34 0.00

Calbo (2006) | 0.001 0.000 9.1e+34 0.00

Guevara (2014) | 0.943 0.133 6.697 0.12

Munoz-Almagro (2011) | 0.000 0.000 4.5e+34 0.00

Perez-Trallero (2009 | 0.001 0.000 1.3e+35 0.00

Picazo (2011) | 0.606 0.152 2.423 0.15

Rodriguez (2011) | 0.820 0.264 2.542 0.17

Salleras (2009) | 0.417 0.059 2.958 0.12

Vila-Corcoles (2013) | 0.001 0.000 9.0e+34 0.00

Lepoutre (2015)* | 1.863 0.970 3.581 0.20

Varon (2015) | 1.546 0.695 3.442 0.19

van der Linden (2015 | 0.369 0.092 1.475 0.15

Knol (2015) | 0.781 0.110 5.546 0.12

Steens (2013) | 0.000 0.000 3.5e+34 0.00

Miller (2011) | 0.947 0.394 2.275 0.18

Moore (2014) | 1.538 0.217 10.922 0.12

Rendi-Wagner (2009) | 0.001 0.000 1.6e+35 0.00

Aguiar (2008) | 0.001 0.000 6.5e+34 0.00

Ceyhan (2011) | 0.000 0.000 4.0e+34 0.00

Sub-total |

D+L pooled ES | 1.122 0.789 1.595 1.50

---------------------+---------------------------------------------------

23B Europe

Aristegui (2007) | 0.001 0.000 7.6e+34 0.00

Barricarte (2007) | 2.353 0.588 9.408 0.15

Calbo (2006) | 0.001 0.000 9.1e+34 0.00

Guevara (2014) | 0.000 0.000 5.5e+34 0.00

Munoz-Almagro (2011) | 2.308 0.744 7.155 0.17

Perez-Trallero (2009 | 0.001 0.000 1.3e+35 0.00

Picazo (2011) | 0.909 0.293 2.819 0.17

Rodriguez (2011) | 2.186 1.093 4.371 0.19

Salleras (2009) | 2.500 1.123 5.565 0.19

Vila-Corcoles (2013) | 0.001 0.000 9.0e+34 0.00

Lepoutre (2015)* | 0.000 0.000 1.2e+34 0.00

Varon (2015) | 0.773 0.249 2.397 0.17

van der Linden (2015 | 0.554 0.179 1.716 0.17

Knol (2015) | 0.781 0.110 5.546 0.12

Steens (2013) | 1.212 0.303 4.847 0.15

Miller (2011) | 1.326 0.632 2.781 0.19

Moore (2014) | 1.538 0.217 10.922 0.12

Rendi-Wagner (2009) | 0.001 0.000 1.6e+35 0.00

Aguiar (2008) | 1.111 0.157 7.888 0.12

Ceyhan (2011) | 0.000 0.000 4.0e+34 0.00

Sub-total |

D+L pooled ES | 1.483 1.088 2.021 1.89

---------------------+---------------------------------------------------

23A Europe

Aristegui (2007) | 0.001 0.000 7.6e+34 0.00

Barricarte (2007) | 0.001 0.000 6.9e+34 0.00

Calbo (2006) | 0.001 0.000 9.1e+34 0.00

Guevara (2014) | 0.000 0.000 5.5e+34 0.00

Munoz-Almagro (2011) | 0.000 0.000 4.5e+34 0.00

Perez-Trallero (2009 | 0.001 0.000 1.3e+35 0.00

Picazo (2011) | 0.000 0.000 1.8e+34 0.00

Rodriguez (2011) | 0.546 0.137 2.185 0.15

Salleras (2009) | 0.000 0.000 2.4e+34 0.00

Vila-Corcoles (2013) | 0.001 0.000 9.0e+34 0.00

Lepoutre (2015)* | 0.000 0.000 1.2e+34 0.00

Varon (2015) | 0.515 0.129 2.061 0.15

van der Linden (2015 | 0.554 0.179 1.716 0.17

Knol (2015) | 0.781 0.110 5.546 0.12

Steens (2013) | 0.000 0.000 3.5e+34 0.00

Miller (2011) | 0.758 0.284 2.019 0.18

Moore (2014) | 0.001 0.000 9.0e+34 0.00

Rendi-Wagner (2009) | 2.778 0.391 19.720 0.12

Aguiar (2008) | 0.001 0.000 6.5e+34 0.00

Ceyhan (2011) | 0.685 0.096 4.863 0.12

Sub-total |

D+L pooled ES | 0.696 0.412 1.175 0.99

---------------------+---------------------------------------------------

non-pcv Europe

Aristegui (2007) | 11.688 6.082 22.464 0.20

Barricarte (2007) | 12.941 7.167 23.368 0.20

Calbo (2006) | 21.875 12.955 36.936 0.20

Guevara (2014) | 21.698 14.419 32.652 0.21

Munoz-Almagro (2011) | 20.000 13.617 29.374 0.21

Perez-Trallero (2009 | 13.333 5.990 29.679 0.19

Picazo (2011) | 20.909 16.514 26.473 0.21

Rodriguez (2011) | 25.137 20.491 30.836 0.21

Salleras (2009) | 22.083 16.871 28.906 0.21

Vila-Corcoles (2013) | 15.385 8.278 28.593 0.20

Lepoutre (2015)* | 28.571 24.181 33.759 0.21

Varon (2015) | 32.474 27.271 38.670 0.21

van der Linden (2015 | 31.919 27.500 37.048 0.21

Knol (2015) | 47.656 37.079 61.250 0.21

Steens (2013) | 30.909 23.490 40.671 0.21

Miller (2011) | 30.303 25.953 35.382 0.21

Moore (2014) | 50.769 36.093 71.413 0.21

Rendi-Wagner (2009) | 2.778 0.391 19.720 0.12

Aguiar (2008) | 14.444 8.387 24.876 0.20

Ceyhan (2011) | 22.603 16.069 31.794 0.21

Sub-total |

D+L pooled ES | 25.012 21.729 28.791 4.02

---------------------+---------------------------------------------------

22F Europe

Aristegui (2007) | 0.001 0.000 7.6e+34 0.00

Barricarte (2007) | 0.001 0.000 6.9e+34 0.00

Calbo (2006) | 0.001 0.000 9.1e+34 0.00

Guevara (2014) | 1.887 0.472 7.544 0.15

Munoz-Almagro (2011) | 0.769 0.108 5.461 0.12

Perez-Trallero (2009 | 0.001 0.000 1.3e+35 0.00

Picazo (2011) | 0.606 0.152 2.423 0.15

Rodriguez (2011) | 1.093 0.410 2.912 0.18

Salleras (2009) | 0.417 0.059 2.958 0.12

Vila-Corcoles (2013) | 1.538 0.217 10.922 0.12

Lepoutre (2015)* | 1.863 0.970 3.581 0.20

Varon (2015) | 1.546 0.695 3.442 0.19

van der Linden (2015 | 1.845 0.993 3.429 0.20

Knol (2015) | 3.906 1.626 9.385 0.18

Steens (2013) | 5.455 2.838 10.483 0.20

Miller (2011) | 5.682 3.973 8.126 0.21

Moore (2014) | 4.615 1.489 14.311 0.17

Rendi-Wagner (2009) | 0.001 0.000 1.6e+35 0.00

Aguiar (2008) | 2.222 0.556 8.886 0.15

Ceyhan (2011) | 0.000 0.000 4.0e+34 0.00

Sub-total |

D+L pooled ES | 2.234 1.501 3.326 2.31

---------------------+---------------------------------------------------

35B Europe

Aristegui (2007) | 0.001 0.000 7.6e+34 0.00

Barricarte (2007) | 0.001 0.000 6.9e+34 0.00

Calbo (2006) | 0.001 0.000 9.1e+34 0.00

Guevara (2014) | 0.000 0.000 5.5e+34 0.00

Munoz-Almagro (2011) | 0.000 0.000 4.5e+34 0.00

Perez-Trallero (2009 | 0.001 0.000 1.3e+35 0.00

Picazo (2011) | 0.909 0.293 2.819 0.17

Rodriguez (2011) | 0.820 0.264 2.542 0.17

Salleras (2009) | 0.833 0.208 3.332 0.15

Vila-Corcoles (2013) | 1.538 0.217 10.922 0.12

Lepoutre (2015)* | 0.000 0.000 1.2e+34 0.00

Varon (2015) | 0.258 0.036 1.830 0.12

van der Linden (2015 | 0.185 0.026 1.310 0.12

Knol (2015) | 0.000 0.000 4.6e+34 0.00

Steens (2013) | 0.000 0.000 3.5e+34 0.00

Miller (2011) | 0.568 0.183 1.762 0.17

Moore (2014) | 0.001 0.000 9.0e+34 0.00

Rendi-Wagner (2009) | 0.001 0.000 1.6e+35 0.00

Aguiar (2008) | 0.001 0.000 6.5e+34 0.00

Ceyhan (2011) | 0.000 0.000 4.0e+34 0.00

Sub-total |

D+L pooled ES | 0.670 0.397 1.131 1.00

---------------------+---------------------------------------------------

pcv10-pcv7 Europe

Aristegui (2007) | 10.390 5.196 20.775 0.19

Barricarte (2007) | 7.059 3.171 15.712 0.19

Calbo (2006) | 20.313 11.794 34.982 0.20

Guevara (2014) | 30.189 21.349 42.689 0.21

Munoz-Almagro (2011) | 47.692 37.183 61.172 0.21

Perez-Trallero (2009 | 35.556 21.782 58.038 0.20

Picazo (2011) | 50.000 42.924 58.242 0.21

Rodriguez (2011) | 42.077 35.929 49.276 0.21

Salleras (2009) | 15.000 10.820 20.795 0.21

Vila-Corcoles (2013) | 38.462 25.989 56.921 0.21

Lepoutre (2015)* | 27.329 23.043 32.413 0.21

Varon (2015) | 22.423 18.173 27.666 0.21

van der Linden (2015 | 25.277 21.379 29.884 0.21

Knol (2015) | 21.094 14.466 30.759 0.21

Steens (2013) | 23.636 17.269 32.351 0.21

Miller (2011) | 33.523 28.931 38.844 0.21

Moore (2014) | 23.077 13.912 38.279 0.20

Rendi-Wagner (2009) | 11.111 4.170 29.605 0.18

Aguiar (2008) | 25.556 16.982 38.457 0.21

Ceyhan (2011) | 7.534 4.172 13.605 0.20

Sub-total |

D+L pooled ES | 24.895 20.692 29.950 4.08

---------------------+---------------------------------------------------

6C Europe

Aristegui (2007) | 0.001 0.000 7.6e+34 0.00

Barricarte (2007) | 0.001 0.000 6.9e+34 0.00

Calbo (2006) | 0.001 0.000 9.1e+34 0.00

Guevara (2014) | 1.887 0.472 7.544 0.15

Munoz-Almagro (2011) | 0.000 0.000 4.5e+34 0.00

Perez-Trallero (2009 | 0.001 0.000 1.3e+35 0.00

Picazo (2011) | 0.606 0.152 2.423 0.15

Rodriguez (2011) | 0.000 0.000 1.6e+34 0.00

Salleras (2009) | 0.000 0.000 2.4e+34 0.00

Vila-Corcoles (2013) | 0.001 0.000 9.0e+34 0.00

Lepoutre (2015)* | 1.242 0.558 2.765 0.19

Varon (2015) | 1.031 0.387 2.747 0.18

van der Linden (2015 | 0.554 0.179 1.716 0.17

Knol (2015) | 0.000 0.000 4.6e+34 0.00

Steens (2013) | 1.212 0.303 4.847 0.15

Miller (2011) | 0.000 0.000 1.1e+34 0.00

Moore (2014) | 3.077 0.770 12.303 0.15

Rendi-Wagner (2009) | 0.001 0.000 1.6e+35 0.00

Aguiar (2008) | 0.001 0.000 6.5e+34 0.00

Ceyhan (2011) | 0.000 0.000 4.0e+34 0.00

Sub-total |

D+L pooled ES | 1.126 0.734 1.727 1.13

---------------------+---------------------------------------------------

12F Europe

Aristegui (2007) | 0.001 0.000 7.6e+34 0.00

Barricarte (2007) | 0.001 0.000 6.9e+34 0.00

Calbo (2006) | 0.001 0.000 9.1e+34 0.00

Guevara (2014) | 0.943 0.133 6.697 0.12

Munoz-Almagro (2011) | 0.769 0.108 5.461 0.12

Perez-Trallero (2009 | 0.001 0.000 1.3e+35 0.00

Picazo (2011) | 1.515 0.631 3.640 0.18

Rodriguez (2011) | 2.186 1.093 4.371 0.19

Salleras (2009) | 2.917 1.390 6.118 0.19

Vila-Corcoles (2013) | 3.077 0.770 12.303 0.15

Lepoutre (2015)* | 1.863 0.970 3.581 0.20

Varon (2015) | 2.320 1.207 4.458 0.20

van der Linden (2015 | 1.292 0.616 2.709 0.19

Knol (2015) | 1.563 0.391 6.248 0.15

Steens (2013) | 0.000 0.000 3.5e+34 0.00

Miller (2011) | 1.515 0.758 3.030 0.19

Moore (2014) | 3.077 0.770 12.303 0.15

Rendi-Wagner (2009) | 0.001 0.000 1.6e+35 0.00

Aguiar (2008) | 0.001 0.000 6.5e+34 0.00

Ceyhan (2011) | 0.000 0.000 4.0e+34 0.00

Sub-total |

D+L pooled ES | 1.900 1.478 2.442 2.02

---------------------+---------------------------------------------------

6A Europe

Aristegui (2007) | 0.001 0.000 7.6e+34 0.00

Barricarte (2007) | 7.059 3.171 15.712 0.19

Calbo (2006) | 9.375 4.212 20.868 0.19

Guevara (2014) | 1.887 0.472 7.544 0.15

Munoz-Almagro (2011) | 2.308 0.744 7.155 0.17

Perez-Trallero (2009 | 4.444 1.112 17.771 0.15

Picazo (2011) | 1.212 0.455 3.230 0.18

Rodriguez (2011) | 2.186 1.093 4.371 0.19

Salleras (2009) | 7.083 4.403 11.394 0.20

Vila-Corcoles (2013) | 0.001 0.000 9.0e+34 0.00

Lepoutre (2015)* | 1.449 0.691 3.040 0.19

Varon (2015) | 0.773 0.249 2.397 0.17

van der Linden (2015 | 4.244 2.820 6.386 0.21

Knol (2015) | 1.563 0.391 6.248 0.15

Steens (2013) | 5.455 2.838 10.483 0.20

Miller (2011) | 0.000 0.000 1.1e+34 0.00

Moore (2014) | 1.538 0.217 10.922 0.12

Rendi-Wagner (2009) | 8.333 2.688 25.839 0.17

Aguiar (2008) | 2.222 0.556 8.886 0.15

Ceyhan (2011) | 2.055 0.663 6.371 0.17

Sub-total |

D+L pooled ES | 3.182 2.246 4.508 2.92

---------------------+---------------------------------------------------

11A Europe

Aristegui (2007) | 0.001 0.000 7.6e+34 0.00

Barricarte (2007) | 0.001 0.000 6.9e+34 0.00

Calbo (2006) | 0.001 0.000 9.1e+34 0.00

Guevara (2014) | 0.000 0.000 5.5e+34 0.00

Munoz-Almagro (2011) | 0.000 0.000 4.5e+34 0.00

Perez-Trallero (2009 | 0.001 0.000 1.3e+35 0.00

Picazo (2011) | 1.212 0.455 3.230 0.18

Rodriguez (2011) | 0.820 0.264 2.542 0.17

Salleras (2009) | 0.000 0.000 2.4e+34 0.00

Vila-Corcoles (2013) | 1.538 0.217 10.922 0.12

Lepoutre (2015)* | 0.000 0.000 1.2e+34 0.00

Varon (2015) | 0.258 0.036 1.830 0.12

van der Linden (2015 | 0.554 0.179 1.716 0.17

Knol (2015) | 2.344 0.756 7.267 0.17

Steens (2013) | 0.000 0.000 3.5e+34 0.00

Miller (2011) | 1.515 0.758 3.030 0.19

Moore (2014) | 0.001 0.000 9.0e+34 0.00

Rendi-Wagner (2009) | 0.001 0.000 1.6e+35 0.00

Aguiar (2008) | 0.001 0.000 6.5e+34 0.00

Ceyhan (2011) | 0.000 0.000 4.0e+34 0.00

Sub-total |

D+L pooled ES | 1.154 0.767 1.737 1.10

---------------------+---------------------------------------------------

3 Europe

Aristegui (2007) | 2.597 0.650 10.386 0.15

Barricarte (2007) | 5.882 2.448 14.133 0.18

Calbo (2006) | 0.001 0.000 9.1e+34 0.00

Guevara (2014) | 4.717 1.963 11.333 0.18

Munoz-Almagro (2011) | 3.077 1.155 8.198 0.18

Perez-Trallero (2009 | 0.001 0.000 1.3e+35 0.00

Picazo (2011) | 3.939 2.287 6.784 0.20

Rodriguez (2011) | 3.279 1.862 5.773 0.20

Salleras (2009) | 2.083 0.867 5.005 0.18

Vila-Corcoles (2013) | 0.001 0.000 9.0e+34 0.00

Lepoutre (2015)* | 3.934 2.509 6.167 0.20

Varon (2015) | 4.639 2.923 7.363 0.20

van der Linden (2015 | 6.089 4.328 8.564 0.21

Knol (2015) | 3.125 1.173 8.326 0.18

Steens (2013) | 6.667 3.692 12.038 0.20

Miller (2011) | 5.682 3.973 8.126 0.21

Moore (2014) | 0.001 0.000 9.0e+34 0.00

Rendi-Wagner (2009) | 2.778 0.391 19.720 0.12

Aguiar (2008) | 3.333 1.075 10.335 0.17

Ceyhan (2011) | 4.110 1.846 9.148 0.19

Sub-total |

D+L pooled ES | 4.646 3.999 5.397 2.94

---------------------+---------------------------------------------------

38 Europe

Aristegui (2007) | 0.001 0.000 7.6e+34 0.00

Barricarte (2007) | 0.001 0.000 6.9e+34 0.00

Calbo (2006) | 3.125 0.782 12.495 0.15

Guevara (2014) | 0.000 0.000 5.5e+34 0.00

Munoz-Almagro (2011) | 2.308 0.744 7.155 0.17

Perez-Trallero (2009 | 0.001 0.000 1.3e+35 0.00

Picazo (2011) | 0.000 0.000 1.8e+34 0.00

Rodriguez (2011) | 0.000 0.000 1.6e+34 0.00

Salleras (2009) | 0.833 0.208 3.332 0.15

Vila-Corcoles (2013) | 0.001 0.000 9.0e+34 0.00

Lepoutre (2015)* | 0.000 0.000 1.2e+34 0.00

Varon (2015) | 0.000 0.000 1.5e+34 0.00

van der Linden (2015 | 2.768 1.668 4.591 0.20

Knol (2015) | 0.781 0.110 5.546 0.12

Steens (2013) | 1.212 0.303 4.847 0.15

Miller (2011) | 1.136 0.511 2.529 0.19

Moore (2014) | 1.538 0.217 10.922 0.12

Rendi-Wagner (2009) | 0.001 0.000 1.6e+35 0.00

Aguiar (2008) | 0.001 0.000 6.5e+34 0.00

Ceyhan (2011) | 0.000 0.000 4.0e+34 0.00

Sub-total |

D+L pooled ES | 1.926 1.362 2.724 1.24

---------------------+---------------------------------------------------

24F Europe

Aristegui (2007) | 0.001 0.000 7.6e+34 0.00

Barricarte (2007) | 0.001 0.000 6.9e+34 0.00

Calbo (2006) | 0.001 0.000 9.1e+34 0.00

Guevara (2014) | 3.774 1.416 10.055 0.18

Munoz-Almagro (2011) | 4.615 2.073 10.273 0.19

Perez-Trallero (2009 | 0.001 0.000 1.3e+35 0.00

Picazo (2011) | 1.515 0.631 3.640 0.18

Rodriguez (2011) | 1.913 0.912 4.012 0.19

Salleras (2009) | 4.583 2.538 8.276 0.20

Vila-Corcoles (2013) | 0.001 0.000 9.0e+34 0.00

Lepoutre (2015)* | 4.555 2.999 6.918 0.21

Varon (2015) | 6.443 4.354 9.536 0.21

van der Linden (2015 | 3.875 2.526 5.943 0.21

Knol (2015) | 2.344 0.756 7.267 0.17

Steens (2013) | 0.000 0.000 3.5e+34 0.00

Miller (2011) | 0.568 0.183 1.762 0.17

Moore (2014) | 0.001 0.000 9.0e+34 0.00

Rendi-Wagner (2009) | 0.001 0.000 1.6e+35 0.00

Aguiar (2008) | 1.111 0.157 7.888 0.12

Ceyhan (2011) | 0.000 0.000 4.0e+34 0.00

Sub-total |

D+L pooled ES | 3.323 2.466 4.479 2.00

---------------------+---------------------------------------------------

15BC Europe

Aristegui (2007) | 0.001 0.000 7.6e+34 0.00

Barricarte (2007) | 3.529 1.138 10.943 0.17

Calbo (2006) | 1.563 0.220 11.093 0.12

Guevara (2014) | 0.943 0.133 6.697 0.12

Munoz-Almagro (2011) | 0.769 0.108 5.461 0.12

Perez-Trallero (2009 | 0.001 0.000 1.3e+35 0.00

Picazo (2011) | 2.424 1.212 4.848 0.19

Rodriguez (2011) | 4.372 2.678 7.136 0.20

Salleras (2009) | 0.000 0.000 2.4e+34 0.00

Vila-Corcoles (2013) | 3.077 0.770 12.303 0.15

Lepoutre (2015)* | 3.106 1.872 5.151 0.20

Varon (2015) | 0.000 0.000 1.5e+34 0.00

van der Linden (2015 | 5.720 4.022 8.133 0.21

Knol (2015) | 3.125 1.173 8.326 0.18

Steens (2013) | 0.000 0.000 3.5e+34 0.00

Miller (2011) | 4.924 3.353 7.232 0.21

Moore (2014) | 7.692 3.202 18.481 0.18

Rendi-Wagner (2009) | 0.001 0.000 1.6e+35 0.00

Aguiar (2008) | 1.111 0.157 7.888 0.12

Ceyhan (2011) | 1.370 0.343 5.477 0.15

Sub-total |

D+L pooled ES | 3.989 3.248 4.900 2.30

---------------------+---------------------------------------------------

pcv13 Europe

Aristegui (2007) | 88.312 69.629 112.007 0.21

Barricarte (2007) | 87.059 69.320 109.336 0.21

Calbo (2006) | 78.125 59.212 103.079 0.21

Guevara (2014) | 78.302 63.145 97.097 0.21

Munoz-Almagro (2011) | 80.000 66.012 96.952 0.21

Perez-Trallero (2009 | 86.667 63.321 118.619 0.21

Picazo (2011) | 79.091 70.055 89.293 0.21

Rodriguez (2011) | 74.863 66.504 84.274 0.21

Salleras (2009) | 77.917 67.512 89.924 0.21

Vila-Corcoles (2013) | 84.615 64.964 110.212 0.21

Lepoutre (2015)* | 71.429 64.275 79.378 0.21

Varon (2015) | 67.526 59.825 76.218 0.21

van der Linden (2015 | 68.081 61.477 75.395 0.21

Knol (2015) | 52.344 41.198 66.506 0.21

Steens (2013) | 69.091 57.504 83.013 0.21

Miller (2011) | 69.697 62.928 77.195 0.21

Moore (2014) | 49.231 34.815 69.616 0.21

Rendi-Wagner (2009) | 97.222 69.805 135.409 0.21

Aguiar (2008) | 85.556 68.429 106.968 0.21

Ceyhan (2011) | 77.397 64.365 93.068 0.21

Sub-total |

D+L pooled ES | 74.313 70.578 78.245 4.22

---------------------+---------------------------------------------------

15B Europe

Aristegui (2007) | 0.001 0.000 7.6e+34 0.00

Barricarte (2007) | 1.176 0.166 8.352 0.12

Calbo (2006) | 0.001 0.000 9.1e+34 0.00

Guevara (2014) | 0.943 0.133 6.697 0.12

Munoz-Almagro (2011) | 0.769 0.108 5.461 0.12

Perez-Trallero (2009 | 0.001 0.000 1.3e+35 0.00

Picazo (2011) | 1.818 0.817 4.047 0.19

Rodriguez (2011) | 3.279 1.862 5.773 0.20

Salleras (2009) | 0.000 0.000 2.4e+34 0.00

Vila-Corcoles (2013) | 0.001 0.000 9.0e+34 0.00

Lepoutre (2015)* | 1.863 0.970 3.581 0.20

Varon (2015) | 0.000 0.000 1.5e+34 0.00

van der Linden (2015 | 2.768 1.668 4.591 0.20

Knol (2015) | 2.344 0.756 7.267 0.17

Steens (2013) | 0.000 0.000 3.5e+34 0.00

Miller (2011) | 2.462 1.430 4.240 0.20

Moore (2014) | 1.538 0.217 10.922 0.12

Rendi-Wagner (2009) | 0.001 0.000 1.6e+35 0.00

Aguiar (2008) | 1.111 0.157 7.888 0.12

Ceyhan (2011) | 0.000 0.000 4.0e+34 0.00

Sub-total |

D+L pooled ES | 2.331 1.821 2.984 1.73

---------------------+---------------------------------------------------

9N Europe

Aristegui (2007) | 0.001 0.000 7.6e+34 0.00

Barricarte (2007) | 0.001 0.000 6.9e+34 0.00

Calbo (2006) | 0.001 0.000 9.1e+34 0.00

Guevara (2014) | 0.000 0.000 5.5e+34 0.00

Munoz-Almagro (2011) | 1.538 0.385 6.152 0.15

Perez-Trallero (2009 | 0.001 0.000 1.3e+35 0.00

Picazo (2011) | 0.000 0.000 1.8e+34 0.00

Rodriguez (2011) | 0.000 0.000 1.6e+34 0.00

Salleras (2009) | 0.833 0.208 3.332 0.15

Vila-Corcoles (2013) | 1.538 0.217 10.922 0.12

Lepoutre (2015)* | 0.000 0.000 1.2e+34 0.00

Varon (2015) | 0.258 0.036 1.830 0.12

van der Linden (2015 | 1.107 0.497 2.464 0.19

Knol (2015) | 0.000 0.000 4.6e+34 0.00

Steens (2013) | 0.000 0.000 3.5e+34 0.00

Miller (2011) | 0.568 0.183 1.762 0.17

Moore (2014) | 3.077 0.770 12.303 0.15

Rendi-Wagner (2009) | 0.001 0.000 1.6e+35 0.00

Aguiar (2008) | 0.001 0.000 6.5e+34 0.00

Ceyhan (2011) | 0.000 0.000 4.0e+34 0.00

Sub-total |

D+L pooled ES | 1.041 0.647 1.674 1.04

---------------------+---------------------------------------------------

10A Europe

Aristegui (2007) | 0.001 0.000 7.6e+34 0.00

Barricarte (2007) | 0.001 0.000 6.9e+34 0.00

Calbo (2006) | 0.001 0.000 9.1e+34 0.00

Guevara (2014) | 1.887 0.472 7.544 0.15

Munoz-Almagro (2011) | 2.308 0.744 7.155 0.17

Perez-Trallero (2009 | 0.001 0.000 1.3e+35 0.00

Picazo (2011) | 0.909 0.293 2.819 0.17

Rodriguez (2011) | 1.913 0.912 4.012 0.19

Salleras (2009) | 2.500 1.123 5.565 0.19

Vila-Corcoles (2013) | 1.538 0.217 10.922 0.12

Lepoutre (2015)* | 0.000 0.000 1.2e+34 0.00

Varon (2015) | 1.804 0.860 3.784 0.19

van der Linden (2015 | 4.797 3.266 7.045 0.21

Knol (2015) | 10.938 6.478 18.468 0.20

Steens (2013) | 2.424 0.910 6.459 0.18

Miller (2011) | 1.136 0.511 2.529 0.19

Moore (2014) | 0.001 0.000 9.0e+34 0.00

Rendi-Wagner (2009) | 0.001 0.000 1.6e+35 0.00

Aguiar (2008) | 2.222 0.556 8.886 0.15

Ceyhan (2011) | 0.000 0.000 4.0e+34 0.00

Sub-total |

D+L pooled ES | 2.488 1.610 3.846 2.09

---------------------+---------------------------------------------------

8 Europe

Aristegui (2007) | 0.001 0.000 7.6e+34 0.00

Barricarte (2007) | 0.001 0.000 6.9e+34 0.00

Calbo (2006) | 0.001 0.000 9.1e+34 0.00

Guevara (2014) | 0.000 0.000 5.5e+34 0.00

Munoz-Almagro (2011) | 0.000 0.000 4.5e+34 0.00

Perez-Trallero (2009 | 0.001 0.000 1.3e+35 0.00

Picazo (2011) | 0.606 0.152 2.423 0.15

Rodriguez (2011) | 0.820 0.264 2.542 0.17

Salleras (2009) | 0.417 0.059 2.958 0.12

Vila-Corcoles (2013) | 0.001 0.000 9.0e+34 0.00

Lepoutre (2015)* | 0.000 0.000 1.2e+34 0.00

Varon (2015) | 0.258 0.036 1.830 0.12

van der Linden (2015 | 0.923 0.384 2.216 0.18

Knol (2015) | 2.344 0.756 7.267 0.17

Steens (2013) | 0.000 0.000 3.5e+34 0.00

Miller (2011) | 2.841 1.713 4.712 0.20

Moore (2014) | 1.538 0.217 10.922 0.12

Rendi-Wagner (2009) | 0.001 0.000 1.6e+35 0.00

Aguiar (2008) | 1.111 0.157 7.888 0.12

Ceyhan (2011) | 1.370 0.343 5.477 0.15

Sub-total |

D+L pooled ES | 1.560 1.114 2.183 1.48

---------------------+---------------------------------------------------

15A Latin America

Parra (2013) | 2.381 0.595 9.520 0.15

Sub-total |

D+L pooled ES | 2.381 0.595 9.520 0.15

---------------------+---------------------------------------------------

12F Latin America

Parra (2013) | 0.001 0.000 7.0e+34 0.00

Sub-total |

D+L pooled ES | 0.001 0.000 7.0e+34 0.00

---------------------+---------------------------------------------------

35B Latin America

Parra (2013) | 2.381 0.595 9.520 0.15

Sub-total |

D+L pooled ES | 2.381 0.595 9.520 0.15

---------------------+---------------------------------------------------

15BC Latin America

Parra (2013) | 5.952 2.478 14.301 0.18

Sub-total |

D+L pooled ES | 5.952 2.478 14.301 0.18

---------------------+---------------------------------------------------

6A Latin America

Parra (2013) | 1.190 0.168 8.452 0.12

Sub-total |

D+L pooled ES | 1.190 0.168 8.452 0.12

---------------------+---------------------------------------------------

pcv7 Latin America

Parra (2013) | 38.095 26.940 53.870 0.21

Sub-total |

D+L pooled ES | 38.095 26.940 53.870 0.21

---------------------+---------------------------------------------------

22F Latin America

Parra (2013) | 1.190 0.168 8.452 0.12

Sub-total |

D+L pooled ES | 1.190 0.168 8.452 0.12

---------------------+---------------------------------------------------

23B Latin America

Parra (2013) | 1.190 0.168 8.452 0.12

Sub-total |

D+L pooled ES | 1.190 0.168 8.452 0.12

---------------------+---------------------------------------------------

15C Latin America

Parra (2013) | 2.381 0.595 9.520 0.15

Sub-total |

D+L pooled ES | 2.381 0.595 9.520 0.15

---------------------+---------------------------------------------------

6C Latin America

Parra (2013) | 0.001 0.000 7.0e+34 0.00

Sub-total |

D+L pooled ES | 0.001 0.000 7.0e+34 0.00

---------------------+---------------------------------------------------

pcv13 Latin America

Parra (2013) | 70.238 54.419 90.655 0.21

Sub-total |

D+L pooled ES | 70.238 54.419 90.655 0.21

---------------------+---------------------------------------------------

non-pcv Latin Americ

Parra (2013) | 29.762 20.110 44.046 0.21

Sub-total |

D+L pooled ES | 29.762 20.110 44.046 0.21

---------------------+---------------------------------------------------

24F Latin America

Parra (2013) | 1.190 0.168 8.452 0.12

Sub-total |

D+L pooled ES | 1.190 0.168 8.452 0.12

---------------------+---------------------------------------------------

8 Latin America

Parra (2013) | 2.381 0.595 9.520 0.15

Sub-total |

D+L pooled ES | 2.381 0.595 9.520 0.15

---------------------+---------------------------------------------------

pcv10-pcv7 Latin Ame

Parra (2013) | 10.714 5.575 20.592 0.20

Sub-total |

D+L pooled ES | 10.714 5.575 20.592 0.20

---------------------+---------------------------------------------------

23A Latin America

Parra (2013) | 0.001 0.000 7.0e+34 0.00

Sub-total |

D+L pooled ES | 0.001 0.000 7.0e+34 0.00

---------------------+---------------------------------------------------

9N Latin America

Parra (2013) | 0.001 0.000 7.0e+34 0.00

Sub-total |

D+L pooled ES | 0.001 0.000 7.0e+34 0.00

---------------------+---------------------------------------------------

10A Latin America

Parra (2013) | 0.001 0.000 7.0e+34 0.00

Sub-total |

D+L pooled ES | 0.001 0.000 7.0e+34 0.00

---------------------+---------------------------------------------------

11A Latin America

Parra (2013) | 2.381 0.595 9.520 0.15

Sub-total |

D+L pooled ES | 2.381 0.595 9.520 0.15

---------------------+---------------------------------------------------

38 Latin America

Parra (2013) | 0.001 0.000 7.0e+34 0.00

Sub-total |

D+L pooled ES | 0.001 0.000 7.0e+34 0.00

---------------------+---------------------------------------------------

33F Latin America

Parra (2013) | 0.001 0.000 7.0e+34 0.00

Sub-total |

D+L pooled ES | 0.001 0.000 7.0e+34 0.00

---------------------+---------------------------------------------------

19A Latin America

Parra (2013) | 5.952 2.478 14.301 0.18

Sub-total |

D+L pooled ES | 5.952 2.478 14.301 0.18

---------------------+---------------------------------------------------

15B Latin America

Parra (2013) | 3.571 1.152 11.074 0.17

Sub-total |

D+L pooled ES | 3.571 1.152 11.074 0.17

---------------------+---------------------------------------------------

3 Latin America

Parra (2013) | 14.286 8.113 25.155 0.20

Sub-total |

D+L pooled ES | 14.286 8.113 25.155 0.20

---------------------+---------------------------------------------------

15B North America

Bettinger (2010) | 2.830 1.271 6.300 0.19

De Wals (2012) | 1.770 0.443 7.077 0.15

Kellner (2009) | 0.001 0.000 8.7e+34 0.00

Black (2007) | 1.190 0.168 8.452 0.12

Bruce (2015) | 0.794 0.112 5.634 0.12

Byington (2005) | 0.000 0.000 5.6e+34 0.00

Croney (2013) | 0.000 0.000 3.7e+34 0.00

Hsu (2010) | 0.000 0.000 4.5e+34 0.00

Kaplan (2013) | 2.627 1.610 4.289 0.20

Pilishvili (2010) | 0.000 0.000 1.1e+34 0.00

Schutze (2004) | 0.001 0.000 7.8e+34 0.00

Sharma (2013)* | 0.001 0.000 1.2e+35 0.00

Weatherholtz (2010) | 0.000 0.000 5.1e+34 0.00

Sub-total |

D+L pooled ES | 2.399 1.633 3.523 0.77

---------------------+---------------------------------------------------

6C North America

Bettinger (2010) | 0.000 0.000 2.8e+34 0.00

De Wals (2012) | 1.770 0.443 7.077 0.15

Kellner (2009) | 0.001 0.000 8.7e+34 0.00

Black (2007) | 0.001 0.000 7.0e+34 0.00

Bruce (2015) | 1.587 0.397 6.347 0.15

Byington (2005) | 0.000 0.000 5.6e+34 0.00

Croney (2013) | 4.459 2.126 9.353 0.19

Hsu (2010) | 0.000 0.000 4.5e+34 0.00

Kaplan (2013) | 3.612 2.379 5.486 0.21

Pilishvili (2010) | 0.000 0.000 1.1e+34 0.00

Schutze (2004) | 0.001 0.000 7.8e+34 0.00

Sharma (2013)* | 0.001 0.000 1.2e+35 0.00

Weatherholtz (2010) | 0.000 0.000 5.1e+34 0.00

Sub-total |

D+L pooled ES | 3.437 2.444 4.835 0.70

---------------------+---------------------------------------------------

15BC North America

Bettinger (2010) | 4.245 2.209 8.159 0.20

De Wals (2012) | 4.425 1.842 10.631 0.18

Kellner (2009) | 0.001 0.000 8.7e+34 0.00

Black (2007) | 8.333 3.973 17.480 0.19

Bruce (2015) | 1.587 0.397 6.347 0.15

Byington (2005) | 0.000 0.000 5.6e+34 0.00

Croney (2013) | 0.000 0.000 3.7e+34 0.00

Hsu (2010) | 0.000 0.000 4.5e+34 0.00

Kaplan (2013) | 4.598 3.175 6.659 0.21

Pilishvili (2010) | 0.000 0.000 1.1e+34 0.00

Schutze (2004) | 0.001 0.000 7.8e+34 0.00

Sharma (2013)* | 0.001 0.000 1.2e+35 0.00

Weatherholtz (2010) | 0.000 0.000 5.1e+34 0.00

Sub-total |

D+L pooled ES | 4.697 3.570 6.180 0.93

---------------------+---------------------------------------------------

35B North America

Bettinger (2010) | 0.943 0.236 3.772 0.15

De Wals (2012) | 0.000 0.000 5.2e+34 0.00

Kellner (2009) | 0.001 0.000 8.7e+34 0.00

Black (2007) | 1.190 0.168 8.452 0.12

Bruce (2015) | 1.587 0.397 6.347 0.15

Byington (2005) | 0.000 0.000 5.6e+34 0.00

Croney (2013) | 3.185 1.326 7.651 0.18

Hsu (2010) | 0.000 0.000 4.5e+34 0.00

Kaplan (2013) | 0.000 0.000 9.6e+33 0.00

Pilishvili (2010) | 0.578 0.186 1.792 0.17

Schutze (2004) | 0.001 0.000 7.8e+34 0.00

Sharma (2013)* | 2.128 0.300 15.105 0.12

Weatherholtz (2010) | 0.000 0.000 5.1e+34 0.00

Sub-total |

D+L pooled ES | 1.519 0.900 2.565 0.88

---------------------+---------------------------------------------------

22F North America

Bettinger (2010) | 6.132 3.561 10.561 0.20

De Wals (2012) | 4.425 1.842 10.631 0.18

Kellner (2009) | 5.970 2.241 15.907 0.18

Black (2007) | 9.524 4.763 19.044 0.19

Bruce (2015) | 3.968 1.652 9.534 0.18

Byington (2005) | 0.000 0.000 5.6e+34 0.00

Croney (2013) | 0.000 0.000 3.7e+34 0.00

Hsu (2010) | 6.154 3.077 12.305 0.19

Kaplan (2013) | 3.448 2.248 5.289 0.21

Pilishvili (2010) | 4.817 3.255 7.129 0.21

Schutze (2004) | 0.001 0.000 7.8e+34 0.00

Sharma (2013)* | 4.255 1.064 17.015 0.15

Weatherholtz (2010) | 3.478 1.305 9.268 0.18

Sub-total |

D+L pooled ES | 4.890 3.999 5.979 1.87

---------------------+---------------------------------------------------

23B North America

Bettinger (2010) | 1.415 0.456 4.388 0.17

De Wals (2012) | 0.885 0.125 6.283 0.12

Kellner (2009) | 0.001 0.000 8.7e+34 0.00

Black (2007) | 1.190 0.168 8.452 0.12

Bruce (2015) | 3.968 1.652 9.534 0.18

Byington (2005) | 0.000 0.000 5.6e+34 0.00

Croney (2013) | 1.911 0.616 5.925 0.17

Hsu (2010) | 0.000 0.000 4.5e+34 0.00

Kaplan (2013) | 2.299 1.361 3.882 0.20

Pilishvili (2010) | 1.349 0.643 2.829 0.19

Schutze (2004) | 0.001 0.000 7.8e+34 0.00

Sharma (2013)* | 2.128 0.300 15.105 0.12

Weatherholtz (2010) | 1.739 0.435 6.954 0.15

Sub-total |

D+L pooled ES | 1.993 1.444 2.751 1.41

---------------------+---------------------------------------------------

9N North America

Bettinger (2010) | 0.472 0.066 3.349 0.12

De Wals (2012) | 0.000 0.000 5.2e+34 0.00

Kellner (2009) | 0.001 0.000 8.7e+34 0.00

Black (2007) | 0.001 0.000 7.0e+34 0.00

Bruce (2015) | 1.587 0.397 6.347 0.15

Byington (2005) | 0.000 0.000 5.6e+34 0.00

Croney (2013) | 0.637 0.090 4.522 0.12

Hsu (2010) | 0.000 0.000 4.5e+34 0.00

Kaplan (2013) | 0.657 0.247 1.750 0.18

Pilishvili (2010) | 0.578 0.186 1.792 0.17

Schutze (2004) | 0.001 0.000 7.8e+34 0.00

Sharma (2013)* | 0.001 0.000 1.2e+35 0.00

Weatherholtz (2010) | 0.000 0.000 5.1e+34 0.00

Sub-total |

D+L pooled ES | 0.719 0.398 1.298 0.73

---------------------+---------------------------------------------------

33F North America

Bettinger (2010) | 1.887 0.708 5.027 0.18

De Wals (2012) | 3.540 1.329 9.432 0.18

Kellner (2009) | 0.001 0.000 8.7e+34 0.00

Black (2007) | 4.762 1.787 12.688 0.18

Bruce (2015) | 3.175 1.191 8.459 0.18

Byington (2005) | 0.000 0.000 5.6e+34 0.00

Croney (2013) | 0.000 0.000 3.7e+34 0.00

Hsu (2010) | 5.385 2.567 11.295 0.19

Kaplan (2013) | 0.000 0.000 9.6e+33 0.00

Pilishvili (2010) | 4.817 3.255 7.129 0.21

Schutze (2004) | 0.001 0.000 7.8e+34 0.00

Sharma (2013)* | 12.766 5.735 28.416 0.19

Weatherholtz (2010) | 0.000 0.000 5.1e+34 0.00

Sub-total |

D+L pooled ES | 4.808 3.683 6.278 1.29

---------------------+---------------------------------------------------

3 North America

Bettinger (2010) | 8.019 4.985 12.899 0.20

De Wals (2012) | 4.425 1.842 10.631 0.18

Kellner (2009) | 8.955 4.023 19.934 0.19

Black (2007) | 2.381 0.595 9.520 0.15

Bruce (2015) | 5.556 2.648 11.654 0.19

Byington (2005) | 9.524 5.124 17.701 0.20

Croney (2013) | 3.822 1.717 8.507 0.19

Hsu (2010) | 4.615 2.073 10.273 0.19

Kaplan (2013) | 7.718 5.799 10.272 0.21

Pilishvili (2010) | 5.010 3.411 7.358 0.21

Schutze (2004) | 1.333 0.188 9.466 0.12

Sharma (2013)* | 2.128 0.300 15.105 0.12

Weatherholtz (2010) | 8.696 4.679 16.161 0.20

Sub-total |

D+L pooled ES | 6.285 5.097 7.751 2.33

---------------------+---------------------------------------------------

pcv7 North America

Bettinger (2010) | 18.396 13.441 25.179 0.21

De Wals (2012) | 4.425 1.842 10.631 0.18

Kellner (2009) | 52.239 37.507 72.757 0.21

Black (2007) | 11.905 6.405 22.126 0.20

Bruce (2015) | 2.381 0.768 7.382 0.17

Byington (2005) | 6.667 3.178 13.984 0.19

Croney (2013) | 12.102 7.719 18.973 0.20

Hsu (2010) | 11.538 6.956 19.140 0.20

Kaplan (2013) | 3.941 2.641 5.880 0.21

Pilishvili (2010) | 2.119 1.174 3.827 0.20

Schutze (2004) | 6.667 2.775 16.017 0.18

Sharma (2013)* | 0.001 0.000 1.2e+35 0.00

Weatherholtz (2010) | 15.652 9.861 24.843 0.20

Sub-total |

D+L pooled ES | 8.606 4.872 15.200 2.35

---------------------+---------------------------------------------------

pcv13 North America

Bettinger (2010) | 69.811 59.423 82.015 0.21

De Wals (2012) | 63.717 50.575 80.273 0.21

Kellner (2009) | 76.119 57.850 100.159 0.21

Black (2007) | 34.524 23.991 49.680 0.21

Bruce (2015) | 61.905 49.584 77.287 0.21

Byington (2005) | 74.286 59.501 92.744 0.21

Croney (2013) | 60.510 49.487 73.987 0.21

Hsu (2010) | 66.154 53.551 81.723 0.21

Kaplan (2013) | 67.652 61.425 74.510 0.21

Pilishvili (2010) | 67.823 61.095 75.291 0.21

Schutze (2004) | 77.333 59.786 100.032 0.21

Sharma (2013)* | 59.574 41.133 86.283 0.21

Weatherholtz (2010) | 76.522 62.093 94.303 0.21

Sub-total |

D+L pooled ES | 66.785 62.300 71.592 2.74

---------------------+---------------------------------------------------

11A North America

Bettinger (2010) | 1.415 0.456 4.388 0.17

De Wals (2012) | 0.885 0.125 6.283 0.12

Kellner (2009) | 0.001 0.000 8.7e+34 0.00

Black (2007) | 2.381 0.595 9.520 0.15

Bruce (2015) | 0.794 0.112 5.634 0.12

Byington (2005) | 0.000 0.000 5.6e+34 0.00

Croney (2013) | 0.000 0.000 3.7e+34 0.00

Hsu (2010) | 0.000 0.000 4.5e+34 0.00

Kaplan (2013) | 0.000 0.000 9.6e+33 0.00

Pilishvili (2010) | 0.771 0.289 2.054 0.18

Schutze (2004) | 0.001 0.000 7.8e+34 0.00

Sharma (2013)* | 0.001 0.000 1.2e+35 0.00

Weatherholtz (2010) | 0.000 0.000 5.1e+34 0.00

Sub-total |

D+L pooled ES | 1.131 0.626 2.041 0.73

---------------------+---------------------------------------------------

pcv10-pcv7 North Ame

Bettinger (2010) | 18.868 13.840 25.723 0.21

De Wals (2012) | 8.850 4.761 16.448 0.20

Kellner (2009) | 7.463 3.106 17.930 0.18

Black (2007) | 4.762 1.787 12.688 0.18

Bruce (2015) | 19.841 13.407 29.364 0.21

Byington (2005) | 10.476 5.802 18.917 0.20

Croney (2013) | 11.465 7.223 18.197 0.20

Hsu (2010) | 13.846 8.724 21.977 0.20

Kaplan (2013) | 15.599 12.758 19.074 0.21

Pilishvili (2010) | 9.827 7.468 12.930 0.21

Schutze (2004) | 17.333 10.065 29.852 0.20

Sharma (2013)* | 8.511 3.194 22.676 0.18

Weatherholtz (2010) | 28.696 20.400 40.364 0.21

Sub-total |

D+L pooled ES | 13.569 10.805 17.040 2.58

---------------------+---------------------------------------------------

10A North America

Bettinger (2010) | 1.887 0.708 5.027 0.18

De Wals (2012) | 2.655 0.856 8.232 0.17

Kellner (2009) | 0.001 0.000 8.7e+34 0.00

Black (2007) | 3.571 1.152 11.074 0.17

Bruce (2015) | 1.587 0.397 6.347 0.15

Byington (2005) | 0.000 0.000 5.6e+34 0.00

Croney (2013) | 0.000 0.000 3.7e+34 0.00

Hsu (2010) | 1.538 0.385 6.152 0.15

Kaplan (2013) | 0.000 0.000 9.6e+33 0.00

Pilishvili (2010) | 2.697 1.598 4.555 0.20

Schutze (2004) | 0.001 0.000 7.8e+34 0.00

Sharma (2013)* | 0.001 0.000 1.2e+35 0.00

Weatherholtz (2010) | 2.609 0.841 8.089 0.17

Sub-total |

D+L pooled ES | 2.453 1.725 3.488 1.18

---------------------+---------------------------------------------------

23A North America

Bettinger (2010) | 0.000 0.000 2.8e+34 0.00

De Wals (2012) | 1.770 0.443 7.077 0.15

Kellner (2009) | 0.001 0.000 8.7e+34 0.00

Black (2007) | 0.001 0.000 7.0e+34 0.00

Bruce (2015) | 0.000 0.000 4.6e+34 0.00

Byington (2005) | 0.000 0.000 5.6e+34 0.00

Croney (2013) | 2.548 0.956 6.788 0.18

Hsu (2010) | 0.000 0.000 4.5e+34 0.00

Kaplan (2013) | 1.642 0.883 3.052 0.20

Pilishvili (2010) | 1.734 0.902 3.333 0.20

Schutze (2004) | 0.001 0.000 7.8e+34 0.00

Sharma (2013)* | 0.001 0.000 1.2e+35 0.00

Weatherholtz (2010) | 0.000 0.000 5.1e+34 0.00

Sub-total |

D+L pooled ES | 1.805 1.219 2.671 0.72

---------------------+---------------------------------------------------

non-pcv North Americ

Bettinger (2010) | 30.189 23.629 38.570 0.21

De Wals (2012) | 36.283 26.716 49.277 0.21

Kellner (2009) | 23.881 14.630 38.981 0.20

Black (2007) | 65.476 50.270 85.283 0.21

Bruce (2015) | 38.095 28.708 50.551 0.21

Byington (2005) | 25.714 17.634 37.497 0.21

Croney (2013) | 39.490 30.788 50.652 0.21

Hsu (2010) | 33.846 25.187 45.482 0.21

Kaplan (2013) | 32.348 28.132 37.196 0.21

Pilishvili (2010) | 32.177 27.649 37.447 0.21

Schutze (2004) | 22.667 14.091 36.462 0.20

Sharma (2013)* | 40.426 25.785 63.378 0.20

Weatherholtz (2010) | 23.478 16.101 34.236 0.21

Sub-total |

D+L pooled ES | 33.696 29.355 38.679 2.71

---------------------+---------------------------------------------------

8 North America

Bettinger (2010) | 0.000 0.000 2.8e+34 0.00

De Wals (2012) | 0.000 0.000 5.2e+34 0.00

Kellner (2009) | 1.493 0.210 10.596 0.12

Black (2007) | 1.190 0.168 8.452 0.12

Bruce (2015) | 0.794 0.112 5.634 0.12

Byington (2005) | 1.905 0.476 7.616 0.15

Croney (2013) | 0.000 0.000 3.7e+34 0.00

Hsu (2010) | 0.000 0.000 4.5e+34 0.00

Kaplan (2013) | 0.493 0.159 1.527 0.17

Pilishvili (2010) | 0.000 0.000 1.1e+34 0.00

Schutze (2004) | 0.001 0.000 7.8e+34 0.00

Sharma (2013)* | 0.001 0.000 1.2e+35 0.00

Weatherholtz (2010) | 0.870 0.122 6.173 0.12

Sub-total |

D+L pooled ES | 0.929 0.484 1.786 0.78

---------------------+---------------------------------------------------

38 North America

Bettinger (2010) | 2.830 1.271 6.300 0.19

De Wals (2012) | 1.770 0.443 7.077 0.15

Kellner (2009) | 1.493 0.210 10.596 0.12

Black (2007) | 8.333 3.973 17.480 0.19

Bruce (2015) | 0.000 0.000 4.6e+34 0.00

Byington (2005) | 0.000 0.000 5.6e+34 0.00

Croney (2013) | 0.000 0.000 3.7e+34 0.00

Hsu (2010) | 0.000 0.000 4.5e+34 0.00

Kaplan (2013) | 0.985 0.443 2.193 0.19

Pilishvili (2010) | 2.697 1.598 4.555 0.20

Schutze (2004) | 0.001 0.000 7.8e+34 0.00

Sharma (2013)* | 4.255 1.064 17.015 0.15

Weatherholtz (2010) | 2.609 0.841 8.089 0.17

Sub-total |

D+L pooled ES | 2.740 1.797 4.178 1.35

---------------------+---------------------------------------------------

12F North America

Bettinger (2010) | 0.000 0.000 2.8e+34 0.00

De Wals (2012) | 0.000 0.000 5.2e+34 0.00

Kellner (2009) | 0.001 0.000 8.7e+34 0.00

Black (2007) | 1.190 0.168 8.452 0.12

Bruce (2015) | 4.762 2.139 10.600 0.19

Byington (2005) | 0.000 0.000 5.6e+34 0.00

Croney (2013) | 0.000 0.000 3.7e+34 0.00

Hsu (2010) | 1.538 0.385 6.152 0.15

Kaplan (2013) | 0.000 0.000 9.6e+33 0.00

Pilishvili (2010) | 1.541 0.771 3.082 0.19

Schutze (2004) | 0.001 0.000 7.8e+34 0.00

Sharma (2013)* | 2.128 0.300 15.105 0.12

Weatherholtz (2010) | 9.565 5.297 17.272 0.20

Sub-total |

D+L pooled ES | 3.110 1.599 6.045 0.96

---------------------+---------------------------------------------------

24F North America

Bettinger (2010) | 0.000 0.000 2.8e+34 0.00

De Wals (2012) | 0.000 0.000 5.2e+34 0.00

Kellner (2009) | 0.001 0.000 8.7e+34 0.00

Black (2007) | 0.001 0.000 7.0e+34 0.00

Bruce (2015) | 0.000 0.000 4.6e+34 0.00

Byington (2005) | 0.000 0.000 5.6e+34 0.00

Croney (2013) | 0.000 0.000 3.7e+34 0.00

Hsu (2010) | 0.000 0.000 4.5e+34 0.00

Kaplan (2013) | 0.000 0.000 9.6e+33 0.00

Pilishvili (2010) | 0.000 0.000 1.1e+34 0.00

Schutze (2004) | 0.001 0.000 7.8e+34 0.00

Sharma (2013)* | 0.001 0.000 1.2e+35 0.00

Weatherholtz (2010) | 0.000 0.000 5.1e+34 0.00

Sub-total |

D+L pooled ES | 0.000 0.000 1.3e+07 0.00

---------------------+---------------------------------------------------

15A North America

Bettinger (2010) | 0.943 0.236 3.772 0.15

De Wals (2012) | 2.655 0.856 8.232 0.17

Kellner (2009) | 0.001 0.000 8.7e+34 0.00

Black (2007) | 0.001 0.000 7.0e+34 0.00

Bruce (2015) | 2.381 0.768 7.382 0.17

Byington (2005) | 0.000 0.000 5.6e+34 0.00

Croney (2013) | 0.000 0.000 3.7e+34 0.00

Hsu (2010) | 0.000 0.000 4.5e+34 0.00

Kaplan (2013) | 0.985 0.443 2.193 0.19

Pilishvili (2010) | 1.541 0.771 3.082 0.19

Schutze (2004) | 0.001 0.000 7.8e+34 0.00

Sharma (2013)* | 0.001 0.000 1.2e+35 0.00

Weatherholtz (2010) | 0.000 0.000 5.1e+34 0.00

Sub-total |

D+L pooled ES | 1.489 0.980 2.261 0.86

---------------------+---------------------------------------------------

15C North America

Bettinger (2010) | 1.415 0.456 4.388 0.17

De Wals (2012) | 2.655 0.856 8.232 0.17

Kellner (2009) | 0.001 0.000 8.7e+34 0.00

Black (2007) | 7.143 3.209 15.899 0.19

Bruce (2015) | 0.794 0.112 5.634 0.12

Byington (2005) | 0.000 0.000 5.6e+34 0.00

Croney (2013) | 0.000 0.000 3.7e+34 0.00

Hsu (2010) | 0.000 0.000 4.5e+34 0.00

Kaplan (2013) | 1.970 1.119 3.470 0.20

Pilishvili (2010) | 0.000 0.000 1.1e+34 0.00

Schutze (2004) | 0.001 0.000 7.8e+34 0.00

Sharma (2013)* | 0.001 0.000 1.2e+35 0.00

Weatherholtz (2010) | 0.000 0.000 5.1e+34 0.00

Sub-total |

D+L pooled ES | 2.574 1.740 3.810 0.84

---------------------+---------------------------------------------------

19A North America

Bettinger (2010) | 20.283 15.043 27.349 0.21

De Wals (2012) | 43.363 32.773 57.375 0.21

Kellner (2009) | 4.478 1.444 13.883 0.17

Black (2007) | 11.905 6.405 22.126 0.20

Bruce (2015) | 31.746 23.286 43.279 0.21

Byington (2005) | 0.000 0.000 5.6e+34 0.00

Croney (2013) | 32.484 24.687 42.743 0.21

Hsu (2010) | 23.846 16.770 33.908 0.21

Kaplan (2013) | 40.066 35.341 45.422 0.21

Pilishvili (2010) | 47.206 41.650 53.503 0.21

Schutze (2004) | 0.001 0.000 7.8e+34 0.00

Sharma (2013)* | 48.936 32.519 73.641 0.21

Weatherholtz (2010) | 19.130 12.596 29.054 0.21

Sub-total |

D+L pooled ES | 28.940 23.029 36.369 2.24

---------------------+---------------------------------------------------

6A North America

Bettinger (2010) | 4.245 2.209 8.159 0.20

De Wals (2012) | 2.655 0.856 8.232 0.17

Kellner (2009) | 2.985 0.747 11.936 0.15

Black (2007) | 3.571 1.152 11.074 0.17

Bruce (2015) | 2.381 0.768 7.382 0.17

Byington (2005) | 0.000 0.000 5.6e+34 0.00

Croney (2013) | 0.637 0.090 4.522 0.12

Hsu (2010) | 12.308 7.540 20.090 0.20

Kaplan (2013) | 0.328 0.082 1.313 0.15

Pilishvili (2010) | 0.000 0.000 1.1e+34 0.00

Schutze (2004) | 0.001 0.000 7.8e+34 0.00

Sharma (2013)* | 0.001 0.000 1.2e+35 0.00

Weatherholtz (2010) | 4.348 1.810 10.446 0.18

Sub-total |

D+L pooled ES | 2.916 1.510 5.631 1.50

---------------------+---------------------------------------------------

22F Western Pacific

Williams (2011) | 4.478 2.330 8.606 0.20

Chiba (2014) | 4.636 2.746 7.827 0.20

Ishiwada (2014) | 6.061 1.516 24.234 0.15

Suga (2015) | 2.273 1.083 4.767 0.19

Sub-total |

D+L pooled ES | 3.994 2.824 5.649 0.74

---------------------+---------------------------------------------------

12F Western Pacific

Williams (2011) | 0.000 0.000 2.9e+34 0.00

Chiba (2014) | 0.000 0.000 1.9e+34 0.00

Ishiwada (2014) | 0.002 0.000 1.8e+35 0.00

Suga (2015) | 0.325 0.046 2.305 0.12

Sub-total |

D+L pooled ES | 0.321 0.045 2.279 0.12

---------------------+---------------------------------------------------

24F Western Pacific

Williams (2011) | 0.000 0.000 2.9e+34 0.00

Chiba (2014) | 0.000 0.000 1.9e+34 0.00

Ishiwada (2014) | 6.061 1.516 24.234 0.15

Suga (2015) | 4.870 2.936 8.078 0.20

Sub-total |

D+L pooled ES | 4.994 3.105 8.033 0.35

---------------------+---------------------------------------------------

pcv10-pcv7 Western P

Williams (2011) | 2.488 1.035 5.977 0.18

Chiba (2014) | 0.993 0.320 3.080 0.17

Ishiwada (2014) | 3.030 0.427 21.513 0.12

Suga (2015) | 0.649 0.162 2.596 0.15

Sub-total |

D+L pooled ES | 1.499 0.763 2.947 0.62

---------------------+---------------------------------------------------

pcv13 Western Pacifi

Williams (2011) | 67.164 56.738 79.506 0.21

Chiba (2014) | 54.967 47.210 63.998 0.21

Ishiwada (2014) | 51.515 32.025 82.868 0.20

Suga (2015) | 68.831 60.162 78.749 0.21

Sub-total |

D+L pooled ES | 62.539 54.873 71.277 0.84

---------------------+---------------------------------------------------

11A Western Pacific

Williams (2011) | 2.985 1.341 6.645 0.19

Chiba (2014) | 0.000 0.000 1.9e+34 0.00

Ishiwada (2014) | 0.002 0.000 1.8e+35 0.00

Suga (2015) | 0.649 0.162 2.596 0.15

Sub-total |

D+L pooled ES | 1.774 0.689 4.573 0.34

---------------------+---------------------------------------------------

pcv7 Western Pacific

Williams (2011) | 18.905 13.756 25.982 0.21

Chiba (2014) | 34.106 28.116 41.372 0.21

Ishiwada (2014) | 15.152 6.306 36.403 0.18

Suga (2015) | 36.364 30.216 43.762 0.21

Sub-total |

D+L pooled ES | 27.464 19.848 38.003 0.81

---------------------+---------------------------------------------------

35B Western Pacific

Williams (2011) | 0.000 0.000 2.9e+34 0.00

Chiba (2014) | 1.987 0.893 4.422 0.19

Ishiwada (2014) | 3.030 0.427 21.513 0.12

Suga (2015) | 0.649 0.162 2.596 0.15

Sub-total |

D+L pooled ES | 1.623 0.845 3.120 0.45

---------------------+---------------------------------------------------

15B Western Pacific

Williams (2011) | 0.000 0.000 2.9e+34 0.00

Chiba (2014) | 3.311 1.782 6.154 0.20

Ishiwada (2014) | 0.002 0.000 1.8e+35 0.00

Suga (2015) | 2.273 1.083 4.767 0.19

Sub-total |

D+L pooled ES | 2.834 1.762 4.560 0.39

---------------------+---------------------------------------------------

15C Western Pacific

Williams (2011) | 0.000 0.000 2.9e+34 0.00

Chiba (2014) | 4.967 2.994 8.239 0.20

Ishiwada (2014) | 3.030 0.427 21.513 0.12

Suga (2015) | 4.545 2.692 7.675 0.20

Sub-total |

D+L pooled ES | 4.687 3.277 6.703 0.52

---------------------+---------------------------------------------------

19A Western Pacific

Williams (2011) | 36.816 29.315 46.237 0.21

Chiba (2014) | 15.894 11.978 21.091 0.21

Ishiwada (2014) | 27.273 14.190 52.416 0.20

Suga (2015) | 27.922 22.603 34.493 0.21

Sub-total |

D+L pooled ES | 25.862 17.661 37.871 0.83

---------------------+---------------------------------------------------

23A Western Pacific

Williams (2011) | 0.000 0.000 2.9e+34 0.00

Chiba (2014) | 3.642 2.017 6.577 0.20

Ishiwada (2014) | 0.002 0.000 1.8e+35 0.00

Suga (2015) | 0.325 0.046 2.305 0.12

Sub-total |

D+L pooled ES | 1.449 0.231 9.100 0.31

---------------------+---------------------------------------------------

9N Western Pacific

Williams (2011) | 0.000 0.000 2.9e+34 0.00

Chiba (2014) | 0.000 0.000 1.9e+34 0.00

Ishiwada (2014) | 0.002 0.000 1.8e+35 0.00

Suga (2015) | 0.000 0.000 1.9e+34 0.00

Sub-total |

D+L pooled ES | 0.000 0.000 3.4e+15 0.00

---------------------+---------------------------------------------------

6A Western Pacific

Williams (2011) | 4.478 2.330 8.606 0.20

Chiba (2014) | 1.987 0.893 4.422 0.19

Ishiwada (2014) | 3.030 0.427 21.513 0.12

Suga (2015) | 2.597 1.299 5.194 0.19

Sub-total |

D+L pooled ES | 2.999 2.010 4.474 0.69

---------------------+---------------------------------------------------

15BC Western Pacific

Williams (2011) | 0.000 0.000 2.9e+34 0.00

Chiba (2014) | 8.278 5.594 12.251 0.21

Ishiwada (2014) | 3.030 0.427 21.513 0.12

Suga (2015) | 6.818 4.445 10.457 0.21

Sub-total |

D+L pooled ES | 7.429 5.582 9.888 0.53

---------------------+---------------------------------------------------

10A Western Pacific

Williams (2011) | 0.000 0.000 2.9e+34 0.00

Chiba (2014) | 0.993 0.320 3.080 0.17

Ishiwada (2014) | 6.061 1.516 24.234 0.15

Suga (2015) | 2.922 1.520 5.616 0.20

Sub-total |

D+L pooled ES | 2.513 1.179 5.356 0.51

---------------------+---------------------------------------------------

15A Western Pacific

Williams (2011) | 0.000 0.000 2.9e+34 0.00

Chiba (2014) | 6.623 4.273 10.265 0.20

Ishiwada (2014) | 15.152 6.306 36.403 0.18

Suga (2015) | 5.844 3.682 9.276 0.20

Sub-total |

D+L pooled ES | 7.114 4.965 10.192 0.59

---------------------+---------------------------------------------------

3 Western Pacific

Williams (2011) | 4.478 2.330 8.606 0.20

Chiba (2014) | 1.987 0.893 4.422 0.19

Ishiwada (2014) | 3.030 0.427 21.513 0.12

Suga (2015) | 1.299 0.487 3.460 0.18

Sub-total |

D+L pooled ES | 2.508 1.361 4.620 0.67

---------------------+---------------------------------------------------

non-pcv Western Paci

Williams (2011) | 32.836 25.797 41.795 0.21

Chiba (2014) | 45.033 38.066 53.275 0.21

Ishiwada (2014) | 48.485 29.703 79.143 0.20

Suga (2015) | 31.169 25.518 38.071 0.21

Sub-total |

D+L pooled ES | 37.603 30.138 46.916 0.84

---------------------+---------------------------------------------------

38 Western Pacific

Williams (2011) | 0.000 0.000 2.9e+34 0.00

Chiba (2014) | 1.987 0.893 4.422 0.19

Ishiwada (2014) | 3.030 0.427 21.513 0.12

Suga (2015) | 1.299 0.487 3.460 0.18

Sub-total |

D+L pooled ES | 1.768 0.979 3.193 0.48

---------------------+---------------------------------------------------

23B Western Pacific

Williams (2011) | 0.000 0.000 2.9e+34 0.00

Chiba (2014) | 0.000 0.000 1.9e+34 0.00

Ishiwada (2014) | 0.002 0.000 1.8e+35 0.00

Suga (2015) | 0.000 0.000 1.9e+34 0.00

Sub-total |

D+L pooled ES | 0.000 0.000 3.4e+15 0.00

---------------------+---------------------------------------------------

6C Western Pacific

Williams (2011) | 0.000 0.000 2.9e+34 0.00

Chiba (2014) | 5.629 3.499 9.055 0.20

Ishiwada (2014) | 0.002 0.000 1.8e+35 0.00

Suga (2015) | 2.597 1.299 5.194 0.19

Sub-total |

D+L pooled ES | 4.242 2.659 6.769 0.40

---------------------+---------------------------------------------------

33F Western Pacific

Williams (2011) | 1.493 0.481 4.628 0.17

Chiba (2014) | 1.987 0.893 4.422 0.19

Ishiwada (2014) | 6.061 1.516 24.234 0.15

Suga (2015) | 1.948 0.875 4.336 0.19

Sub-total |

D+L pooled ES | 2.139 1.330 3.441 0.69

---------------------+---------------------------------------------------

8 Western Pacific

Williams (2011) | 0.000 0.000 2.9e+34 0.00

Chiba (2014) | 0.000 0.000 1.9e+34 0.00

Ishiwada (2014) | 0.002 0.000 1.8e+35 0.00

Suga (2015) | 0.000 0.000 1.9e+34 0.00

Sub-total |

D+L pooled ES | 0.000 0.000 3.4e+15 0.00

---------------------+---------------------------------------------------

Settings where PCV10/13 have been introduced

Study | ES [95% Conf. Interval] % Weight

---------------------+---------------------------------------------------

non-pcv

Al-Sheikh (2014) | 14.103 7.810 25.465 0.25

Australia Surv | 56.522 46.639 68.499 0.26

Bruce (2015) | 75.000 54.797 102.651 0.26

Demczuk (2013) | 44.470 40.288 49.085 0.26

Guevara (2014) | 48.000 27.259 84.521 0.25

Kaplan (2013) | 43.463 36.422 51.865 0.26

Knol (2015) | 78.947 58.945 105.738 0.26

Moore (2014) | 62.500 43.699 89.390 0.26

Moore (2015) | 80.791 68.577 95.180 0.26

Nakano (2015) | 71.429 58.096 87.821 0.26

New Zealand Surv | 48.718 35.449 66.954 0.26

SIREVA (Brasil) | 31.250 26.314 37.111 0.26

SIREVA (Chile) | 27.381 20.509 36.556 0.26

SIREVA (Colombia) | 25.962 19.884 33.897 0.26

SIREVA (Costa Rica) | 23.684 12.323 45.520 0.24

SIREVA (Ecuador) | 19.355 10.992 34.081 0.25

SIREVA (El Salvador) | 25.806 12.906 51.603 0.24

SIREVA (Mexico) | 33.333 23.933 46.426 0.26

SIREVA (Panama) | 11.765 5.883 23.525 0.24

SIREVA (Peru) | 30.435 14.509 63.841 0.24

SIREVA (Uruguay) | 50.000 37.680 66.349 0.26

Scotland Surv | 80.097 68.762 93.300 0.26

Shibl (2012) | 5.556 2.496 12.366 0.23

Singapore Surv | 13.846 7.204 26.611 0.24

Steens (2013) | 44.681 29.132 68.529 0.26

Varon (2015) | 82.320 70.109 96.659 0.26

Waight (2015) | 85.830 75.020 98.198 0.26

van der Linden (2015 | 64.550 58.265 71.514 0.26

von Gottberg (2013) | 42.705 38.513 47.352 0.26

Sub-total |

D+L pooled ES | 42.243 36.060 49.487 7.43

I-V pooled ES | 53.423 51.436 55.487

---------------------+---------------------------------------------------

pcv7

Al-Sheikh (2014) | 73.077 56.368 94.739 0.26

Australia Surv | 3.261 1.465 7.258 0.23

Bruce (2015) | 3.846 0.962 15.379 0.19

Demczuk (2013) | 3.273 2.275 4.710 0.26

Guevara (2014) | 4.000 0.563 28.397 0.15

Kaplan (2013) | 3.180 1.655 6.112 0.24

Knol (2015) | 0.001 0.000 1.0e+35 0.00

Moore (2014) | 6.250 2.016 19.379 0.21

Moore (2015) | 3.955 1.885 8.296 0.24

Nakano (2015) | 0.794 0.112 5.634 0.15

New Zealand Surv | 7.692 3.456 17.122 0.23

SIREVA (Brasil) | 41.587 35.829 48.269 0.26

SIREVA (Chile) | 43.452 34.545 54.656 0.26

SIREVA (Colombia) | 40.865 33.039 50.546 0.26

SIREVA (Costa Rica) | 36.842 21.820 62.207 0.25

SIREVA (Ecuador) | 48.387 33.831 69.205 0.26

SIREVA (El Salvador) | 51.613 31.619 84.249 0.25

SIREVA (Mexico) | 17.143 10.801 27.209 0.25

SIREVA (Panama) | 10.294 4.907 21.593 0.24

SIREVA (Peru) | 34.783 17.394 69.553 0.24

SIREVA (Uruguay) | 13.542 7.863 23.322 0.25

Scotland Surv | 1.942 0.729 5.174 0.22

Shibl (2012) | 53.704 41.518 69.466 0.26

Singapore Surv | 26.154 16.259 42.071 0.25

Steens (2013) | 2.128 0.300 15.105 0.15

Varon (2015) | 5.525 2.973 10.268 0.25

Waight (2015) | 2.834 1.351 5.945 0.24

van der Linden (2015 | 4.938 3.410 7.152 0.26

von Gottberg (2013) | 25.504 22.313 29.152 0.26

Sub-total |

D+L pooled ES | 12.490 8.792 17.743 6.61

I-V pooled ES | 27.570 25.825 29.434

---------------------+---------------------------------------------------

pcv10-pcv7

Al-Sheikh (2014) | 2.564 0.641 10.253 0.19

Australia Surv | 10.870 7.013 16.848 0.26

Bruce (2015) | 3.846 0.962 15.379 0.19

Demczuk (2013) | 9.819 7.958 12.116 0.26

Guevara (2014) | 12.000 3.870 37.208 0.21

Kaplan (2013) | 15.194 11.269 20.488 0.26

Knol (2015) | 1.754 0.247 12.455 0.15

Moore (2014) | 10.417 4.336 25.027 0.23

Moore (2015) | 2.260 0.848 6.021 0.22

Nakano (2015) | 2.381 0.768 7.382 0.21

New Zealand Surv | 2.564 0.641 10.253 0.19

SIREVA (Brasil) | 2.644 1.464 4.775 0.25

SIREVA (Chile) | 8.929 5.383 14.810 0.25

SIREVA (Colombia) | 12.981 8.902 18.929 0.26

SIREVA (Costa Rica) | 15.789 7.093 35.146 0.23

SIREVA (Ecuador) | 6.452 2.421 17.190 0.22

SIREVA (El Salvador) | 6.452 1.613 25.797 0.19

SIREVA (Mexico) | 4.762 1.982 11.441 0.23

SIREVA (Panama) | 60.294 44.395 81.887 0.26

SIREVA (Peru) | 4.348 0.612 30.867 0.15

SIREVA (Uruguay) | 20.833 13.441 32.292 0.26

Scotland Surv | 8.252 5.130 13.275 0.25

Shibl (2012) | 25.000 17.144 36.455 0.26

Singapore Surv | 0.001 0.000 9.0e+34 0.00

Steens (2013) | 25.532 14.500 44.958 0.25

Varon (2015) | 0.000 0.000 3.2e+34 0.00

Waight (2015) | 5.263 3.056 9.064 0.25

van der Linden (2015 | 12.346 9.767 15.605 0.26

von Gottberg (2013) | 9.846 7.940 12.209 0.26

Sub-total |

D+L pooled ES | 9.165 6.890 12.190 6.21

I-V pooled ES | 12.371 11.358 13.475

---------------------+---------------------------------------------------

pcv13

Al-Sheikh (2014) | 85.897 67.606 109.137 0.26

Australia Surv | 43.478 34.922 54.130 0.26

Bruce (2015) | 25.000 14.516 43.055 0.25

Demczuk (2013) | 55.530 50.834 60.661 0.26

Guevara (2014) | 52.000 30.194 89.555 0.25

Kaplan (2013) | 56.537 48.422 66.013 0.26

Knol (2015) | 21.053 11.956 37.071 0.25

Moore (2014) | 37.500 23.626 59.520 0.25

Moore (2015) | 19.209 13.725 26.884 0.26

Nakano (2015) | 28.571 20.609 39.610 0.26

New Zealand Surv | 51.282 37.616 69.912 0.26

SIREVA (Brasil) | 68.750 61.226 77.198 0.26

SIREVA (Chile) | 72.619 60.811 86.719 0.26

SIREVA (Colombia) | 74.038 63.221 86.706 0.26

SIREVA (Costa Rica) | 76.316 53.033 109.820 0.26

SIREVA (Ecuador) | 80.645 61.122 106.404 0.26

SIREVA (El Salvador) | 74.194 49.303 111.650 0.26

SIREVA (Mexico) | 66.667 52.743 84.265 0.26

SIREVA (Panama) | 88.235 68.509 113.641 0.26

SIREVA (Peru) | 69.565 42.617 113.552 0.25

SIREVA (Uruguay) | 50.000 37.680 66.349 0.26

Scotland Surv | 19.903 14.655 27.031 0.26

Shibl (2012) | 94.444 77.785 114.673 0.26

Singapore Surv | 86.154 66.302 111.950 0.26

Steens (2013) | 55.319 37.665 81.248 0.26

Varon (2015) | 17.680 12.502 25.000 0.26

Waight (2015) | 14.170 10.174 19.736 0.26

van der Linden (2015 | 35.450 30.873 40.706 0.26

von Gottberg (2013) | 57.295 52.407 62.640 0.26

Sub-total |

D+L pooled ES | 49.077 42.308 56.930 7.53

I-V pooled ES | 55.695 53.669 57.797

---------------------+---------------------------------------------------

19A

Al-Sheikh (2014) | 3.846 1.240 11.926 0.21

Australia Surv | 22.826 16.869 30.887 0.26

Bruce (2015) | 15.385 7.694 30.764 0.24

Demczuk (2013) | 32.844 29.279 36.843 0.26

Guevara (2014) | 24.000 10.782 53.422 0.23

Kaplan (2013) | 33.216 27.136 40.657 0.26

Knol (2015) | 19.298 10.687 34.847 0.25

Moore (2014) | 16.667 8.335 33.327 0.24

Moore (2015) | 7.345 4.265 12.649 0.25

Nakano (2015) | 24.603 17.302 34.984 0.26

New Zealand Surv | 26.923 17.554 41.293 0.26

SIREVA (Brasil) | 8.413 6.041 11.718 0.26

SIREVA (Chile) | 8.333 4.935 14.071 0.25

SIREVA (Colombia) | 10.096 6.583 15.485 0.26

SIREVA (Costa Rica) | 18.421 8.782 38.641 0.24

SIREVA (Ecuador) | 16.129 8.678 29.977 0.25

SIREVA (El Salvador) | 16.129 6.713 38.751 0.23

SIREVA (Mexico) | 39.048 28.751 53.031 0.26

SIREVA (Panama) | 8.824 3.964 19.640 0.23

SIREVA (Peru) | 17.391 6.527 46.338 0.22

SIREVA (Uruguay) | 3.125 1.008 9.689 0.21

Scotland Surv | 5.825 3.308 10.257 0.25

Shibl (2012) | 7.407 3.704 14.812 0.24

Singapore Surv | 50.769 36.093 71.413 0.26

Steens (2013) | 21.277 11.448 39.544 0.25

Varon (2015) | 8.840 5.415 14.429 0.25

Waight (2015) | 2.834 1.351 5.945 0.24

van der Linden (2015 | 11.817 9.300 15.014 0.26

von Gottberg (2013) | 11.388 9.323 13.910 0.26

Sub-total |

D+L pooled ES | 14.245 11.061 18.345 7.15

I-V pooled ES | 20.833 19.531 22.221

---------------------+---------------------------------------------------

6A

Al-Sheikh (2014) | 2.564 0.641 10.253 0.19

Australia Surv | 0.543 0.077 3.858 0.15

Bruce (2015) | 0.001 0.000 1.1e+35 0.00

Demczuk (2013) | 1.129 0.607 2.098 0.25

Guevara (2014) | 0.002 0.000 2.3e+35 0.00

Kaplan (2013) | 0.353 0.050 2.509 0.15

Knol (2015) | 0.001 0.000 1.0e+35 0.00

Moore (2014) | 0.001 0.000 1.2e+35 0.00

Moore (2015) | 0.000 0.000 3.3e+34 0.00

Nakano (2015) | 0.000 0.000 4.6e+34 0.00

New Zealand Surv | 0.001 0.000 7.5e+34 0.00

SIREVA (Brasil) | 5.529 3.674 8.320 0.26

SIREVA (Chile) | 7.143 4.056 12.578 0.25

SIREVA (Colombia) | 4.327 2.251 8.316 0.24

SIREVA (Costa Rica) | 0.001 0.000 1.5e+35 0.00

SIREVA (Ecuador) | 6.452 2.421 17.190 0.22

SIREVA (El Salvador) | 0.002 0.000 1.9e+35 0.00

SIREVA (Mexico) | 1.905 0.476 7.616 0.19

SIREVA (Panama) | 5.882 2.208 15.673 0.22

SIREVA (Peru) | 4.348 0.612 30.867 0.15

SIREVA (Uruguay) | 0.001 0.000 6.1e+34 0.00

Scotland Surv | 0.000 0.000 2.8e+34 0.00

Shibl (2012) | 6.481 3.090 13.596 0.24

Singapore Surv | 0.001 0.000 9.0e+34 0.00

Steens (2013) | 0.001 0.000 1.2e+35 0.00

Varon (2015) | 0.552 0.078 3.922 0.15

Waight (2015) | 0.000 0.000 2.4e+34 0.00

van der Linden (2015 | 1.058 0.475 2.355 0.23

von Gottberg (2013) | 8.541 6.779 10.760 0.26

Sub-total |

D+L pooled ES | 3.214 2.093 4.935 3.16

I-V pooled ES | 5.423 4.633 6.348

---------------------+---------------------------------------------------

3

Al-Sheikh (2014) | 2.564 0.641 10.253 0.19

Australia Surv | 5.978 3.311 10.795 0.25

Bruce (2015) | 1.923 0.271 13.653 0.15

Demczuk (2013) | 8.465 6.751 10.615 0.26

Guevara (2014) | 12.000 3.870 37.208 0.21

Kaplan (2013) | 4.594 2.667 7.911 0.25

Knol (2015) | 0.001 0.000 1.0e+35 0.00

Moore (2014) | 4.167 1.042 16.661 0.19

Moore (2015) | 5.650 3.040 10.500 0.25

Nakano (2015) | 0.794 0.112 5.634 0.15

New Zealand Surv | 14.103 7.810 25.465 0.25

SIREVA (Brasil) | 10.577 7.871 14.213 0.26

SIREVA (Chile) | 4.762 2.381 9.522 0.24

SIREVA (Colombia) | 5.769 3.276 10.159 0.25

SIREVA (Costa Rica) | 5.263 1.316 21.045 0.19

SIREVA (Ecuador) | 3.226 0.807 12.899 0.19

SIREVA (El Salvador) | 0.002 0.000 1.9e+35 0.00

SIREVA (Mexico) | 3.810 1.430 10.150 0.22

SIREVA (Panama) | 1.471 0.207 10.440 0.15

SIREVA (Peru) | 8.696 2.175 34.770 0.19

SIREVA (Uruguay) | 12.500 7.099 22.011 0.25

Scotland Surv | 3.883 1.942 7.766 0.24

Shibl (2012) | 1.852 0.463 7.405 0.19

Singapore Surv | 9.231 4.147 20.547 0.23

Steens (2013) | 6.383 2.059 19.791 0.21

Varon (2015) | 2.762 1.150 6.637 0.23

Waight (2015) | 3.239 1.620 6.477 0.24

van der Linden (2015 | 5.291 3.699 7.567 0.26

von Gottberg (2013) | 2.017 1.254 3.244 0.25

Sub-total |

D+L pooled ES | 5.305 4.185 6.725 5.96

I-V pooled ES | 6.439 5.745 7.218

---------------------+---------------------------------------------------

12F

Al-Sheikh (2014) | 1.282 0.181 9.102 0.15

Australia Surv | 0.000 0.000 3.2e+34 0.00

Bruce (2015) | 7.692 2.887 20.496 0.22

Demczuk (2013) | 1.467 0.852 2.527 0.25

Guevara (2014) | 8.000 2.001 31.988 0.19

Kaplan (2013) | 0.000 0.000 2.1e+34 0.00

Knol (2015) | 3.509 0.878 14.030 0.19

Moore (2014) | 6.250 2.016 19.379 0.21

Moore (2015) | 3.955 1.885 8.296 0.24

Nakano (2015) | 0.000 0.000 4.6e+34 0.00

New Zealand Surv | 0.001 0.000 7.5e+34 0.00

SIREVA (Brasil) | 3.365 1.993 5.682 0.25

SIREVA (Chile) | 3.571 1.604 7.950 0.23

SIREVA (Colombia) | 0.000 0.000 2.8e+34 0.00

SIREVA (Costa Rica) | 0.001 0.000 1.5e+35 0.00

SIREVA (Ecuador) | 0.001 0.000 9.4e+34 0.00

SIREVA (El Salvador) | 0.002 0.000 1.9e+35 0.00

SIREVA (Mexico) | 0.952 0.134 6.761 0.15

SIREVA (Panama) | 0.001 0.000 8.6e+34 0.00

SIREVA (Peru) | 4.348 0.612 30.867 0.15

SIREVA (Uruguay) | 7.292 3.476 15.295 0.24

Scotland Surv | 4.854 2.612 9.022 0.25

Shibl (2012) | 0.000 0.000 5.4e+34 0.00

Singapore Surv | 0.001 0.000 9.0e+34 0.00

Steens (2013) | 0.001 0.000 1.2e+35 0.00

Varon (2015) | 8.840 5.415 14.429 0.25

Waight (2015) | 6.073 3.661 10.073 0.25

van der Linden (2015 | 4.056 2.696 6.104 0.26

von Gottberg (2013) | 4.033 2.882 5.645 0.26

Sub-total |

D+L pooled ES | 4.335 3.524 5.334 3.75

I-V pooled ES | 4.314 3.693 5.039

---------------------+---------------------------------------------------

10A

Al-Sheikh (2014) | 0.001 0.000 7.5e+34 0.00

Australia Surv | 2.174 0.816 5.792 0.22

Bruce (2015) | 3.846 0.962 15.379 0.19

Demczuk (2013) | 2.483 1.635 3.771 0.26

Guevara (2014) | 0.002 0.000 2.3e+35 0.00

Kaplan (2013) | 0.000 0.000 2.1e+34 0.00

Knol (2015) | 28.070 17.197 45.819 0.25

Moore (2014) | 8.333 3.128 22.204 0.22

Moore (2015) | 0.000 0.000 3.3e+34 0.00

Nakano (2015) | 3.968 1.652 9.534 0.23

New Zealand Surv | 0.001 0.000 7.5e+34 0.00

SIREVA (Brasil) | 0.962 0.361 2.562 0.22

SIREVA (Chile) | 0.595 0.084 4.226 0.15

SIREVA (Colombia) | 0.962 0.240 3.845 0.19

SIREVA (Costa Rica) | 2.632 0.371 18.682 0.15

SIREVA (Ecuador) | 1.613 0.227 11.451 0.15

SIREVA (El Salvador) | 0.002 0.000 1.9e+35 0.00

SIREVA (Mexico) | 2.857 0.921 8.859 0.21

SIREVA (Panama) | 0.001 0.000 8.6e+34 0.00

SIREVA (Peru) | 0.002 0.000 2.5e+35 0.00

SIREVA (Uruguay) | 1.042 0.147 7.395 0.15

Scotland Surv | 2.913 1.309 6.483 0.23

Shibl (2012) | 0.000 0.000 5.4e+34 0.00

Singapore Surv | 0.001 0.000 9.0e+34 0.00

Steens (2013) | 4.255 1.064 17.015 0.19

Varon (2015) | 7.735 4.581 13.060 0.25

Waight (2015) | 2.834 1.351 5.945 0.24

van der Linden (2015 | 6.878 5.025 9.414 0.26

von Gottberg (2013) | 0.000 0.000 6.9e+33 0.00

Sub-total |

D+L pooled ES | 3.440 2.201 5.376 3.78

I-V pooled ES | 5.119 4.322 6.063

---------------------+---------------------------------------------------

22F

Al-Sheikh (2014) | 0.001 0.000 7.5e+34 0.00

Australia Surv | 5.978 3.311 10.795 0.25

Bruce (2015) | 7.692 2.887 20.496 0.22

Demczuk (2013) | 6.095 4.668 7.958 0.26

Guevara (2014) | 4.000 0.563 28.397 0.15

Kaplan (2013) | 4.240 2.408 7.467 0.25

Knol (2015) | 5.263 1.697 16.319 0.21

Moore (2015) | 11.299 7.290 17.514 0.26

Nakano (2015) | 11.111 6.581 18.761 0.25

New Zealand Surv | 3.846 1.240 11.926 0.21

SIREVA (Brasil) | 1.923 0.962 3.845 0.24

SIREVA (Chile) | 2.976 1.239 7.151 0.23

SIREVA (Colombia) | 0.000 0.000 2.8e+34 0.00

SIREVA (Costa Rica) | 0.001 0.000 1.5e+35 0.00

SIREVA (Ecuador) | 0.001 0.000 9.4e+34 0.00

SIREVA (El Salvador) | 0.002 0.000 1.9e+35 0.00

SIREVA (Mexico) | 0.952 0.134 6.761 0.15

SIREVA (Panama) | 0.001 0.000 8.6e+34 0.00

SIREVA (Peru) | 0.002 0.000 2.5e+35 0.00

SIREVA (Uruguay) | 3.125 1.008 9.689 0.21

Scotland Surv | 4.369 2.273 8.397 0.24

Shibl (2012) | 0.000 0.000 5.4e+34 0.00

Singapore Surv | 0.001 0.000 9.0e+34 0.00

Steens (2013) | 4.255 1.064 17.015 0.19

Varon (2015) | 4.972 2.587 9.557 0.24

Waight (2015) | 9.312 6.188 14.013 0.26

van der Linden (2015 | 3.527 2.276 5.467 0.26

von Gottberg (2013) | 0.000 0.000 6.9e+33 0.00

Sub-total |

D+L pooled ES | 5.321 4.242 6.674 4.08

I-V pooled ES | 5.841 5.089 6.705

---------------------+---------------------------------------------------

15B

Al-Sheikh (2014) | 0.001 0.000 7.5e+34 0.00

Australia Surv | 2.174 0.816 5.792 0.22

Bruce (2015) | 7.692 2.887 20.496 0.22

Demczuk (2013) | 2.709 1.816 4.041 0.26

Guevara (2014) | 0.002 0.000 2.3e+35 0.00

Kaplan (2013) | 2.473 1.179 5.188 0.24

Knol (2015) | 1.754 0.247 12.455 0.15

Moore (2014) | 4.167 1.042 16.661 0.19

Moore (2015) | 7.345 4.265 12.649 0.25

Nakano (2015) | 0.000 0.000 4.6e+34 0.00

New Zealand Surv | 5.128 1.925 13.664 0.22

SIREVA (Brasil) | 0.240 0.034 1.707 0.15

SIREVA (Chile) | 1.190 0.298 4.760 0.19

SIREVA (Colombia) | 2.885 1.296 6.421 0.23

SIREVA (Costa Rica) | 0.001 0.000 1.5e+35 0.00

SIREVA (Ecuador) | 0.001 0.000 9.4e+34 0.00

SIREVA (El Salvador) | 0.002 0.000 1.9e+35 0.00

SIREVA (Mexico) | 4.762 1.982 11.441 0.23

SIREVA (Panama) | 0.001 0.000 8.6e+34 0.00

SIREVA (Peru) | 0.002 0.000 2.5e+35 0.00

SIREVA (Uruguay) | 0.001 0.000 6.1e+34 0.00

Scotland Surv | 3.398 1.620 7.128 0.24

Shibl (2012) | 0.000 0.000 5.4e+34 0.00

Singapore Surv | 0.001 0.000 9.0e+34 0.00

Steens (2013) | 0.001 0.000 1.2e+35 0.00

Varon (2015) | 0.000 0.000 3.2e+34 0.00

Waight (2015) | 0.000 0.000 2.4e+34 0.00

van der Linden (2015 | 3.880 2.555 5.893 0.26

von Gottberg (2013) | 4.270 3.080 5.920 0.26

Sub-total |

D+L pooled ES | 3.714 3.143 4.388 3.32

I-V pooled ES | 3.714 3.143 4.388

---------------------+---------------------------------------------------

15C

Al-Sheikh (2014) | 0.001 0.000 7.5e+34 0.00

Australia Surv | 4.348 2.174 8.694 0.24

Bruce (2015) | 5.769 1.861 17.888 0.21

Demczuk (2013) | 2.935 1.998 4.310 0.26

Guevara (2014) | 0.002 0.000 2.3e+35 0.00

Kaplan (2013) | 2.473 1.179 5.188 0.24

Knol (2015) | 7.018 2.634 18.698 0.22

Moore (2014) | 10.417 4.336 25.027 0.23

Moore (2015) | 6.780 3.850 11.938 0.25

Nakano (2015) | 0.000 0.000 4.6e+34 0.00

New Zealand Surv | 0.001 0.000 7.5e+34 0.00

SIREVA (Brasil) | 1.683 0.802 3.530 0.24

SIREVA (Chile) | 1.190 0.298 4.760 0.19

SIREVA (Colombia) | 0.000 0.000 2.8e+34 0.00

SIREVA (Costa Rica) | 0.001 0.000 1.5e+35 0.00

SIREVA (Ecuador) | 0.001 0.000 9.4e+34 0.00

SIREVA (El Salvador) | 0.002 0.000 1.9e+35 0.00

SIREVA (Mexico) | 0.952 0.134 6.761 0.15

SIREVA (Panama) | 1.471 0.207 10.440 0.15

SIREVA (Peru) | 0.002 0.000 2.5e+35 0.00

SIREVA (Uruguay) | 0.001 0.000 6.1e+34 0.00

Scotland Surv | 2.427 1.010 5.831 0.23

Shibl (2012) | 0.000 0.000 5.4e+34 0.00

Singapore Surv | 0.001 0.000 9.0e+34 0.00

Steens (2013) | 0.001 0.000 1.2e+35 0.00

Varon (2015) | 0.000 0.000 3.2e+34 0.00

Waight (2015) | 0.000 0.000 2.4e+34 0.00

van der Linden (2015 | 5.996 4.285 8.392 0.26

von Gottberg (2013) | 0.000 0.000 6.9e+33 0.00

Sub-total |

D+L pooled ES | 3.957 3.118 5.022 2.87

I-V pooled ES | 4.141 3.449 4.972

---------------------+---------------------------------------------------

24F

Al-Sheikh (2014) | 0.001 0.000 7.5e+34 0.00

Australia Surv | 0.543 0.077 3.858 0.15

Bruce (2015) | 0.001 0.000 1.1e+35 0.00

Demczuk (2013) | 0.000 0.000 6.6e+33 0.00

Guevara (2014) | 4.000 0.563 28.397 0.15

Kaplan (2013) | 0.000 0.000 2.1e+34 0.00

Knol (2015) | 1.754 0.247 12.455 0.15

Moore (2014) | 2.083 0.293 14.790 0.15

Moore (2015) | 0.000 0.000 3.3e+34 0.00

Nakano (2015) | 22.222 15.343 32.185 0.26

New Zealand Surv | 0.001 0.000 7.5e+34 0.00

SIREVA (Brasil) | 0.962 0.361 2.562 0.22

SIREVA (Chile) | 3.571 1.604 7.950 0.23

SIREVA (Colombia) | 0.962 0.240 3.845 0.19

SIREVA (Costa Rica) | 0.001 0.000 1.5e+35 0.00

SIREVA (Ecuador) | 4.839 1.561 15.003 0.21

SIREVA (El Salvador) | 0.002 0.000 1.9e+35 0.00

SIREVA (Mexico) | 0.000 0.000 5.6e+34 0.00

SIREVA (Panama) | 2.941 0.736 11.760 0.19

SIREVA (Peru) | 0.002 0.000 2.5e+35 0.00

SIREVA (Uruguay) | 3.125 1.008 9.689 0.21

Scotland Surv | 0.485 0.068 3.446 0.15

Shibl (2012) | 0.000 0.000 5.4e+34 0.00

Singapore Surv | 0.001 0.000 9.0e+34 0.00

Steens (2013) | 2.128 0.300 15.105 0.15

Varon (2015) | 19.890 14.347 27.574 0.26

Waight (2015) | 11.336 7.827 16.418 0.26

van der Linden (2015 | 8.818 6.684 11.635 0.26

von Gottberg (2013) | 0.000 0.000 6.9e+33 0.00

Sub-total |

D+L pooled ES | 4.172 2.568 6.776 3.20

I-V pooled ES | 10.254 8.815 11.928

---------------------+---------------------------------------------------

15A

Al-Sheikh (2014) | 0.001 0.000 7.5e+34 0.00

Australia Surv | 0.543 0.077 3.858 0.15

Bruce (2015) | 7.692 2.887 20.496 0.22

Demczuk (2013) | 3.725 2.648 5.239 0.26

Guevara (2014) | 0.002 0.000 2.3e+35 0.00

Kaplan (2013) | 1.060 0.342 3.287 0.21

Knol (2015) | 0.001 0.000 1.0e+35 0.00

Moore (2014) | 0.001 0.000 1.2e+35 0.00

Moore (2015) | 0.000 0.000 3.3e+34 0.00

Nakano (2015) | 11.111 6.581 18.761 0.25

New Zealand Surv | 0.001 0.000 7.5e+34 0.00

SIREVA (Brasil) | 0.721 0.233 2.236 0.21

SIREVA (Chile) | 0.595 0.084 4.226 0.15

SIREVA (Colombia) | 0.962 0.240 3.845 0.19

SIREVA (Costa Rica) | 5.263 1.316 21.045 0.19

SIREVA (Ecuador) | 1.613 0.227 11.451 0.15

SIREVA (El Salvador) | 0.002 0.000 1.9e+35 0.00

SIREVA (Mexico) | 0.000 0.000 5.6e+34 0.00

SIREVA (Panama) | 2.941 0.736 11.760 0.19

SIREVA (Peru) | 0.002 0.000 2.5e+35 0.00

SIREVA (Uruguay) | 1.042 0.147 7.395 0.15

Scotland Surv | 2.913 1.309 6.483 0.23

Shibl (2012) | 0.000 0.000 5.4e+34 0.00

Singapore Surv | 0.001 0.000 9.0e+34 0.00

Steens (2013) | 0.001 0.000 1.2e+35 0.00

Varon (2015) | 7.182 4.170 12.369 0.25

Waight (2015) | 7.287 4.591 11.567 0.25

van der Linden (2015 | 1.235 0.589 2.590 0.24

von Gottberg (2013) | 0.000 0.000 6.9e+33 0.00

Sub-total |

D+L pooled ES | 2.880 1.902 4.360 3.31

I-V pooled ES | 4.169 3.461 5.021

---------------------+---------------------------------------------------

33F

Al-Sheikh (2014) | 0.001 0.000 7.5e+34 0.00

Australia Surv | 4.891 2.545 9.401 0.24

Bruce (2015) | 5.769 1.861 17.888 0.21

Demczuk (2013) | 1.919 1.193 3.086 0.25

Guevara (2014) | 4.000 0.563 28.397 0.15

Kaplan (2013) | 5.654 3.464 9.229 0.25

Knol (2015) | 10.526 4.729 23.431 0.23

Moore (2014) | 6.250 2.016 19.379 0.21

Moore (2015) | 9.605 5.971 15.450 0.25

Nakano (2015) | 0.794 0.112 5.634 0.15

New Zealand Surv | 5.128 1.925 13.664 0.22

SIREVA (Brasil) | 0.000 0.000 1.4e+34 0.00

SIREVA (Chile) | 2.381 0.894 6.344 0.22

SIREVA (Colombia) | 0.000 0.000 2.8e+34 0.00

SIREVA (Costa Rica) | 0.001 0.000 1.5e+35 0.00

SIREVA (Ecuador) | 0.001 0.000 9.4e+34 0.00

SIREVA (El Salvador) | 0.002 0.000 1.9e+35 0.00

SIREVA (Mexico) | 0.000 0.000 5.6e+34 0.00

SIREVA (Panama) | 0.001 0.000 8.6e+34 0.00

SIREVA (Peru) | 0.002 0.000 2.5e+35 0.00

SIREVA (Uruguay) | 1.042 0.147 7.395 0.15

Scotland Surv | 2.913 1.309 6.483 0.23

Shibl (2012) | 0.000 0.000 5.4e+34 0.00

Singapore Surv | 0.001 0.000 9.0e+34 0.00

Steens (2013) | 4.255 1.064 17.015 0.19

Varon (2015) | 3.315 1.489 7.379 0.23

Waight (2015) | 8.097 5.224 12.551 0.26

van der Linden (2015 | 3.175 2.000 5.039 0.25

von Gottberg (2013) | 0.000 0.000 6.9e+33 0.00

Sub-total |

D+L pooled ES | 4.452 3.385 5.855 3.73

I-V pooled ES | 4.676 3.948 5.539

---------------------+---------------------------------------------------

6C

Al-Sheikh (2014) | 0.001 0.000 7.5e+34 0.00

Australia Surv | 2.174 0.816 5.792 0.22

Bruce (2015) | 0.001 0.000 1.1e+35 0.00

Demczuk (2013) | 1.919 1.193 3.086 0.25

Guevara (2014) | 4.000 0.563 28.397 0.15

Kaplan (2013) | 3.534 1.901 6.567 0.25

Knol (2015) | 1.754 0.247 12.455 0.15

Moore (2014) | 2.083 0.293 14.790 0.15

Moore (2015) | 1.695 0.547 5.255 0.21

Nakano (2015) | 1.587 0.397 6.347 0.19

New Zealand Surv | 7.692 3.456 17.122 0.23

SIREVA (Brasil) | 2.885 1.638 5.079 0.25

SIREVA (Chile) | 2.381 0.894 6.344 0.22

SIREVA (Colombia) | 1.442 0.465 4.472 0.21

SIREVA (Costa Rica) | 0.001 0.000 1.5e+35 0.00

SIREVA (Ecuador) | 1.613 0.227 11.451 0.15

SIREVA (El Salvador) | 0.002 0.000 1.9e+35 0.00

SIREVA (Mexico) | 1.905 0.476 7.616 0.19

SIREVA (Panama) | 0.001 0.000 8.6e+34 0.00

SIREVA (Peru) | 4.348 0.612 30.867 0.15

SIREVA (Uruguay) | 0.001 0.000 6.1e+34 0.00

Scotland Surv | 0.485 0.068 3.446 0.15

Shibl (2012) | 0.000 0.000 5.4e+34 0.00

Singapore Surv | 1.538 0.217 10.922 0.15

Steens (2013) | 8.511 3.194 22.676 0.22

Varon (2015) | 0.552 0.078 3.922 0.15

Waight (2015) | 0.810 0.203 3.238 0.19

van der Linden (2015 | 1.058 0.475 2.355 0.23

von Gottberg (2013) | 0.000 0.000 6.9e+33 0.00

Sub-total |

D+L pooled ES | 2.358 1.840 3.023 4.08

I-V pooled ES | 2.390 1.927 2.964

---------------------+---------------------------------------------------

23B

Al-Sheikh (2014) | 1.282 0.181 9.102 0.15

Australia Surv | 4.348 2.174 8.694 0.24

Bruce (2015) | 1.923 0.271 13.653 0.15

Demczuk (2013) | 2.596 1.725 3.906 0.26

Guevara (2014) | 0.002 0.000 2.3e+35 0.00

Kaplan (2013) | 2.120 0.952 4.719 0.23

Knol (2015) | 0.001 0.000 1.0e+35 0.00

Moore (2014) | 2.083 0.293 14.790 0.15

Moore (2015) | 5.085 2.646 9.773 0.24

Nakano (2015) | 0.000 0.000 4.6e+34 0.00

New Zealand Surv | 2.564 0.641 10.253 0.19

SIREVA (Brasil) | 0.481 0.120 1.922 0.19

SIREVA (Chile) | 0.000 0.000 3.5e+34 0.00

SIREVA (Colombia) | 1.442 0.465 4.472 0.21

SIREVA (Costa Rica) | 0.001 0.000 1.5e+35 0.00

SIREVA (Ecuador) | 3.226 0.807 12.899 0.19

SIREVA (El Salvador) | 0.002 0.000 1.9e+35 0.00

SIREVA (Mexico) | 4.762 1.982 11.441 0.23

SIREVA (Panama) | 0.001 0.000 8.6e+34 0.00

SIREVA (Peru) | 4.348 0.612 30.867 0.15

SIREVA (Uruguay) | 0.001 0.000 6.1e+34 0.00

Scotland Surv | 2.427 1.010 5.831 0.23

Shibl (2012) | 0.000 0.000 5.4e+34 0.00

Singapore Surv | 0.001 0.000 9.0e+34 0.00

Steens (2013) | 0.001 0.000 1.2e+35 0.00

Varon (2015) | 1.105 0.276 4.418 0.19

Waight (2015) | 7.287 4.591 11.567 0.25

van der Linden (2015 | 4.762 3.266 6.944 0.26

von Gottberg (2013) | 0.000 0.000 6.9e+33 0.00

Sub-total |

D+L pooled ES | 3.364 2.648 4.274 3.53

I-V pooled ES | 3.647 3.040 4.374

---------------------+---------------------------------------------------

11A

Al-Sheikh (2014) | 0.001 0.000 7.5e+34 0.00

Australia Surv | 0.543 0.077 3.858 0.15

Bruce (2015) | 1.923 0.271 13.653 0.15

Demczuk (2013) | 2.483 1.635 3.771 0.26

Guevara (2014) | 0.002 0.000 2.3e+35 0.00

Kaplan (2013) | 0.000 0.000 2.1e+34 0.00

Knol (2015) | 0.001 0.000 1.0e+35 0.00

Moore (2014) | 2.083 0.293 14.790 0.15

Moore (2015) | 0.000 0.000 3.3e+34 0.00

Nakano (2015) | 0.794 0.112 5.634 0.15

New Zealand Surv | 0.001 0.000 7.5e+34 0.00

SIREVA (Brasil) | 1.923 0.962 3.845 0.24

SIREVA (Chile) | 1.190 0.298 4.760 0.19

SIREVA (Colombia) | 2.885 1.296 6.421 0.23

SIREVA (Costa Rica) | 5.263 1.316 21.045 0.19

SIREVA (Ecuador) | 1.613 0.227 11.451 0.15

SIREVA (El Salvador) | 0.002 0.000 1.9e+35 0.00

SIREVA (Mexico) | 1.905 0.476 7.616 0.19

SIREVA (Panama) | 1.471 0.207 10.440 0.15

SIREVA (Peru) | 0.002 0.000 2.5e+35 0.00

SIREVA (Uruguay) | 2.083 0.521 8.330 0.19

Scotland Surv | 0.971 0.243 3.882 0.19

Shibl (2012) | 0.000 0.000 5.4e+34 0.00

Singapore Surv | 0.001 0.000 9.0e+34 0.00

Steens (2013) | 2.128 0.300 15.105 0.15

Varon (2015) | 1.105 0.276 4.418 0.19

Waight (2015) | 1.619 0.608 4.315 0.22

van der Linden (2015 | 1.940 1.074 3.503 0.25

von Gottberg (2013) | 0.000 0.000 6.9e+33 0.00

Sub-total |

D+L pooled ES | 2.028 1.605 2.564 3.40

I-V pooled ES | 2.028 1.605 2.564

---------------------+---------------------------------------------------

35B

Al-Sheikh (2014) | 0.001 0.000 7.5e+34 0.00

Australia Surv | 4.348 2.174 8.694 0.24

Bruce (2015) | 5.769 1.861 17.888 0.21

Demczuk (2013) | 2.144 1.368 3.362 0.25

Guevara (2014) | 0.002 0.000 2.3e+35 0.00

Kaplan (2013) | 0.000 0.000 2.1e+34 0.00

Knol (2015) | 1.754 0.247 12.455 0.15

Moore (2014) | 0.001 0.000 1.2e+35 0.00

Moore (2015) | 7.910 4.684 13.355 0.25

Nakano (2015) | 3.175 1.191 8.459 0.22

New Zealand Surv | 0.001 0.000 7.5e+34 0.00

SIREVA (Brasil) | 0.240 0.034 1.707 0.15

SIREVA (Chile) | 0.000 0.000 3.5e+34 0.00

SIREVA (Colombia) | 0.481 0.068 3.413 0.15

SIREVA (Costa Rica) | 0.001 0.000 1.5e+35 0.00

SIREVA (Ecuador) | 0.001 0.000 9.4e+34 0.00

SIREVA (El Salvador) | 0.002 0.000 1.9e+35 0.00

SIREVA (Mexico) | 6.667 3.178 13.984 0.24

SIREVA (Panama) | 0.001 0.000 8.6e+34 0.00

SIREVA (Peru) | 0.002 0.000 2.5e+35 0.00

SIREVA (Uruguay) | 0.001 0.000 6.1e+34 0.00

Scotland Surv | 1.456 0.470 4.515 0.21

Shibl (2012) | 0.000 0.000 5.4e+34 0.00

Singapore Surv | 0.001 0.000 9.0e+34 0.00

Steens (2013) | 0.001 0.000 1.2e+35 0.00

Varon (2015) | 2.210 0.829 5.888 0.22

Waight (2015) | 0.810 0.203 3.238 0.19

van der Linden (2015 | 1.235 0.589 2.590 0.24

von Gottberg (2013) | 0.000 0.000 6.9e+33 0.00

Sub-total |

D+L pooled ES | 2.599 1.764 3.829 2.74

I-V pooled ES | 3.031 2.413 3.806

---------------------+---------------------------------------------------

8

Al-Sheikh (2014) | 1.282 0.181 9.102 0.15

Australia Surv | 0.000 0.000 3.2e+34 0.00

Bruce (2015) | 0.001 0.000 1.1e+35 0.00

Demczuk (2013) | 1.693 1.021 2.808 0.25

Guevara (2014) | 0.002 0.000 2.3e+35 0.00

Kaplan (2013) | 0.353 0.050 2.509 0.15

Knol (2015) | 3.509 0.878 14.030 0.19

Moore (2014) | 6.250 2.016 19.379 0.21

Moore (2015) | 0.000 0.000 3.3e+34 0.00

Nakano (2015) | 0.000 0.000 4.6e+34 0.00

New Zealand Surv | 3.846 1.240 11.926 0.21

SIREVA (Brasil) | 0.962 0.361 2.562 0.22

SIREVA (Chile) | 0.595 0.084 4.226 0.15

SIREVA (Colombia) | 1.442 0.465 4.472 0.21

SIREVA (Costa Rica) | 0.001 0.000 1.5e+35 0.00

SIREVA (Ecuador) | 0.001 0.000 9.4e+34 0.00

SIREVA (El Salvador) | 3.226 0.454 22.901 0.15

SIREVA (Mexico) | 0.000 0.000 5.6e+34 0.00

SIREVA (Panama) | 0.001 0.000 8.6e+34 0.00

SIREVA (Peru) | 0.002 0.000 2.5e+35 0.00

SIREVA (Uruguay) | 0.001 0.000 6.1e+34 0.00

Scotland Surv | 0.971 0.243 3.882 0.19

Shibl (2012) | 0.000 0.000 5.4e+34 0.00

Singapore Surv | 0.001 0.000 9.0e+34 0.00

Steens (2013) | 0.001 0.000 1.2e+35 0.00

Varon (2015) | 0.000 0.000 3.2e+34 0.00

Waight (2015) | 6.073 3.661 10.073 0.25

van der Linden (2015 | 1.235 0.589 2.590 0.24

von Gottberg (2013) | 8.304 6.569 10.496 0.26

Sub-total |

D+L pooled ES | 2.233 1.313 3.797 2.85

I-V pooled ES | 4.672 3.929 5.556

---------------------+---------------------------------------------------

23A

Al-Sheikh (2014) | 2.564 0.641 10.253 0.19

Australia Surv | 0.543 0.077 3.858 0.15

Bruce (2015) | 1.923 0.271 13.653 0.15

Demczuk (2013) | 2.144 1.368 3.362 0.25

Guevara (2014) | 0.002 0.000 2.3e+35 0.00

Kaplan (2013) | 2.473 1.179 5.188 0.24

Knol (2015) | 1.754 0.247 12.455 0.15

Moore (2014) | 0.001 0.000 1.2e+35 0.00

Moore (2015) | 0.000 0.000 3.3e+34 0.00

Nakano (2015) | 2.381 0.768 7.382 0.21

New Zealand Surv | 3.846 1.240 11.926 0.21

SIREVA (Brasil) | 0.481 0.120 1.922 0.19

SIREVA (Chile) | 0.000 0.000 3.5e+34 0.00

SIREVA (Colombia) | 2.885 1.296 6.421 0.23

SIREVA (Costa Rica) | 0.001 0.000 1.5e+35 0.00

SIREVA (Ecuador) | 0.001 0.000 9.4e+34 0.00

SIREVA (El Salvador) | 6.452 1.613 25.797 0.19

SIREVA (Mexico) | 0.952 0.134 6.761 0.15

SIREVA (Panama) | 0.001 0.000 8.6e+34 0.00

SIREVA (Peru) | 0.002 0.000 2.5e+35 0.00

SIREVA (Uruguay) | 1.042 0.147 7.395 0.15

Scotland Surv | 0.000 0.000 2.8e+34 0.00

Shibl (2012) | 0.000 0.000 5.4e+34 0.00

Singapore Surv | 0.001 0.000 9.0e+34 0.00

Steens (2013) | 0.001 0.000 1.2e+35 0.00

Varon (2015) | 2.210 0.829 5.888 0.22

Waight (2015) | 1.215 0.392 3.766 0.21

van der Linden (2015 | 1.411 0.706 2.821 0.24

von Gottberg (2013) | 0.000 0.000 6.9e+33 0.00

Sub-total |

D+L pooled ES | 2.021 1.582 2.582 3.15

I-V pooled ES | 2.021 1.582 2.582

---------------------+---------------------------------------------------

9N

Al-Sheikh (2014) | 0.001 0.000 7.5e+34 0.00

Australia Surv | 0.543 0.077 3.858 0.15

Bruce (2015) | 0.001 0.000 1.1e+35 0.00

Demczuk (2013) | 0.677 0.304 1.507 0.23

Guevara (2014) | 0.002 0.000 2.3e+35 0.00

Kaplan (2013) | 1.060 0.342 3.287 0.21

Knol (2015) | 1.754 0.247 12.455 0.15

Moore (2014) | 0.001 0.000 1.2e+35 0.00

Moore (2015) | 0.000 0.000 3.3e+34 0.00

Nakano (2015) | 0.000 0.000 4.6e+34 0.00

New Zealand Surv | 2.564 0.641 10.253 0.19

SIREVA (Brasil) | 1.202 0.500 2.888 0.23

SIREVA (Chile) | 0.000 0.000 3.5e+34 0.00

SIREVA (Colombia) | 0.000 0.000 2.8e+34 0.00

SIREVA (Costa Rica) | 2.632 0.371 18.682 0.15

SIREVA (Ecuador) | 0.001 0.000 9.4e+34 0.00

SIREVA (El Salvador) | 0.002 0.000 1.9e+35 0.00

SIREVA (Mexico) | 0.000 0.000 5.6e+34 0.00

SIREVA (Panama) | 0.001 0.000 8.6e+34 0.00

SIREVA (Peru) | 0.002 0.000 2.5e+35 0.00

SIREVA (Uruguay) | 1.042 0.147 7.395 0.15

Scotland Surv | 0.000 0.000 2.8e+34 0.00

Shibl (2012) | 0.000 0.000 5.4e+34 0.00

Singapore Surv | 0.001 0.000 9.0e+34 0.00

Steens (2013) | 0.001 0.000 1.2e+35 0.00

Varon (2015) | 0.000 0.000 3.2e+34 0.00

Waight (2015) | 0.405 0.057 2.874 0.15

van der Linden (2015 | 1.411 0.706 2.821 0.24

von Gottberg (2013) | 1.779 1.073 2.952 0.25

Sub-total |

D+L pooled ES | 1.314 0.978 1.766 2.11

I-V pooled ES | 1.314 0.978 1.766

---------------------+---------------------------------------------------

38

Al-Sheikh (2014) | 0.001 0.000 7.5e+34 0.00

Australia Surv | 0.543 0.077 3.858 0.15

Bruce (2015) | 0.001 0.000 1.1e+35 0.00

Demczuk (2013) | 2.822 1.907 4.176 0.26

Guevara (2014) | 0.002 0.000 2.3e+35 0.00

Kaplan (2013) | 0.000 0.000 2.1e+34 0.00

Knol (2015) | 1.754 0.247 12.455 0.15

Moore (2014) | 2.083 0.293 14.790 0.15

Moore (2015) | 9.040 5.538 14.755 0.25

Nakano (2015) | 0.794 0.112 5.634 0.15

New Zealand Surv | 0.001 0.000 7.5e+34 0.00

SIREVA (Brasil) | 0.000 0.000 1.4e+34 0.00

SIREVA (Chile) | 1.786 0.576 5.537 0.21

SIREVA (Colombia) | 0.000 0.000 2.8e+34 0.00

SIREVA (Costa Rica) | 0.001 0.000 1.5e+35 0.00

SIREVA (Ecuador) | 0.001 0.000 9.4e+34 0.00

SIREVA (El Salvador) | 0.002 0.000 1.9e+35 0.00

SIREVA (Mexico) | 0.000 0.000 5.6e+34 0.00

SIREVA (Panama) | 0.001 0.000 8.6e+34 0.00

SIREVA (Peru) | 0.002 0.000 2.5e+35 0.00

SIREVA (Uruguay) | 0.001 0.000 6.1e+34 0.00

Scotland Surv | 0.971 0.243 3.882 0.19

Shibl (2012) | 0.000 0.000 5.4e+34 0.00

Singapore Surv | 0.001 0.000 9.0e+34 0.00

Steens (2013) | 0.001 0.000 1.2e+35 0.00

Varon (2015) | 2.762 1.150 6.637 0.23

Waight (2015) | 3.239 1.620 6.477 0.24

van der Linden (2015 | 6.526 4.728 9.007 0.26

von Gottberg (2013) | 0.000 0.000 6.9e+33 0.00

Sub-total |

D+L pooled ES | 3.427 2.436 4.820 2.25

I-V pooled ES | 4.354 3.579 5.296

---------------------+---------------------------------------------------

15BC

Al-Sheikh (2014) | 0.001 0.000 7.5e+34 0.00

Australia Surv | 6.522 3.704 11.484 0.25

Bruce (2015) | 13.462 6.417 28.237 0.24

Demczuk (2013) | 5.643 4.277 7.446 0.26

Guevara (2014) | 0.002 0.000 2.3e+35 0.00

Kaplan (2013) | 4.947 2.930 8.353 0.25

Knol (2015) | 8.772 3.651 21.075 0.23

Moore (2014) | 14.583 6.952 30.591 0.24

Moore (2015) | 14.124 9.544 20.903 0.26

Nakano (2015) | 0.000 0.000 4.6e+34 0.00

New Zealand Surv | 5.128 1.925 13.664 0.22

SIREVA (Brasil) | 1.923 0.962 3.845 0.24

SIREVA (Chile) | 2.381 0.894 6.344 0.22

SIREVA (Colombia) | 2.885 1.296 6.421 0.23

SIREVA (Costa Rica) | 0.001 0.000 1.5e+35 0.00

SIREVA (Ecuador) | 0.001 0.000 9.4e+34 0.00

SIREVA (El Salvador) | 0.002 0.000 1.9e+35 0.00

SIREVA (Mexico) | 5.714 2.567 12.719 0.23

SIREVA (Panama) | 1.471 0.207 10.440 0.15

SIREVA (Peru) | 0.002 0.000 2.5e+35 0.00

SIREVA (Uruguay) | 0.001 0.000 6.1e+34 0.00

Scotland Surv | 5.825 3.308 10.257 0.25

Shibl (2012) | 0.000 0.000 5.4e+34 0.00

Singapore Surv | 0.001 0.000 9.0e+34 0.00

Steens (2013) | 0.001 0.000 1.2e+35 0.00

Varon (2015) | 0.000 0.000 3.2e+34 0.00

Waight (2015) | 0.000 0.000 2.4e+34 0.00

van der Linden (2015 | 9.877 7.601 12.834 0.26

von Gottberg (2013) | 4.270 3.080 5.920 0.26

Sub-total |

D+L pooled ES | 6.060 4.674 7.857 3.80

I-V pooled ES | 6.653 5.882 7.526

---------------------+---------------------------------------------------

Study | ES [95% Conf. Interval] % Weight

---------------------+---------------------------------------------------

12F Africa

von Gottberg (2013) | 4.033 2.882 5.645 0.26

Sub-total |

D+L pooled ES | 4.033 2.882 5.645 0.26

---------------------+---------------------------------------------------

3 Africa

von Gottberg (2013) | 2.017 1.254 3.244 0.25

Sub-total |

D+L pooled ES | 2.017 1.254 3.244 0.25

---------------------+---------------------------------------------------

11A Africa

von Gottberg (2013) | 0.000 0.000 6.9e+33 0.00

Sub-total |

D+L pooled ES | 0.000 0.000 6.9e+33 0.00

---------------------+---------------------------------------------------

19A Africa

von Gottberg (2013) | 11.388 9.323 13.910 0.26

Sub-total |

D+L pooled ES | 11.388 9.323 13.910 0.26

---------------------+---------------------------------------------------

38 Africa

von Gottberg (2013) | 0.000 0.000 6.9e+33 0.00

Sub-total |

D+L pooled ES | 0.000 0.000 6.9e+33 0.00

---------------------+---------------------------------------------------

15C Africa

von Gottberg (2013) | 0.000 0.000 6.9e+33 0.00

Sub-total |

D+L pooled ES | 0.000 0.000 6.9e+33 0.00

---------------------+---------------------------------------------------

33F Africa

von Gottberg (2013) | 0.000 0.000 6.9e+33 0.00

Sub-total |

D+L pooled ES | 0.000 0.000 6.9e+33 0.00

---------------------+---------------------------------------------------

15B Africa

von Gottberg (2013) | 4.270 3.080 5.920 0.26

Sub-total |

D+L pooled ES | 4.270 3.080 5.920 0.26

---------------------+---------------------------------------------------

8 Africa

von Gottberg (2013) | 8.304 6.569 10.496 0.26

Sub-total |

D+L pooled ES | 8.304 6.569 10.496 0.26

---------------------+---------------------------------------------------

9N Africa

von Gottberg (2013) | 1.779 1.073 2.952 0.25

Sub-total |

D+L pooled ES | 1.779 1.073 2.952 0.25

---------------------+---------------------------------------------------

10A Africa

von Gottberg (2013) | 0.000 0.000 6.9e+33 0.00

Sub-total |

D+L pooled ES | 0.000 0.000 6.9e+33 0.00

---------------------+---------------------------------------------------

6A Africa

von Gottberg (2013) | 8.541 6.779 10.760 0.26

Sub-total |

D+L pooled ES | 8.541 6.779 10.760 0.26

---------------------+---------------------------------------------------

15BC Africa

von Gottberg (2013) | 4.270 3.080 5.920 0.26

Sub-total |

D+L pooled ES | 4.270 3.080 5.920 0.26

---------------------+---------------------------------------------------

23B Africa

von Gottberg (2013) | 0.000 0.000 6.9e+33 0.00

Sub-total |

D+L pooled ES | 0.000 0.000 6.9e+33 0.00

---------------------+---------------------------------------------------

pcv10-pcv7 Africa

von Gottberg (2013) | 9.846 7.940 12.209 0.26

Sub-total |

D+L pooled ES | 9.846 7.940 12.209 0.26

---------------------+---------------------------------------------------

pcv7 Africa

von Gottberg (2013) | 25.504 22.313 29.152 0.26

Sub-total |

D+L pooled ES | 25.504 22.313 29.152 0.26

---------------------+---------------------------------------------------

15A Africa

von Gottberg (2013) | 0.000 0.000 6.9e+33 0.00

Sub-total |

D+L pooled ES | 0.000 0.000 6.9e+33 0.00

---------------------+---------------------------------------------------

23A Africa

von Gottberg (2013) | 0.000 0.000 6.9e+33 0.00

Sub-total |

D+L pooled ES | 0.000 0.000 6.9e+33 0.00

---------------------+---------------------------------------------------

6C Africa

von Gottberg (2013) | 0.000 0.000 6.9e+33 0.00

Sub-total |

D+L pooled ES | 0.000 0.000 6.9e+33 0.00

---------------------+---------------------------------------------------

22F Africa

von Gottberg (2013) | 0.000 0.000 6.9e+33 0.00

Sub-total |

D+L pooled ES | 0.000 0.000 6.9e+33 0.00

---------------------+---------------------------------------------------

pcv13 Africa

von Gottberg (2013) | 57.295 52.407 62.640 0.26

Sub-total |

D+L pooled ES | 57.295 52.407 62.640 0.26

---------------------+---------------------------------------------------

24F Africa

von Gottberg (2013) | 0.000 0.000 6.9e+33 0.00

Sub-total |

D+L pooled ES | 0.000 0.000 6.9e+33 0.00

---------------------+---------------------------------------------------

35B Africa

von Gottberg (2013) | 0.000 0.000 6.9e+33 0.00

Sub-total |

D+L pooled ES | 0.000 0.000 6.9e+33 0.00

---------------------+---------------------------------------------------

non-pcv Africa

von Gottberg (2013) | 42.705 38.513 47.352 0.26

Sub-total |

D+L pooled ES | 42.705 38.513 47.352 0.26

---------------------+---------------------------------------------------

33F Europe

Guevara (2014) | 4.000 0.563 28.397 0.15

Varon (2015) | 3.315 1.489 7.379 0.23

van der Linden (2015 | 3.175 2.000 5.039 0.25

Knol (2015) | 10.526 4.729 23.431 0.23

Steens (2013) | 4.255 1.064 17.015 0.19

Moore (2014) | 6.250 2.016 19.379 0.21

Scotland Surv | 2.913 1.309 6.483 0.23

Waight (2015) | 8.097 5.224 12.551 0.26

Sub-total |

D+L pooled ES | 4.908 3.263 7.382 1.77

---------------------+---------------------------------------------------

15C Europe

Guevara (2014) | 0.002 0.000 2.3e+35 0.00

Varon (2015) | 0.000 0.000 3.2e+34 0.00

van der Linden (2015 | 5.996 4.285 8.392 0.26

Knol (2015) | 7.018 2.634 18.698 0.22

Steens (2013) | 0.001 0.000 1.2e+35 0.00

Moore (2014) | 10.417 4.336 25.027 0.23

Scotland Surv | 2.427 1.010 5.831 0.23

Waight (2015) | 0.000 0.000 2.4e+34 0.00

Sub-total |

D+L pooled ES | 5.854 4.412 7.769 0.94

---------------------+---------------------------------------------------

pcv7 Europe

Guevara (2014) | 4.000 0.563 28.397 0.15

Varon (2015) | 5.525 2.973 10.268 0.25

van der Linden (2015 | 4.938 3.410 7.152 0.26

Knol (2015) | 0.001 0.000 1.0e+35 0.00

Steens (2013) | 2.128 0.300 15.105 0.15

Moore (2014) | 6.250 2.016 19.379 0.21

Scotland Surv | 1.942 0.729 5.174 0.22

Waight (2015) | 2.834 1.351 5.945 0.24

Sub-total |

D+L pooled ES | 4.350 3.332 5.680 1.48

---------------------+---------------------------------------------------

19A Europe

Guevara (2014) | 24.000 10.782 53.422 0.23

Varon (2015) | 8.840 5.415 14.429 0.25

van der Linden (2015 | 11.817 9.300 15.014 0.26

Knol (2015) | 19.298 10.687 34.847 0.25

Steens (2013) | 21.277 11.448 39.544 0.25

Moore (2014) | 16.667 8.335 33.327 0.24

Scotland Surv | 5.825 3.308 10.257 0.25

Waight (2015) | 2.834 1.351 5.945 0.24

Sub-total |

D+L pooled ES | 11.347 7.572 17.004 1.97

---------------------+---------------------------------------------------

15A Europe

Guevara (2014) | 0.002 0.000 2.3e+35 0.00

Varon (2015) | 7.182 4.170 12.369 0.25

van der Linden (2015 | 1.235 0.589 2.590 0.24

Knol (2015) | 0.001 0.000 1.0e+35 0.00

Steens (2013) | 0.001 0.000 1.2e+35 0.00

Moore (2014) | 0.001 0.000 1.2e+35 0.00

Scotland Surv | 2.913 1.309 6.483 0.23

Waight (2015) | 7.287 4.591 11.567 0.25

Sub-total |

D+L pooled ES | 3.895 1.920 7.901 0.98

---------------------+---------------------------------------------------

23B Europe

Guevara (2014) | 0.002 0.000 2.3e+35 0.00

Varon (2015) | 1.105 0.276 4.418 0.19

van der Linden (2015 | 4.762 3.266 6.944 0.26

Knol (2015) | 0.001 0.000 1.0e+35 0.00

Steens (2013) | 0.001 0.000 1.2e+35 0.00

Moore (2014) | 2.083 0.293 14.790 0.15

Scotland Surv | 2.427 1.010 5.831 0.23

Waight (2015) | 7.287 4.591 11.567 0.25

Sub-total |

D+L pooled ES | 4.107 2.608 6.468 1.08

---------------------+---------------------------------------------------

23A Europe

Guevara (2014) | 0.002 0.000 2.3e+35 0.00

Varon (2015) | 2.210 0.829 5.888 0.22

van der Linden (2015 | 1.411 0.706 2.821 0.24

Knol (2015) | 1.754 0.247 12.455 0.15

Steens (2013) | 0.001 0.000 1.2e+35 0.00

Moore (2014) | 0.001 0.000 1.2e+35 0.00

Scotland Surv | 0.000 0.000 2.8e+34 0.00

Waight (2015) | 1.215 0.392 3.766 0.21

Sub-total |

D+L pooled ES | 1.554 0.952 2.537 0.83

---------------------+---------------------------------------------------

non-pcv Europe

Guevara (2014) | 48.000 27.259 84.521 0.25

Varon (2015) | 82.320 70.109 96.659 0.26

van der Linden (2015 | 64.550 58.265 71.514 0.26

Knol (2015) | 78.947 58.945 105.738 0.26

Steens (2013) | 44.681 29.132 68.529 0.26

Moore (2014) | 62.500 43.699 89.390 0.26

Scotland Surv | 80.097 68.762 93.300 0.26

Waight (2015) | 85.830 75.020 98.198 0.26

Sub-total |

D+L pooled ES | 71.936 63.085 82.028 2.08

---------------------+---------------------------------------------------

22F Europe

Guevara (2014) | 4.000 0.563 28.397 0.15

Varon (2015) | 4.972 2.587 9.557 0.24

van der Linden (2015 | 3.527 2.276 5.467 0.26

Knol (2015) | 5.263 1.697 16.319 0.21

Steens (2013) | 4.255 1.064 17.015 0.19

Scotland Surv | 4.369 2.273 8.397 0.24

Waight (2015) | 9.312 6.188 14.013 0.26

Sub-total |

D+L pooled ES | 5.178 3.570 7.510 1.55

---------------------+---------------------------------------------------

35B Europe

Guevara (2014) | 0.002 0.000 2.3e+35 0.00

Varon (2015) | 2.210 0.829 5.888 0.22

van der Linden (2015 | 1.235 0.589 2.590 0.24

Knol (2015) | 1.754 0.247 12.455 0.15

Steens (2013) | 0.001 0.000 1.2e+35 0.00

Moore (2014) | 0.001 0.000 1.2e+35 0.00

Scotland Surv | 1.456 0.470 4.515 0.21

Waight (2015) | 0.810 0.203 3.238 0.19

Sub-total |

D+L pooled ES | 1.415 0.880 2.277 1.01

---------------------+---------------------------------------------------

pcv10-pcv7 Europe

Guevara (2014) | 12.000 3.870 37.208 0.21

Varon (2015) | 0.000 0.000 3.2e+34 0.00

van der Linden (2015 | 12.346 9.767 15.605 0.26

Knol (2015) | 1.754 0.247 12.455 0.15

Steens (2013) | 25.532 14.500 44.958 0.25

Moore (2014) | 10.417 4.336 25.027 0.23

Scotland Surv | 8.252 5.130 13.275 0.25

Waight (2015) | 5.263 3.056 9.064 0.25

Sub-total |

D+L pooled ES | 10.124 6.640 15.437 1.61

---------------------+---------------------------------------------------

6C Europe

Guevara (2014) | 4.000 0.563 28.397 0.15

Varon (2015) | 0.552 0.078 3.922 0.15

van der Linden (2015 | 1.058 0.475 2.355 0.23

Knol (2015) | 1.754 0.247 12.455 0.15

Steens (2013) | 8.511 3.194 22.676 0.22

Moore (2014) | 2.083 0.293 14.790 0.15

Scotland Surv | 0.485 0.068 3.446 0.15

Waight (2015) | 0.810 0.203 3.238 0.19

Sub-total |

D+L pooled ES | 1.637 0.734 3.648 1.40

---------------------+---------------------------------------------------

12F Europe

Guevara (2014) | 8.000 2.001 31.988 0.19

Varon (2015) | 8.840 5.415 14.429 0.25

van der Linden (2015 | 4.056 2.696 6.104 0.26

Knol (2015) | 3.509 0.878 14.030 0.19

Steens (2013) | 0.001 0.000 1.2e+35 0.00

Moore (2014) | 6.250 2.016 19.379 0.21

Scotland Surv | 4.854 2.612 9.022 0.25

Waight (2015) | 6.073 3.661 10.073 0.25

Sub-total |

D+L pooled ES | 5.583 4.424 7.044 1.60

---------------------+---------------------------------------------------

6A Europe

Guevara (2014) | 0.002 0.000 2.3e+35 0.00

Varon (2015) | 0.552 0.078 3.922 0.15

van der Linden (2015 | 1.058 0.475 2.355 0.23

Knol (2015) | 0.001 0.000 1.0e+35 0.00

Steens (2013) | 0.001 0.000 1.2e+35 0.00

Moore (2014) | 0.001 0.000 1.2e+35 0.00

Scotland Surv | 0.000 0.000 2.8e+34 0.00

Waight (2015) | 0.000 0.000 2.4e+34 0.00

Sub-total |

D+L pooled ES | 0.961 0.458 2.016 0.39

---------------------+---------------------------------------------------

11A Europe

Guevara (2014) | 0.002 0.000 2.3e+35 0.00

Varon (2015) | 1.105 0.276 4.418 0.19

van der Linden (2015 | 1.940 1.074 3.503 0.25

Knol (2015) | 0.001 0.000 1.0e+35 0.00

Steens (2013) | 2.128 0.300 15.105 0.15

Moore (2014) | 2.083 0.293 14.790 0.15

Scotland Surv | 0.971 0.243 3.882 0.19

Waight (2015) | 1.619 0.608 4.315 0.22

Sub-total |

D+L pooled ES | 1.676 1.093 2.570 1.15

---------------------+---------------------------------------------------

3 Europe

Guevara (2014) | 12.000 3.870 37.208 0.21

Varon (2015) | 2.762 1.150 6.637 0.23

van der Linden (2015 | 5.291 3.699 7.567 0.26

Knol (2015) | 0.001 0.000 1.0e+35 0.00

Steens (2013) | 6.383 2.059 19.791 0.21

Moore (2014) | 4.167 1.042 16.661 0.19

Scotland Surv | 3.883 1.942 7.766 0.24

Waight (2015) | 3.239 1.620 6.477 0.24

Sub-total |

D+L pooled ES | 4.690 3.634 6.053 1.59

---------------------+---------------------------------------------------

38 Europe

Guevara (2014) | 0.002 0.000 2.3e+35 0.00

Varon (2015) | 2.762 1.150 6.637 0.23

van der Linden (2015 | 6.526 4.728 9.007 0.26

Knol (2015) | 1.754 0.247 12.455 0.15

Steens (2013) | 0.001 0.000 1.2e+35 0.00

Moore (2014) | 2.083 0.293 14.790 0.15

Scotland Surv | 0.971 0.243 3.882 0.19

Waight (2015) | 3.239 1.620 6.477 0.24

Sub-total |

D+L pooled ES | 3.200 1.821 5.622 1.22

---------------------+---------------------------------------------------

24F Europe

Guevara (2014) | 4.000 0.563 28.397 0.15

Varon (2015) | 19.890 14.347 27.574 0.26

van der Linden (2015 | 8.818 6.684 11.635 0.26

Knol (2015) | 1.754 0.247 12.455 0.15

Steens (2013) | 2.128 0.300 15.105 0.15

Moore (2014) | 2.083 0.293 14.790 0.15

Scotland Surv | 0.485 0.068 3.446 0.15

Waight (2015) | 11.336 7.827 16.418 0.26

Sub-total |

D+L pooled ES | 6.707 3.790 11.867 1.53

---------------------+---------------------------------------------------

15BC Europe

Guevara (2014) | 0.002 0.000 2.3e+35 0.00

Varon (2015) | 0.000 0.000 3.2e+34 0.00

van der Linden (2015 | 9.877 7.601 12.834 0.26

Knol (2015) | 8.772 3.651 21.075 0.23

Steens (2013) | 0.001 0.000 1.2e+35 0.00

Moore (2014) | 14.583 6.952 30.591 0.24

Scotland Surv | 5.825 3.308 10.257 0.25

Waight (2015) | 0.000 0.000 2.4e+34 0.00

Sub-total |

D+L pooled ES | 9.369 7.525 11.664 0.98

---------------------+---------------------------------------------------

pcv13 Europe

Guevara (2014) | 52.000 30.194 89.555 0.25

Varon (2015) | 17.680 12.502 25.000 0.26

van der Linden (2015 | 35.450 30.873 40.706 0.26

Knol (2015) | 21.053 11.956 37.071 0.25

Steens (2013) | 55.319 37.665 81.248 0.26

Moore (2014) | 37.500 23.626 59.520 0.25

Scotland Surv | 19.903 14.655 27.031 0.26

Waight (2015) | 14.170 10.174 19.736 0.26

Sub-total |

D+L pooled ES | 27.901 19.935 39.051 2.05

---------------------+---------------------------------------------------

15B Europe

Guevara (2014) | 0.002 0.000 2.3e+35 0.00

Varon (2015) | 0.000 0.000 3.2e+34 0.00

van der Linden (2015 | 3.880 2.555 5.893 0.26

Knol (2015) | 1.754 0.247 12.455 0.15

Steens (2013) | 0.001 0.000 1.2e+35 0.00

Moore (2014) | 4.167 1.042 16.661 0.19

Scotland Surv | 3.398 1.620 7.128 0.24

Waight (2015) | 0.000 0.000 2.4e+34 0.00

Sub-total |

D+L pooled ES | 3.691 2.610 5.220 0.84

---------------------+---------------------------------------------------

9N Europe

Guevara (2014) | 0.002 0.000 2.3e+35 0.00

Varon (2015) | 0.000 0.000 3.2e+34 0.00

van der Linden (2015 | 1.411 0.706 2.821 0.24

Knol (2015) | 1.754 0.247 12.455 0.15

Steens (2013) | 0.001 0.000 1.2e+35 0.00

Moore (2014) | 0.001 0.000 1.2e+35 0.00

Scotland Surv | 0.000 0.000 2.8e+34 0.00

Waight (2015) | 0.405 0.057 2.874 0.15

Sub-total |

D+L pooled ES | 1.270 0.684 2.361 0.54

---------------------+---------------------------------------------------

10A Europe

Guevara (2014) | 0.002 0.000 2.3e+35 0.00

Varon (2015) | 7.735 4.581 13.060 0.25

van der Linden (2015 | 6.878 5.025 9.414 0.26

Knol (2015) | 28.070 17.197 45.819 0.25

Steens (2013) | 4.255 1.064 17.015 0.19

Moore (2014) | 8.333 3.128 22.204 0.22

Scotland Surv | 2.913 1.309 6.483 0.23

Waight (2015) | 2.834 1.351 5.945 0.24

Sub-total |

D+L pooled ES | 6.746 3.679 12.369 1.65

---------------------+---------------------------------------------------

8 Europe

Guevara (2014) | 0.002 0.000 2.3e+35 0.00

Varon (2015) | 0.000 0.000 3.2e+34 0.00

van der Linden (2015 | 1.235 0.589 2.590 0.24

Knol (2015) | 3.509 0.878 14.030 0.19

Steens (2013) | 0.001 0.000 1.2e+35 0.00

Moore (2014) | 6.250 2.016 19.379 0.21

Scotland Surv | 0.971 0.243 3.882 0.19

Waight (2015) | 6.073 3.661 10.073 0.25

Sub-total |

D+L pooled ES | 2.891 1.339 6.241 1.09

---------------------+---------------------------------------------------

15A Latin America

SIREVA (Brasil) | 0.721 0.233 2.236 0.21

SIREVA (Chile) | 0.595 0.084 4.226 0.15

SIREVA (Colombia) | 0.962 0.240 3.845 0.19

SIREVA (Costa Rica) | 5.263 1.316 21.045 0.19

SIREVA (Ecuador) | 1.613 0.227 11.451 0.15

SIREVA (El Salvador) | 0.002 0.000 1.9e+35 0.00

SIREVA (Mexico) | 0.000 0.000 5.6e+34 0.00

SIREVA (Panama) | 2.941 0.736 11.760 0.19

SIREVA (Peru) | 0.002 0.000 2.5e+35 0.00

SIREVA (Uruguay) | 1.042 0.147 7.395 0.15

Sub-total |

D+L pooled ES | 1.444 0.820 2.543 1.24

---------------------+---------------------------------------------------

12F Latin America

SIREVA (Brasil) | 3.365 1.993 5.682 0.25

SIREVA (Chile) | 3.571 1.604 7.950 0.23

SIREVA (Colombia) | 0.000 0.000 2.8e+34 0.00

SIREVA (Costa Rica) | 0.001 0.000 1.5e+35 0.00

SIREVA (Ecuador) | 0.001 0.000 9.4e+34 0.00

SIREVA (El Salvador) | 0.002 0.000 1.9e+35 0.00

SIREVA (Mexico) | 0.952 0.134 6.761 0.15

SIREVA (Panama) | 0.001 0.000 8.6e+34 0.00

SIREVA (Peru) | 4.348 0.612 30.867 0.15

SIREVA (Uruguay) | 7.292 3.476 15.295 0.24

Sub-total |

D+L pooled ES | 3.963 2.754 5.703 1.03

---------------------+---------------------------------------------------

35B Latin America

SIREVA (Brasil) | 0.240 0.034 1.707 0.15

SIREVA (Chile) | 0.000 0.000 3.5e+34 0.00

SIREVA (Colombia) | 0.481 0.068 3.413 0.15

SIREVA (Costa Rica) | 0.001 0.000 1.5e+35 0.00

SIREVA (Ecuador) | 0.001 0.000 9.4e+34 0.00

SIREVA (El Salvador) | 0.002 0.000 1.9e+35 0.00

SIREVA (Mexico) | 6.667 3.178 13.984 0.24

SIREVA (Panama) | 0.001 0.000 8.6e+34 0.00

SIREVA (Peru) | 0.002 0.000 2.5e+35 0.00

SIREVA (Uruguay) | 0.001 0.000 6.1e+34 0.00

Sub-total |

D+L pooled ES | 1.194 0.223 6.402 0.54

---------------------+---------------------------------------------------

15BC Latin America

SIREVA (Brasil) | 1.923 0.962 3.845 0.24

SIREVA (Chile) | 2.381 0.894 6.344 0.22

SIREVA (Colombia) | 2.885 1.296 6.421 0.23

SIREVA (Costa Rica) | 0.001 0.000 1.5e+35 0.00

SIREVA (Ecuador) | 0.001 0.000 9.4e+34 0.00

SIREVA (El Salvador) | 0.002 0.000 1.9e+35 0.00

SIREVA (Mexico) | 5.714 2.567 12.719 0.23

SIREVA (Panama) | 1.471 0.207 10.440 0.15

SIREVA (Peru) | 0.002 0.000 2.5e+35 0.00

SIREVA (Uruguay) | 0.001 0.000 6.1e+34 0.00

Sub-total |

D+L pooled ES | 2.816 1.903 4.167 1.08

---------------------+---------------------------------------------------

6A Latin America

SIREVA (Brasil) | 5.529 3.674 8.320 0.26

SIREVA (Chile) | 7.143 4.056 12.578 0.25

SIREVA (Colombia) | 4.327 2.251 8.316 0.24

SIREVA (Costa Rica) | 0.001 0.000 1.5e+35 0.00

SIREVA (Ecuador) | 6.452 2.421 17.190 0.22

SIREVA (El Salvador) | 0.002 0.000 1.9e+35 0.00

SIREVA (Mexico) | 1.905 0.476 7.616 0.19

SIREVA (Panama) | 5.882 2.208 15.673 0.22

SIREVA (Peru) | 4.348 0.612 30.867 0.15

SIREVA (Uruguay) | 0.001 0.000 6.1e+34 0.00

Sub-total |

D+L pooled ES | 5.464 4.195 7.116 1.54

---------------------+---------------------------------------------------

pcv7 Latin America

SIREVA (Brasil) | 41.587 35.829 48.269 0.26

SIREVA (Chile) | 43.452 34.545 54.656 0.26

SIREVA (Colombia) | 40.865 33.039 50.546 0.26

SIREVA (Costa Rica) | 36.842 21.820 62.207 0.25

SIREVA (Ecuador) | 48.387 33.831 69.205 0.26

SIREVA (El Salvador) | 51.613 31.619 84.249 0.25

SIREVA (Mexico) | 17.143 10.801 27.209 0.25

SIREVA (Panama) | 10.294 4.907 21.593 0.24

SIREVA (Peru) | 34.783 17.394 69.553 0.24

SIREVA (Uruguay) | 13.542 7.863 23.322 0.25

Sub-total |

D+L pooled ES | 32.450 25.485 41.319 2.54

---------------------+---------------------------------------------------

22F Latin America

SIREVA (Brasil) | 1.923 0.962 3.845 0.24

SIREVA (Chile) | 2.976 1.239 7.151 0.23

SIREVA (Colombia) | 0.000 0.000 2.8e+34 0.00

SIREVA (Costa Rica) | 0.001 0.000 1.5e+35 0.00

SIREVA (Ecuador) | 0.001 0.000 9.4e+34 0.00

SIREVA (El Salvador) | 0.002 0.000 1.9e+35 0.00

SIREVA (Mexico) | 0.952 0.134 6.761 0.15

SIREVA (Panama) | 0.001 0.000 8.6e+34 0.00

SIREVA (Peru) | 0.002 0.000 2.5e+35 0.00

SIREVA (Uruguay) | 3.125 1.008 9.689 0.21

Sub-total |

D+L pooled ES | 2.283 1.419 3.672 0.83

---------------------+---------------------------------------------------

23B Latin America

SIREVA (Brasil) | 0.481 0.120 1.922 0.19

SIREVA (Chile) | 0.000 0.000 3.5e+34 0.00

SIREVA (Colombia) | 1.442 0.465 4.472 0.21

SIREVA (Costa Rica) | 0.001 0.000 1.5e+35 0.00

SIREVA (Ecuador) | 3.226 0.807 12.899 0.19

SIREVA (El Salvador) | 0.002 0.000 1.9e+35 0.00

SIREVA (Mexico) | 4.762 1.982 11.441 0.23

SIREVA (Panama) | 0.001 0.000 8.6e+34 0.00

SIREVA (Peru) | 4.348 0.612 30.867 0.15

SIREVA (Uruguay) | 0.001 0.000 6.1e+34 0.00

Sub-total |

D+L pooled ES | 2.372 1.377 4.085 0.97

---------------------+---------------------------------------------------

15C Latin America

SIREVA (Brasil) | 1.683 0.802 3.530 0.24

SIREVA (Chile) | 1.190 0.298 4.760 0.19

SIREVA (Colombia) | 0.000 0.000 2.8e+34 0.00

SIREVA (Costa Rica) | 0.001 0.000 1.5e+35 0.00

SIREVA (Ecuador) | 0.001 0.000 9.4e+34 0.00

SIREVA (El Salvador) | 0.002 0.000 1.9e+35 0.00

SIREVA (Mexico) | 0.952 0.134 6.761 0.15

SIREVA (Panama) | 1.471 0.207 10.440 0.15

SIREVA (Peru) | 0.002 0.000 2.5e+35 0.00

SIREVA (Uruguay) | 0.001 0.000 6.1e+34 0.00

Sub-total |

D+L pooled ES | 1.479 0.819 2.671 0.73

---------------------+---------------------------------------------------

6C Latin America

SIREVA (Brasil) | 2.885 1.638 5.079 0.25

SIREVA (Chile) | 2.381 0.894 6.344 0.22

SIREVA (Colombia) | 1.442 0.465 4.472 0.21

SIREVA (Costa Rica) | 0.001 0.000 1.5e+35 0.00

SIREVA (Ecuador) | 1.613 0.227 11.451 0.15

SIREVA (El Salvador) | 0.002 0.000 1.9e+35 0.00

SIREVA (Mexico) | 1.905 0.476 7.616 0.19

SIREVA (Panama) | 0.001 0.000 8.6e+34 0.00

SIREVA (Peru) | 4.348 0.612 30.867 0.15

SIREVA (Uruguay) | 0.001 0.000 6.1e+34 0.00

Sub-total |

D+L pooled ES | 2.439 1.620 3.670 1.17

---------------------+---------------------------------------------------

pcv13 Latin America

SIREVA (Brasil) | 68.750 61.226 77.198 0.26

SIREVA (Chile) | 72.619 60.811 86.719 0.26

SIREVA (Colombia) | 74.038 63.221 86.706 0.26

SIREVA (Costa Rica) | 76.316 53.033 109.820 0.26

SIREVA (Ecuador) | 80.645 61.122 106.404 0.26

SIREVA (El Salvador) | 74.194 49.303 111.650 0.26

SIREVA (Mexico) | 66.667 52.743 84.265 0.26

SIREVA (Panama) | 88.235 68.509 113.641 0.26

SIREVA (Peru) | 69.565 42.617 113.552 0.25

SIREVA (Uruguay) | 50.000 37.680 66.349 0.26

Sub-total |

D+L pooled ES | 71.242 65.944 76.967 2.60

---------------------+---------------------------------------------------

non-pcv Latin Americ

SIREVA (Brasil) | 31.250 26.314 37.111 0.26

SIREVA (Chile) | 27.381 20.509 36.556 0.26

SIREVA (Colombia) | 25.962 19.884 33.897 0.26

SIREVA (Costa Rica) | 23.684 12.323 45.520 0.24

SIREVA (Ecuador) | 19.355 10.992 34.081 0.25

SIREVA (El Salvador) | 25.806 12.906 51.603 0.24

SIREVA (Mexico) | 33.333 23.933 46.426 0.26

SIREVA (Panama) | 11.765 5.883 23.525 0.24

SIREVA (Peru) | 30.435 14.509 63.841 0.24

SIREVA (Uruguay) | 50.000 37.680 66.349 0.26

Sub-total |

D+L pooled ES | 28.482 23.355 34.736 2.52

---------------------+---------------------------------------------------

24F Latin America

SIREVA (Brasil) | 0.962 0.361 2.562 0.22

SIREVA (Chile) | 3.571 1.604 7.950 0.23

SIREVA (Colombia) | 0.962 0.240 3.845 0.19

SIREVA (Costa Rica) | 0.001 0.000 1.5e+35 0.00

SIREVA (Ecuador) | 4.839 1.561 15.003 0.21

SIREVA (El Salvador) | 0.002 0.000 1.9e+35 0.00

SIREVA (Mexico) | 0.000 0.000 5.6e+34 0.00

SIREVA (Panama) | 2.941 0.736 11.760 0.19

SIREVA (Peru) | 0.002 0.000 2.5e+35 0.00

SIREVA (Uruguay) | 3.125 1.008 9.689 0.21

Sub-total |

D+L pooled ES | 2.422 1.563 3.754 1.26

---------------------+---------------------------------------------------

8 Latin America

SIREVA (Brasil) | 0.962 0.361 2.562 0.22

SIREVA (Chile) | 0.595 0.084 4.226 0.15

SIREVA (Colombia) | 1.442 0.465 4.472 0.21

SIREVA (Costa Rica) | 0.001 0.000 1.5e+35 0.00

SIREVA (Ecuador) | 0.001 0.000 9.4e+34 0.00

SIREVA (El Salvador) | 3.226 0.454 22.901 0.15

SIREVA (Mexico) | 0.000 0.000 5.6e+34 0.00

SIREVA (Panama) | 0.001 0.000 8.6e+34 0.00

SIREVA (Peru) | 0.002 0.000 2.5e+35 0.00

SIREVA (Uruguay) | 0.001 0.000 6.1e+34 0.00

Sub-total |

D+L pooled ES | 1.191 0.620 2.289 0.73

---------------------+---------------------------------------------------

pcv10-pcv7 Latin Ame

SIREVA (Brasil) | 2.644 1.464 4.775 0.25

SIREVA (Chile) | 8.929 5.383 14.810 0.25

SIREVA (Colombia) | 12.981 8.902 18.929 0.26

SIREVA (Costa Rica) | 15.789 7.093 35.146 0.23

SIREVA (Ecuador) | 6.452 2.421 17.190 0.22

SIREVA (El Salvador) | 6.452 1.613 25.797 0.19

SIREVA (Mexico) | 4.762 1.982 11.441 0.23

SIREVA (Panama) | 60.294 44.395 81.887 0.26

SIREVA (Peru) | 4.348 0.612 30.867 0.15

SIREVA (Uruguay) | 20.833 13.441 32.292 0.26

Sub-total |

D+L pooled ES | 10.055 4.956 20.400 2.30

---------------------+---------------------------------------------------

23A Latin America

SIREVA (Brasil) | 0.481 0.120 1.922 0.19

SIREVA (Chile) | 0.000 0.000 3.5e+34 0.00

SIREVA (Colombia) | 2.885 1.296 6.421 0.23

SIREVA (Costa Rica) | 0.001 0.000 1.5e+35 0.00

SIREVA (Ecuador) | 0.001 0.000 9.4e+34 0.00

SIREVA (El Salvador) | 6.452 1.613 25.797 0.19

SIREVA (Mexico) | 0.952 0.134 6.761 0.15

SIREVA (Panama) | 0.001 0.000 8.6e+34 0.00

SIREVA (Peru) | 0.002 0.000 2.5e+35 0.00

SIREVA (Uruguay) | 1.042 0.147 7.395 0.15

Sub-total |

D+L pooled ES | 2.046 1.162 3.603 0.92

---------------------+---------------------------------------------------

9N Latin America

SIREVA (Brasil) | 1.202 0.500 2.888 0.23

SIREVA (Chile) | 0.000 0.000 3.5e+34 0.00

SIREVA (Colombia) | 0.000 0.000 2.8e+34 0.00

SIREVA (Costa Rica) | 2.632 0.371 18.682 0.15

SIREVA (Ecuador) | 0.001 0.000 9.4e+34 0.00

SIREVA (El Salvador) | 0.002 0.000 1.9e+35 0.00

SIREVA (Mexico) | 0.000 0.000 5.6e+34 0.00

SIREVA (Panama) | 0.001 0.000 8.6e+34 0.00

SIREVA (Peru) | 0.002 0.000 2.5e+35 0.00

SIREVA (Uruguay) | 1.042 0.147 7.395 0.15

Sub-total |

D+L pooled ES | 1.312 0.626 2.752 0.53

---------------------+---------------------------------------------------

10A Latin America

SIREVA (Brasil) | 0.962 0.361 2.562 0.22

SIREVA (Chile) | 0.595 0.084 4.226 0.15

SIREVA (Colombia) | 0.962 0.240 3.845 0.19

SIREVA (Costa Rica) | 2.632 0.371 18.682 0.15

SIREVA (Ecuador) | 1.613 0.227 11.451 0.15

SIREVA (El Salvador) | 0.002 0.000 1.9e+35 0.00

SIREVA (Mexico) | 2.857 0.921 8.859 0.21

SIREVA (Panama) | 0.001 0.000 8.6e+34 0.00

SIREVA (Peru) | 0.002 0.000 2.5e+35 0.00

SIREVA (Uruguay) | 1.042 0.147 7.395 0.15

Sub-total |

D+L pooled ES | 1.347 0.782 2.320 1.23

---------------------+---------------------------------------------------

11A Latin America

SIREVA (Brasil) | 1.923 0.962 3.845 0.24

SIREVA (Chile) | 1.190 0.298 4.760 0.19

SIREVA (Colombia) | 2.885 1.296 6.421 0.23

SIREVA (Costa Rica) | 5.263 1.316 21.045 0.19

SIREVA (Ecuador) | 1.613 0.227 11.451 0.15

SIREVA (El Salvador) | 0.002 0.000 1.9e+35 0.00

SIREVA (Mexico) | 1.905 0.476 7.616 0.19

SIREVA (Panama) | 1.471 0.207 10.440 0.15

SIREVA (Peru) | 0.002 0.000 2.5e+35 0.00

SIREVA (Uruguay) | 2.083 0.521 8.330 0.19

Sub-total |

D+L pooled ES | 2.195 1.471 3.275 1.54

---------------------+---------------------------------------------------

38 Latin America

SIREVA (Brasil) | 0.000 0.000 1.4e+34 0.00

SIREVA (Chile) | 1.786 0.576 5.537 0.21

SIREVA (Colombia) | 0.000 0.000 2.8e+34 0.00

SIREVA (Costa Rica) | 0.001 0.000 1.5e+35 0.00

SIREVA (Ecuador) | 0.001 0.000 9.4e+34 0.00

SIREVA (El Salvador) | 0.002 0.000 1.9e+35 0.00

SIREVA (Mexico) | 0.000 0.000 5.6e+34 0.00

SIREVA (Panama) | 0.001 0.000 8.6e+34 0.00

SIREVA (Peru) | 0.002 0.000 2.5e+35 0.00

SIREVA (Uruguay) | 0.001 0.000 6.1e+34 0.00

Sub-total |

D+L pooled ES | 1.765 0.570 5.467 0.21

---------------------+---------------------------------------------------

33F Latin America

SIREVA (Brasil) | 0.000 0.000 1.4e+34 0.00

SIREVA (Chile) | 2.381 0.894 6.344 0.22

SIREVA (Colombia) | 0.000 0.000 2.8e+34 0.00

SIREVA (Costa Rica) | 0.001 0.000 1.5e+35 0.00

SIREVA (Ecuador) | 0.001 0.000 9.4e+34 0.00

SIREVA (El Salvador) | 0.002 0.000 1.9e+35 0.00

SIREVA (Mexico) | 0.000 0.000 5.6e+34 0.00

SIREVA (Panama) | 0.001 0.000 8.6e+34 0.00

SIREVA (Peru) | 0.002 0.000 2.5e+35 0.00

SIREVA (Uruguay) | 1.042 0.147 7.395 0.15

Sub-total |

D+L pooled ES | 2.005 0.835 4.816 0.37

---------------------+---------------------------------------------------

19A Latin America

SIREVA (Brasil) | 8.413 6.041 11.718 0.26

SIREVA (Chile) | 8.333 4.935 14.071 0.25

SIREVA (Colombia) | 10.096 6.583 15.485 0.26

SIREVA (Costa Rica) | 18.421 8.782 38.641 0.24

SIREVA (Ecuador) | 16.129 8.678 29.977 0.25

SIREVA (El Salvador) | 16.129 6.713 38.751 0.23

SIREVA (Mexico) | 39.048 28.751 53.031 0.26

SIREVA (Panama) | 8.824 3.964 19.640 0.23

SIREVA (Peru) | 17.391 6.527 46.338 0.22

SIREVA (Uruguay) | 3.125 1.008 9.689 0.21

Sub-total |

D+L pooled ES | 12.488 7.715 20.216 2.41

---------------------+---------------------------------------------------

15B Latin America

SIREVA (Brasil) | 0.240 0.034 1.707 0.15

SIREVA (Chile) | 1.190 0.298 4.760 0.19

SIREVA (Colombia) | 2.885 1.296 6.421 0.23

SIREVA (Costa Rica) | 0.001 0.000 1.5e+35 0.00

SIREVA (Ecuador) | 0.001 0.000 9.4e+34 0.00

SIREVA (El Salvador) | 0.002 0.000 1.9e+35 0.00

SIREVA (Mexico) | 4.762 1.982 11.441 0.23

SIREVA (Panama) | 0.001 0.000 8.6e+34 0.00

SIREVA (Peru) | 0.002 0.000 2.5e+35 0.00

SIREVA (Uruguay) | 0.001 0.000 6.1e+34 0.00

Sub-total |

D+L pooled ES | 2.542 1.505 4.291 0.81

---------------------+---------------------------------------------------

3 Latin America

SIREVA (Brasil) | 10.577 7.871 14.213 0.26

SIREVA (Chile) | 4.762 2.381 9.522 0.24

SIREVA (Colombia) | 5.769 3.276 10.159 0.25

SIREVA (Costa Rica) | 5.263 1.316 21.045 0.19

SIREVA (Ecuador) | 3.226 0.807 12.899 0.19

SIREVA (El Salvador) | 0.002 0.000 1.9e+35 0.00

SIREVA (Mexico) | 3.810 1.430 10.150 0.22

SIREVA (Panama) | 1.471 0.207 10.440 0.15

SIREVA (Peru) | 8.696 2.175 34.770 0.19

SIREVA (Uruguay) | 12.500 7.099 22.011 0.25

Sub-total |

D+L pooled ES | 6.719 4.675 9.655 1.95

---------------------+---------------------------------------------------

15B North America

Demczuk (2013) | 2.709 1.816 4.041 0.26

Bruce (2015) | 7.692 2.887 20.496 0.22

Kaplan (2013) | 2.473 1.179 5.188 0.24

Moore (2015) | 7.345 4.265 12.649 0.25

Sub-total |

D+L pooled ES | 4.238 2.294 7.829 0.97

---------------------+---------------------------------------------------

6C North America

Demczuk (2013) | 1.919 1.193 3.086 0.25

Bruce (2015) | 0.001 0.000 1.1e+35 0.00

Kaplan (2013) | 3.534 1.901 6.567 0.25

Moore (2015) | 1.695 0.547 5.255 0.21

Sub-total |

D+L pooled ES | 2.323 1.624 3.322 0.71

---------------------+---------------------------------------------------

15BC North America

Demczuk (2013) | 5.643 4.277 7.446 0.26

Bruce (2015) | 13.462 6.417 28.237 0.24

Kaplan (2013) | 4.947 2.930 8.353 0.25

Moore (2015) | 14.124 9.544 20.903 0.26

Sub-total |

D+L pooled ES | 8.323 4.767 14.530 1.01

---------------------+---------------------------------------------------

35B North America

Demczuk (2013) | 2.144 1.368 3.362 0.25

Bruce (2015) | 5.769 1.861 17.888 0.21

Kaplan (2013) | 0.000 0.000 2.1e+34 0.00

Moore (2015) | 7.910 4.684 13.355 0.25

Sub-total |

D+L pooled ES | 4.455 1.741 11.397 0.72

---------------------+---------------------------------------------------

22F North America

Demczuk (2013) | 6.095 4.668 7.958 0.26

Bruce (2015) | 7.692 2.887 20.496 0.22

Kaplan (2013) | 4.240 2.408 7.467 0.25

Moore (2015) | 11.299 7.290 17.514 0.26

Sub-total |

D+L pooled ES | 6.869 4.535 10.403 0.99

---------------------+---------------------------------------------------

23B North America

Demczuk (2013) | 2.596 1.725 3.906 0.26

Bruce (2015) | 1.923 0.271 13.653 0.15

Kaplan (2013) | 2.120 0.952 4.719 0.23

Moore (2015) | 5.085 2.646 9.773 0.24

Sub-total |

D+L pooled ES | 2.957 1.992 4.391 0.89

---------------------+---------------------------------------------------

9N North America

Demczuk (2013) | 0.677 0.304 1.507 0.23

Bruce (2015) | 0.001 0.000 1.1e+35 0.00

Kaplan (2013) | 1.060 0.342 3.287 0.21

Moore (2015) | 0.000 0.000 3.3e+34 0.00

Sub-total |

D+L pooled ES | 0.786 0.409 1.510 0.45

---------------------+---------------------------------------------------

33F North America

Demczuk (2013) | 1.919 1.193 3.086 0.25

Bruce (2015) | 5.769 1.861 17.888 0.21

Kaplan (2013) | 5.654 3.464 9.229 0.25

Moore (2015) | 9.605 5.971 15.450 0.25

Sub-total |

D+L pooled ES | 4.886 2.217 10.767 0.97

---------------------+---------------------------------------------------

3 North America

Demczuk (2013) | 8.465 6.751 10.615 0.26

Bruce (2015) | 1.923 0.271 13.653 0.15

Kaplan (2013) | 4.594 2.667 7.911 0.25

Moore (2015) | 5.650 3.040 10.500 0.25

Sub-total |

D+L pooled ES | 6.067 3.943 9.334 0.91

---------------------+---------------------------------------------------

pcv7 North America

Demczuk (2013) | 3.273 2.275 4.710 0.26

Bruce (2015) | 3.846 0.962 15.379 0.19

Kaplan (2013) | 3.180 1.655 6.112 0.24

Moore (2015) | 3.955 1.885 8.296 0.24

Sub-total |

D+L pooled ES | 3.371 2.533 4.487 0.93

---------------------+---------------------------------------------------

pcv13 North America

Demczuk (2013) | 55.530 50.834 60.661 0.26

Bruce (2015) | 25.000 14.516 43.055 0.25

Kaplan (2013) | 56.537 48.422 66.013 0.26

Moore (2015) | 19.209 13.725 26.884 0.26

Sub-total |

D+L pooled ES | 37.253 25.360 54.725 1.04

---------------------+---------------------------------------------------

11A North America

Demczuk (2013) | 2.483 1.635 3.771 0.26

Bruce (2015) | 1.923 0.271 13.653 0.15

Kaplan (2013) | 0.000 0.000 2.1e+34 0.00

Moore (2015) | 0.000 0.000 3.3e+34 0.00

Sub-total |

D+L pooled ES | 2.455 1.631 3.694 0.41

---------------------+---------------------------------------------------

pcv10-pcv7 North Ame

Demczuk (2013) | 9.819 7.958 12.116 0.26

Bruce (2015) | 3.846 0.962 15.379 0.19

Kaplan (2013) | 15.194 11.269 20.488 0.26

Moore (2015) | 2.260 0.848 6.021 0.22

Sub-total |

D+L pooled ES | 7.874 4.464 13.891 0.94

---------------------+---------------------------------------------------

10A North America

Demczuk (2013) | 2.483 1.635 3.771 0.26

Bruce (2015) | 3.846 0.962 15.379 0.19

Kaplan (2013) | 0.000 0.000 2.1e+34 0.00

Moore (2015) | 0.000 0.000 3.3e+34 0.00

Sub-total |

D+L pooled ES | 2.574 1.725 3.841 0.45

---------------------+---------------------------------------------------

23A North America

Demczuk (2013) | 2.144 1.368 3.362 0.25

Bruce (2015) | 1.923 0.271 13.653 0.15

Kaplan (2013) | 2.473 1.179 5.188 0.24

Moore (2015) | 0.000 0.000 3.3e+34 0.00

Sub-total |

D+L pooled ES | 2.216 1.520 3.231 0.64

---------------------+---------------------------------------------------

non-pcv North Americ

Demczuk (2013) | 44.470 40.288 49.085 0.26

Bruce (2015) | 75.000 54.797 102.651 0.26

Kaplan (2013) | 43.463 36.422 51.865 0.26

Moore (2015) | 80.791 68.577 95.180 0.26

Sub-total |

D+L pooled ES | 57.831 41.606 80.383 1.05

---------------------+---------------------------------------------------

8 North America

Demczuk (2013) | 1.693 1.021 2.808 0.25

Bruce (2015) | 0.001 0.000 1.1e+35 0.00

Kaplan (2013) | 0.353 0.050 2.509 0.15

Moore (2015) | 0.000 0.000 3.3e+34 0.00

Sub-total |

D+L pooled ES | 1.534 0.940 2.504 0.40

---------------------+---------------------------------------------------

38 North America

Demczuk (2013) | 2.822 1.907 4.176 0.26

Bruce (2015) | 0.001 0.000 1.1e+35 0.00

Kaplan (2013) | 0.000 0.000 2.1e+34 0.00

Moore (2015) | 9.040 5.538 14.755 0.25

Sub-total |

D+L pooled ES | 4.980 1.734 14.300 0.51

---------------------+---------------------------------------------------

12F North America

Demczuk (2013) | 1.467 0.852 2.527 0.25

Bruce (2015) | 7.692 2.887 20.496 0.22

Kaplan (2013) | 0.000 0.000 2.1e+34 0.00

Moore (2015) | 3.955 1.885 8.296 0.24

Sub-total |

D+L pooled ES | 3.303 1.320 8.264 0.71

---------------------+---------------------------------------------------

24F North America

Demczuk (2013) | 0.000 0.000 6.6e+33 0.00

Bruce (2015) | 0.001 0.000 1.1e+35 0.00

Kaplan (2013) | 0.000 0.000 2.1e+34 0.00

Moore (2015) | 0.000 0.000 3.3e+34 0.00

Sub-total |

D+L pooled ES | 0.000 0.000 2.5e+15 0.00

---------------------+---------------------------------------------------

15A North America

Demczuk (2013) | 3.725 2.648 5.239 0.26

Bruce (2015) | 7.692 2.887 20.496 0.22

Kaplan (2013) | 1.060 0.342 3.287 0.21

Moore (2015) | 0.000 0.000 3.3e+34 0.00

Sub-total |

D+L pooled ES | 3.348 1.520 7.373 0.69

---------------------+---------------------------------------------------

15C North America

Demczuk (2013) | 2.935 1.998 4.310 0.26

Bruce (2015) | 5.769 1.861 17.888 0.21

Kaplan (2013) | 2.473 1.179 5.188 0.24

Moore (2015) | 6.780 3.850 11.938 0.25

Sub-total |

D+L pooled ES | 3.931 2.367 6.528 0.96

---------------------+---------------------------------------------------

19A North America

Demczuk (2013) | 32.844 29.279 36.843 0.26

Bruce (2015) | 15.385 7.694 30.764 0.24

Kaplan (2013) | 33.216 27.136 40.657 0.26

Moore (2015) | 7.345 4.265 12.649 0.25

Sub-total |

D+L pooled ES | 20.813 13.273 32.634 1.02

---------------------+---------------------------------------------------

6A North America

Demczuk (2013) | 1.129 0.607 2.098 0.25

Bruce (2015) | 0.001 0.000 1.1e+35 0.00

Kaplan (2013) | 0.353 0.050 2.509 0.15

Moore (2015) | 0.000 0.000 3.3e+34 0.00

Sub-total |

D+L pooled ES | 1.015 0.562 1.833 0.40

---------------------+---------------------------------------------------

22F Western Pacific

Australia Surv | 5.978 3.311 10.795 0.25

Nakano (2015) | 11.111 6.581 18.761 0.25

New Zealand Surv | 3.846 1.240 11.926 0.21

Singapore Surv | 0.001 0.000 9.0e+34 0.00

Sub-total |

D+L pooled ES | 7.381 4.535 12.012 0.71

---------------------+---------------------------------------------------

12F Western Pacific

Australia Surv | 0.000 0.000 3.2e+34 0.00

Nakano (2015) | 0.000 0.000 4.6e+34 0.00

New Zealand Surv | 0.001 0.000 7.5e+34 0.00

Singapore Surv | 0.001 0.000 9.0e+34 0.00

Sub-total |

D+L pooled ES | 0.000 0.000 5.2e+15 0.00

---------------------+---------------------------------------------------

24F Western Pacific

Australia Surv | 0.543 0.077 3.858 0.15

Nakano (2015) | 22.222 15.343 32.185 0.26

New Zealand Surv | 0.001 0.000 7.5e+34 0.00

Singapore Surv | 0.001 0.000 9.0e+34 0.00

Sub-total |

D+L pooled ES | 3.944 0.138 112.374 0.41

---------------------+---------------------------------------------------

pcv10-pcv7 Western P

Australia Surv | 10.870 7.013 16.848 0.26

Nakano (2015) | 2.381 0.768 7.382 0.21

New Zealand Surv | 2.564 0.641 10.253 0.19

Singapore Surv | 0.001 0.000 9.0e+34 0.00

Sub-total |

D+L pooled ES | 4.628 1.532 13.980 0.66

---------------------+---------------------------------------------------

pcv13 Western Pacifi

Australia Surv | 43.478 34.922 54.130 0.26

Nakano (2015) | 28.571 20.609 39.610 0.26

New Zealand Surv | 51.282 37.616 69.912 0.26

Singapore Surv | 86.154 66.302 111.950 0.26

Sub-total |

D+L pooled ES | 48.659 31.636 74.842 1.04

---------------------+---------------------------------------------------

11A Western Pacific

Australia Surv | 0.543 0.077 3.858 0.15

Nakano (2015) | 0.794 0.112 5.634 0.15

New Zealand Surv | 0.001 0.000 7.5e+34 0.00

Singapore Surv | 0.001 0.000 9.0e+34 0.00

Sub-total |

D+L pooled ES | 0.655 0.164 2.616 0.30

---------------------+---------------------------------------------------

pcv7 Western Pacific

Australia Surv | 3.261 1.465 7.258 0.23

Nakano (2015) | 0.794 0.112 5.634 0.15

New Zealand Surv | 7.692 3.456 17.122 0.23

Singapore Surv | 26.154 16.259 42.071 0.25

Sub-total |

D+L pooled ES | 5.810 1.597 21.139 0.87

---------------------+---------------------------------------------------

35B Western Pacific

Australia Surv | 4.348 2.174 8.694 0.24

Nakano (2015) | 3.175 1.191 8.459 0.22

New Zealand Surv | 0.001 0.000 7.5e+34 0.00

Singapore Surv | 0.001 0.000 9.0e+34 0.00

Sub-total |

D+L pooled ES | 3.912 2.222 6.889 0.46

---------------------+---------------------------------------------------

15B Western Pacific

Australia Surv | 2.174 0.816 5.792 0.22

Nakano (2015) | 0.000 0.000 4.6e+34 0.00

New Zealand Surv | 5.128 1.925 13.664 0.22

Singapore Surv | 0.001 0.000 9.0e+34 0.00

Sub-total |

D+L pooled ES | 3.335 1.668 6.669 0.45

---------------------+---------------------------------------------------

15C Western Pacific

Australia Surv | 4.348 2.174 8.694 0.24

Nakano (2015) | 0.000 0.000 4.6e+34 0.00

New Zealand Surv | 0.001 0.000 7.5e+34 0.00

Singapore Surv | 0.001 0.000 9.0e+34 0.00

Sub-total |

D+L pooled ES | 4.341 2.171 8.679 0.24

---------------------+---------------------------------------------------

19A Western Pacific

Australia Surv | 22.826 16.869 30.887 0.26

Nakano (2015) | 24.603 17.302 34.984 0.26

New Zealand Surv | 26.923 17.554 41.293 0.26

Singapore Surv | 50.769 36.093 71.413 0.26

Sub-total |

D+L pooled ES | 29.603 20.323 43.119 1.03

---------------------+---------------------------------------------------

23A Western Pacific

Australia Surv | 0.543 0.077 3.858 0.15

Nakano (2015) | 2.381 0.768 7.382 0.21

New Zealand Surv | 3.846 1.240 11.926 0.21

Singapore Surv | 0.001 0.000 9.0e+34 0.00

Sub-total |

D+L pooled ES | 2.367 1.128 4.964 0.57

---------------------+---------------------------------------------------

9N Western Pacific

Australia Surv | 0.543 0.077 3.858 0.15

Nakano (2015) | 0.000 0.000 4.6e+34 0.00

New Zealand Surv | 2.564 0.641 10.253 0.19

Singapore Surv | 0.001 0.000 9.0e+34 0.00

Sub-total |

D+L pooled ES | 1.525 0.492 4.727 0.34

---------------------+---------------------------------------------------

6A Western Pacific

Australia Surv | 0.543 0.077 3.858 0.15

Nakano (2015) | 0.000 0.000 4.6e+34 0.00

New Zealand Surv | 0.001 0.000 7.5e+34 0.00

Singapore Surv | 0.001 0.000 9.0e+34 0.00

Sub-total |

D+L pooled ES | 0.538 0.076 3.813 0.15

---------------------+---------------------------------------------------

15BC Western Pacific

Australia Surv | 6.522 3.704 11.484 0.25

Nakano (2015) | 0.000 0.000 4.6e+34 0.00

New Zealand Surv | 5.128 1.925 13.664 0.22

Singapore Surv | 0.001 0.000 9.0e+34 0.00

Sub-total |

D+L pooled ES | 6.138 3.760 10.019 0.47

---------------------+---------------------------------------------------

10A Western Pacific

Australia Surv | 2.174 0.816 5.792 0.22

Nakano (2015) | 3.968 1.652 9.534 0.23

New Zealand Surv | 0.001 0.000 7.5e+34 0.00

Singapore Surv | 0.001 0.000 9.0e+34 0.00

Sub-total |

D+L pooled ES | 3.034 1.579 5.831 0.45

---------------------+---------------------------------------------------

15A Western Pacific

Australia Surv | 0.543 0.077 3.858 0.15

Nakano (2015) | 11.111 6.581 18.761 0.25

New Zealand Surv | 0.001 0.000 7.5e+34 0.00

Singapore Surv | 0.001 0.000 9.0e+34 0.00

Sub-total |

D+L pooled ES | 2.954 0.224 38.905 0.40

---------------------+---------------------------------------------------

3 Western Pacific

Australia Surv | 5.978 3.311 10.795 0.25

Nakano (2015) | 0.794 0.112 5.634 0.15

New Zealand Surv | 14.103 7.810 25.465 0.25

Singapore Surv | 9.231 4.147 20.547 0.23

Sub-total |

D+L pooled ES | 7.143 3.435 14.855 0.88

---------------------+---------------------------------------------------

non-pcv Western Paci

Australia Surv | 56.522 46.639 68.499 0.26

Nakano (2015) | 71.429 58.096 87.821 0.26

New Zealand Surv | 48.718 35.449 66.954 0.26

Singapore Surv | 13.846 7.204 26.611 0.24

Sub-total |

D+L pooled ES | 45.925 30.921 68.209 1.03

---------------------+---------------------------------------------------

38 Western Pacific

Australia Surv | 0.543 0.077 3.858 0.15

Nakano (2015) | 0.794 0.112 5.634 0.15

New Zealand Surv | 0.001 0.000 7.5e+34 0.00

Singapore Surv | 0.001 0.000 9.0e+34 0.00

Sub-total |

D+L pooled ES | 0.655 0.164 2.616 0.30

---------------------+---------------------------------------------------

23B Western Pacific

Australia Surv | 4.348 2.174 8.694 0.24

Nakano (2015) | 0.000 0.000 4.6e+34 0.00

New Zealand Surv | 2.564 0.641 10.253 0.19

Singapore Surv | 0.001 0.000 9.0e+34 0.00

Sub-total |

D+L pooled ES | 3.909 2.103 7.264 0.43

---------------------+---------------------------------------------------

6C Western Pacific

Australia Surv | 2.174 0.816 5.792 0.22

Nakano (2015) | 1.587 0.397 6.347 0.19

New Zealand Surv | 7.692 3.456 17.122 0.23

Singapore Surv | 1.538 0.217 10.922 0.15

Sub-total |

D+L pooled ES | 2.983 1.247 7.134 0.80

---------------------+---------------------------------------------------

33F Western Pacific

Australia Surv | 4.891 2.545 9.401 0.24

Nakano (2015) | 0.794 0.112 5.634 0.15

New Zealand Surv | 5.128 1.925 13.664 0.22

Singapore Surv | 0.001 0.000 9.0e+34 0.00

Sub-total |

D+L pooled ES | 4.281 2.435 7.526 0.62

---------------------+---------------------------------------------------

8 Western Pacific

Australia Surv | 0.000 0.000 3.2e+34 0.00

Nakano (2015) | 0.000 0.000 4.6e+34 0.00

New Zealand Surv | 3.846 1.240 11.926 0.21

Singapore Surv | 0.001 0.000 9.0e+34 0.00

Sub-total |

D+L pooled ES | 3.829 1.235 11.868 0.21

---------------------+---------------------------------------------------

10A Eastern Mediterr

Al-Sheikh (2014) | 0.001 0.000 7.5e+34 0.00

Shibl (2012) | 0.000 0.000 5.4e+34 0.00

Sub-total |

D+L pooled ES | 0.001 0.000 4.5e+23 0.00

---------------------+---------------------------------------------------

15B Eastern Mediterr

Al-Sheikh (2014) | 0.001 0.000 7.5e+34 0.00

Shibl (2012) | 0.000 0.000 5.4e+34 0.00

Sub-total |

D+L pooled ES | 0.001 0.000 4.5e+23 0.00

---------------------+---------------------------------------------------

24F Eastern Mediterr

Al-Sheikh (2014) | 0.001 0.000 7.5e+34 0.00

Shibl (2012) | 0.000 0.000 5.4e+34 0.00

Sub-total |

D+L pooled ES | 0.001 0.000 4.5e+23 0.00

---------------------+---------------------------------------------------

12F Eastern Mediterr

Al-Sheikh (2014) | 1.282 0.181 9.102 0.15

Shibl (2012) | 0.000 0.000 5.4e+34 0.00

Sub-total |

D+L pooled ES | 1.277 0.180 9.061 0.15

---------------------+---------------------------------------------------

38 Eastern Mediterra

Al-Sheikh (2014) | 0.001 0.000 7.5e+34 0.00

Shibl (2012) | 0.000 0.000 5.4e+34 0.00

Sub-total |

D+L pooled ES | 0.001 0.000 4.5e+23 0.00

---------------------+---------------------------------------------------

33F Eastern Mediterr

Al-Sheikh (2014) | 0.001 0.000 7.5e+34 0.00

Shibl (2012) | 0.000 0.000 5.4e+34 0.00

Sub-total |

D+L pooled ES | 0.001 0.000 4.5e+23 0.00

---------------------+---------------------------------------------------

23A Eastern Mediterr

Al-Sheikh (2014) | 2.564 0.641 10.253 0.19

Shibl (2012) | 0.000 0.000 5.4e+34 0.00

Sub-total |

D+L pooled ES | 2.559 0.640 10.229 0.19

---------------------+---------------------------------------------------

15C Eastern Mediterr

Al-Sheikh (2014) | 0.001 0.000 7.5e+34 0.00

Shibl (2012) | 0.000 0.000 5.4e+34 0.00

Sub-total |

D+L pooled ES | 0.001 0.000 4.5e+23 0.00

---------------------+---------------------------------------------------

8 Eastern Mediterran

Al-Sheikh (2014) | 1.282 0.181 9.102 0.15

Shibl (2012) | 0.000 0.000 5.4e+34 0.00

Sub-total |

D+L pooled ES | 1.277 0.180 9.061 0.15

---------------------+---------------------------------------------------

35B Eastern Mediterr

Al-Sheikh (2014) | 0.001 0.000 7.5e+34 0.00

Shibl (2012) | 0.000 0.000 5.4e+34 0.00

Sub-total |

D+L pooled ES | 0.001 0.000 4.5e+23 0.00

---------------------+---------------------------------------------------

9N Eastern Mediterra

Al-Sheikh (2014) | 0.001 0.000 7.5e+34 0.00

Shibl (2012) | 0.000 0.000 5.4e+34 0.00

Sub-total |

D+L pooled ES | 0.001 0.000 4.5e+23 0.00

---------------------+---------------------------------------------------

non-pcv Eastern Medi

Al-Sheikh (2014) | 14.103 7.810 25.465 0.25

Shibl (2012) | 5.556 2.496 12.366 0.23

Sub-total |

D+L pooled ES | 9.219 3.713 22.889 0.48

---------------------+---------------------------------------------------

15BC Eastern Mediter

Al-Sheikh (2014) | 0.001 0.000 7.5e+34 0.00

Shibl (2012) | 0.000 0.000 5.4e+34 0.00

Sub-total |

D+L pooled ES | 0.001 0.000 4.5e+23 0.00

---------------------+---------------------------------------------------

6C Eastern Mediterra

Al-Sheikh (2014) | 0.001 0.000 7.5e+34 0.00

Shibl (2012) | 0.000 0.000 5.4e+34 0.00

Sub-total |

D+L pooled ES | 0.001 0.000 4.5e+23 0.00

---------------------+---------------------------------------------------

3 Eastern Mediterran

Al-Sheikh (2014) | 2.564 0.641 10.253 0.19

Shibl (2012) | 1.852 0.463 7.405 0.19

Sub-total |

D+L pooled ES | 2.179 0.818 5.806 0.38

---------------------+---------------------------------------------------

22F Eastern Mediterr

Al-Sheikh (2014) | 0.001 0.000 7.5e+34 0.00

Shibl (2012) | 0.000 0.000 5.4e+34 0.00

Sub-total |

D+L pooled ES | 0.001 0.000 4.5e+23 0.00

---------------------+---------------------------------------------------

23B Eastern Mediterr

Al-Sheikh (2014) | 1.282 0.181 9.102 0.15

Shibl (2012) | 0.000 0.000 5.4e+34 0.00

Sub-total |

D+L pooled ES | 1.277 0.180 9.061 0.15

---------------------+---------------------------------------------------

19A Eastern Mediterr

Al-Sheikh (2014) | 3.846 1.240 11.926 0.21

Shibl (2012) | 7.407 3.704 14.812 0.24

Sub-total |

D+L pooled ES | 6.195 3.431 11.186 0.45

---------------------+---------------------------------------------------

pcv7 Eastern Mediter

Al-Sheikh (2014) | 73.077 56.368 94.739 0.26

Shibl (2012) | 53.704 41.518 69.466 0.26

Sub-total |

D+L pooled ES | 62.615 46.300 84.679 0.52

---------------------+---------------------------------------------------

pcv13 Eastern Medite

Al-Sheikh (2014) | 85.897 67.606 109.137 0.26

Shibl (2012) | 94.444 77.785 114.673 0.26

Sub-total |

D+L pooled ES | 90.959 78.229 105.760 0.52

---------------------+---------------------------------------------------

pcv10-pcv7 Eastern M

Al-Sheikh (2014) | 2.564 0.641 10.253 0.19

Shibl (2012) | 25.000 17.144 36.455 0.26

Sub-total |

D+L pooled ES | 8.863 0.960 81.832 0.45

---------------------+---------------------------------------------------

11A Eastern Mediterr

Al-Sheikh (2014) | 0.001 0.000 7.5e+34 0.00

Shibl (2012) | 0.000 0.000 5.4e+34 0.00

Sub-total |

D+L pooled ES | 0.001 0.000 4.5e+23 0.00

---------------------+---------------------------------------------------

6A Eastern Mediterra

Al-Sheikh (2014) | 2.564 0.641 10.253 0.19

Shibl (2012) | 6.481 3.090 13.596 0.24

Sub-total |

D+L pooled ES | 4.942 2.162 11.297 0.43

---------------------+---------------------------------------------------

15A Eastern Mediterr

Al-Sheikh (2014) | 0.001 0.000 7.5e+34 0.00

Shibl (2012) | 0.000 0.000 5.4e+34 0.00

Sub-total |

D+L pooled ES | 0.001 0.000 4.5e+23 0.00

---------------------+---------------------------------------------------
