# Supplementary material for: Serotype distribution of Streptococcus pneumoniae causing invasive disease in children in the post-PCV era: A systematic review and meta-analysis
Source: PLoS One. 2017 May 9;12(5):e0177113. doi: 10.1371/journal.pone.0177113 (PMC5423631; doi:10.1371/journal.pone.0177113)
Supplement: S6 Table — (DOCX) [file pone.0177113.s007.docx]

# **Serotype distribution of Streptococcus pneumoniae causing invasive disease in young children during the post-PCV period**

Evelyn Balsells, Laurence Guillot, Harish Nair, Moe H. Kyaw

## S6 Table. Sensitivity analysis

**Exclusion of studies including children aged more than 5 years**

|  | **Studies included in meta-analysis (<18 years)** | **Sensitivity analysis results**  **(any age group < 5 years)** |  |
| --- | --- | --- | --- |
| **PCV 7** |  |  |  |
|  | **Overall % (95%CI)** | **Overall % (95%CI)** |  |
| pcv7 | 14.8 (11.4-19.1) | 14.6 (10.8-19.7) |  |
| pcv10not7 | 16.3 (13.5-19.8) | 17.4 (14.3-21.2) |  |
| pcv13 | 70.5 (67.6-73.5) | 70.8 (67.7-74) |  |
| 19A | 21.8 (18.6-25.6) | 22.6 (18.9-27) |  |
| 3 | 4.9 (4.2-5.8) | 5 (4.2-6) |  |
| 6A | 3.1 (2.4-4.1) | 3.2 (2.5-4.2) |  |
| non pcv13 | 29.4 (26.8-32.4) | 29.4 (26.5-32.6) |  |
| 22F | 3.5 (2.9-4.3) | 3.4 (2.7-4.3) |  |
| 12F | 2.3 (1.8-2.9) | 2.2 (1.7-3) |  |
| 33F | 3.4 (2.6-4.6) | 3.5 (2.5-4.9) |  |
| 24F | 4 (3.4-4.8) | 4.1 (3.5-5) |  |
| 15C | 2.8 (2.3-3.4) | 2.6 (2.1-3.2) |  |
| 15B | 2.4 (2-3) | 2.4 (2-3) |  |
| 23B | 1.7 (1.4-2.1) | 1.7 (1.4-2.2) |  |
| 10A | 2.6 (2-3.3) | 2.8 (2.1-3.7) |  |
| 15A | 1.8 (1.2-2.7) | 1.5 (1.1-2.2) |  |
| 38 | 2.3 (1.8-2.8) | 2.3 (1.8-2.9) |  |
| 35B | 1.2 (0.9-1.6) | 0.8 (0.5-1.2) |  |
| 6C | 2.8 (2.2-3.4) | 2.2 (1.7-2.9) |  |
| 8 | 1.4 (1.1-1.9) | 1.5 (1.1-2) |  |
| 11A | 1.3 (1-1.8) | 1.3 (1-1.8) |  |
| 23A | 1.6 (1.2-2.1) | 1.1 (0.8-1.6) |  |
| 9N | 0.9 (0.6-1.2) | 0.9 (0.6-1.3) |  |
| **In countries that have introduced higher valent PCVs** | | |  |
|  | **Overall % (95%CI)** | **Overall % (95%CI)** |  |
| pcv7 | 12.5 (8.8-17.7) | 12.2 (8.6-17.5) |  |
| pcv10not7 | 9.2 (6.9-12.2) | 9.9 (7.4-13.2) |  |
| pcv13 | 49.1 (42.3-56.9) | 49 (42.1-57) |  |
| 19A | 14.2 (11.1-18.3) | 14.4 (11-18.7) |  |
| 3 | 5.3 (4.2-6.7) | 5.5 (4.4-7) |  |
| 6A | 3.2 (2.1-4.9) | 3.2 (2.1-5.1) |  |
| non-pcv13 | 42.2 (36.1-49.5) | 42.7 (36.3-50.2) |  |
| 22F | 5.3 (4.2-6.7) | 5 (4-6.3) |  |
| 12F | 4.3 (3.5-5.3) | 4.4 (3.6-5.4) |  |
| 33F | 4.5 (3.4-5.9) | 4.6 (3.5-6) |  |
| 24F | 4.2 (2.6-6.8) | 3.5 (2.1-5.8) |  |
| 15C | 4 (3.1-5) | 3.9 (3-5.1) |  |
| 15B | 3.7 (3.1-4.4) | 3.7 (3.1-4.4) |  |
| 23B | 3.4 (2.6-4.3) | 3.4 (2.6-4.3) |  |
| 10A | 3.4 (2.2-5.4) | 3.4 (2.1-5.4) |  |
| 15A | 2.9 (1.9-4.4) | 2.6 (1.7-3.9) |  |
| 38 | 3.4 (2.4-4.8) | 3.6 (2.6-5.1) |  |
| 35B | 2.6 (1.8-3.8) | 2.5 (1.6-3.8) |  |
| 6C | 2.4 (1.8-3) | 2.4 (1.8-3.1) |  |
| 8 | 2.2 (1.3-3.8) | 2.3 (1.3-4) |  |
| 11A | 2 (1.6-2.6) | 2.1 (1.6-2.6) |  |
| 23A | 2 (1.6-2.6) | 2 (1.5-2.6) |  |
| 9N | 1.3 (1-1.8) | 1.3 (1-1.8) |  |
